# Population genomics of Mesolithic Scandinavia: Investigating early postglacial migration routes and high-latitude adaptation

**DOI:** 10.1371/journal.pbio.2003703

**Published:** 2018-01-09

**Authors:** Torsten Günther, Helena Malmström, Emma M. Svensson, Ayça Omrak, Federico Sánchez-Quinto, Gülşah M. Kılınç, Maja Krzewińska, Gunilla Eriksson, Magdalena Fraser, Hanna Edlund, Arielle R. Munters, Alexandra Coutinho, Luciana G. Simões, Mário Vicente, Anders Sjölander, Berit Jansen Sellevold, Roger Jørgensen, Peter Claes, Mark D. Shriver, Cristina Valdiosera, Mihai G. Netea, Jan Apel, Kerstin Lidén, Birgitte Skar, Jan Storå, Anders Götherström, Mattias Jakobsson

**Affiliations:** 1 Department of Organismal Biology, Uppsala University, Uppsala, Sweden; 2 Department of Archaeology and Classical Studies, Stockholm University, Stockholm, Sweden; 3 Middle East Technical University, Department of Biological Sciences, Ankara, Turkey; 4 Department of Archaeology and Ancient History, Uppsala University-Campus Gotland, Visby, Sweden; 5 Norwegian Institute for Cultural Heritage Research, Oslo, Norway; 6 Tromsø University Museum, University of Tromsø-The Arctic University of Norway, Tromsø, Norway; 7 Department of Electrical Engineering, Center for Processing Speech and Images, Katholieke Universiteit Leuven, Leuven, Belgium; 8 Department of Anthropology, Penn State University, State College, Pennsylvania, United States of America; 9 Department of Archaeology and History, La Trobe University, Melbourne, Australia; 10 Department of Internal Medicine and Radboud Center for Infectious Diseases, Radboud University Medical Center, Nijmegen, the Netherlands; 11 Department of Archaeology and Ancient History, Lund University, Lund, Sweden; 12 Department of Archaeology and Cultural History, Norwegian University of Science and Technology University Museum, Trondheim, Norway; 13 SciLifeLab, Uppsala and Stockholm, Sweden; The Institute of Science and Technology Austria, Austria

## Abstract

Scandinavia was one of the last geographic areas in Europe to become habitable for humans after the Last Glacial Maximum (LGM). However, the routes and genetic composition of these postglacial migrants remain unclear. We sequenced the genomes, up to 57× coverage, of seven hunter-gatherers excavated across Scandinavia and dated from 9,500–6,000 years before present (BP). Surprisingly, among the Scandinavian Mesolithic individuals, the genetic data display an east–west genetic gradient that opposes the pattern seen in other parts of Mesolithic Europe. Our results suggest two different early postglacial migrations into Scandinavia: initially from the south, and later, from the northeast. The latter followed the ice-free Norwegian north Atlantic coast, along which novel and advanced pressure-blade stone-tool techniques may have spread. These two groups met and mixed in Scandinavia, creating a genetically diverse population, which shows patterns of genetic adaptation to high latitude environments. These potential adaptations include high frequencies of low pigmentation variants and a gene region associated with physical performance, which shows strong continuity into modern-day northern Europeans.

## Author summary

The Scandinavian peninsula was the last part of Europe to be colonized after the Last Glacial Maximum. The migration routes, cultural networks, and the genetic makeup of the first Scandinavians remain elusive and several hypotheses exist based on archaeology, climate modeling, and genetics. By analyzing the genomes of early Scandinavian hunter-gatherers, we show that their migrations followed two routes: one from the south and another from the northeast along the ice-free Norwegian Atlantic coast. These groups met and mixed in Scandinavia, creating a population more diverse than contemporaneous central and western European hunter-gatherers. As northern Europe is associated with cold and low light conditions, we investigated genomic patterns of adaptation to these conditions and genes known to be involved in skin pigmentation. We demonstrate that Mesolithic Scandinavians had higher levels of light pigmentation variants compared to the respective source populations of the migrations, suggesting adaptation to low light levels and a surprising signal of genetic continuity in *TMEM131*, a gene that may be involved in long-term adaptation to the cold.

## Introduction

**Fig 1:**
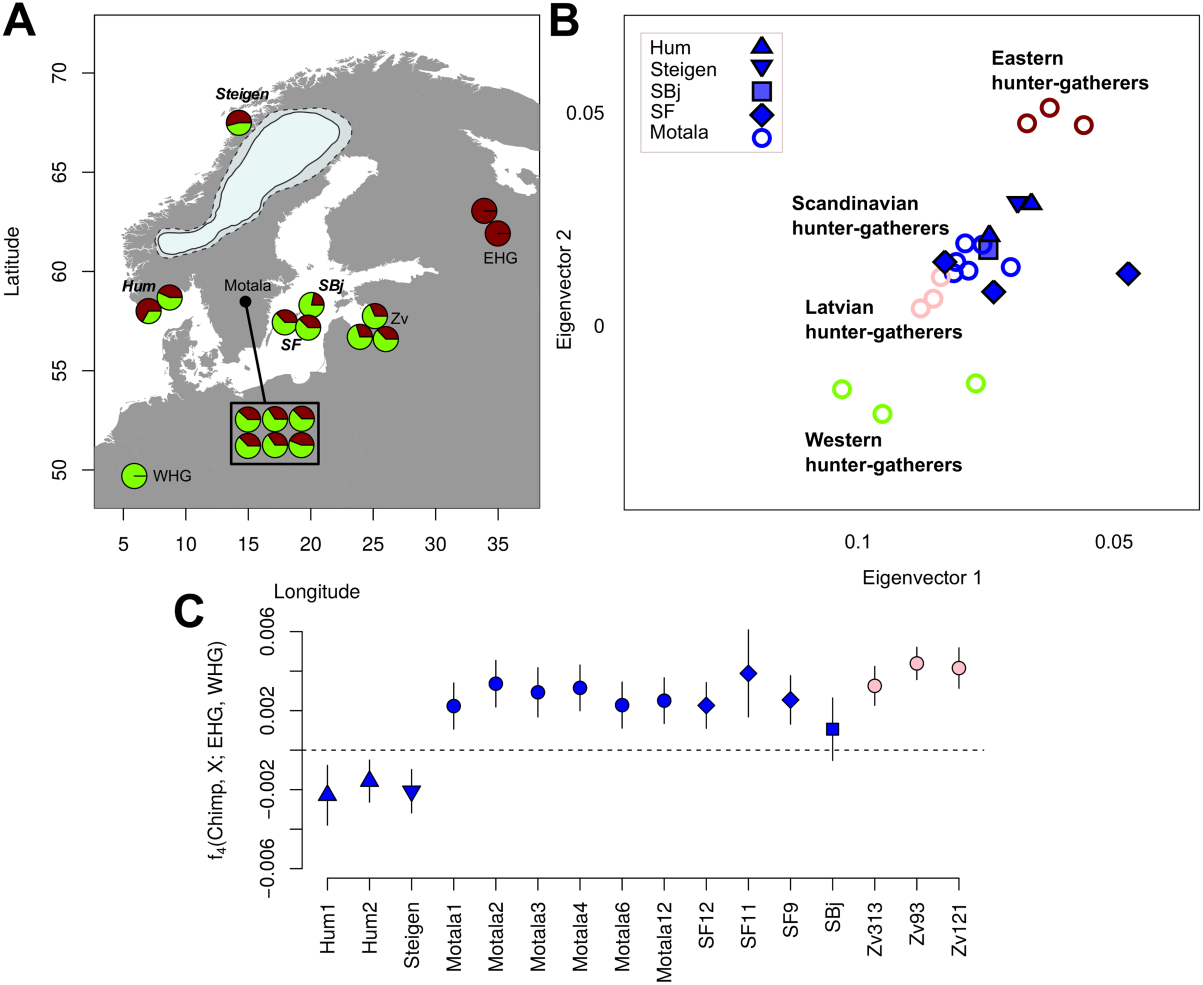
Mesolithic samples and their genetic affinities. (A) Map of the Mesolithic European samples used in this study. The pie charts show the model-based [18,19] estimates of genetic ancestry for each SHG individual. The map also displays the ice sheet covering Scandinavia 10,000 cal BP (most credible [solid line] and maximum extend [dashed line] following [10]). Newly sequenced individuals are shown with bold and italic site names. SF11 is excluded from this map due to its low coverage (0.1×). Additional European EHG and WHG individuals used in this study derive from sites outside this map. The map was plotted using the R package rworldmap [28]. (B) Magnified section of genetic similarity among ancient and modern day individuals using PCA, featuring only the Mesolithic European samples (see S6 Text for the full plot). Symbols representing newly sequenced individuals have a black contour line. (C) Allele sharing between the SHGs, Latvian Mesolithic hunter-gatherers (Zv) [29], and EHGs versus WHGs measured by the statistic f_4_(Chimp, SHG; EHG, WHG) calculated for the captured SNPs [20]. Error bars show two block-jackknife standard errors. Data shown in this figure can be found in S1 Data. BP, before present; cal, calibrated; Chimp, Chimpanzee; EHG, eastern hunter-gatherer; PCA, principal component analysis; SHG, Scandinavian hunter-gatherer; WHG, western hunter-gatherer; Zv, Latvian Mesolithic hunter-gatherer from Zvejnieki.

As the ice sheet retracted from northern Europe after the Last Glacial Maximum (LGM), around 23,000 years ago, new habitable areas emerged [1], allowing plants [2,3] and animals [4,5] to recolonize the Scandinavian peninsula (hereafter referred to as Scandinavia). There is consistent evidence of human presence in the archaeological record from approximately 11,700 years before present (BP) both in southern and northern Scandinavia [6–9]. At this time, the ice sheet was still dominating the interior of Scandinavia [9,10] (Fig 1A, S1 Text), but recent climate modeling shows that the Arctic coast of (modern-day) northern Norway was ice free [10]. Similarities in late-glacial lithic technology (direct blade percussion technique) of Western Europe and the oldest counterparts of Scandinavia appearing around 11,000 calibrated (cal) BP [11] (S1 Text) have been used to argue for an early postglacial migration from southwestern Europe into Scandinavia, including areas of northern Norway. However, studies of another lithic technology, the “pressure blade” technique, which first occurred in the northern parts of Scandinavia around 10,200 cal BP, indicates contact with groups in the east and possibly an eastern origin of the early settlers [7,12–15] (S1 Text). The first genetic studies of Mesolithic human remains from central and eastern Scandinavian hunter-gatherers (SHGs) revealed similarities to two different Mesolithic European populations, the “western hunter-gatherers” (WHGs) from western, central, and southern Europe and the “eastern hunter-gatherers” (EHGs) from northeastern and eastern Europe [16–24]. Archaeology, climate modeling, and genetics suggest several possibilities for the early postglacial migrations into Scandinavia, including migrations from the south, southeast, northeast, and combinations of these; however, the early postglacial peopling of Scandinavia remains elusive [1,4,6–19,25,26]. In this study, we contrast genome sequence data and stable isotopes from Mesolithic human remains from western, northern, and eastern Scandinavia to infer the early postglacial migration routes into Scandinavia—from where people came, what routes they followed, how they were related to other Mesolithic Europeans [17–21,27]—and to investigate human adaptation to high-latitude environments.

## Results and discussion

We sequenced the genomes of seven hunter-gatherers from Scandinavia (Table 1; S1, S2 and S3 Text) ranging from 57.8× to 0.1× genome coverage, of which four individuals had a genome coverage above 1×. The remains were directly dated to between 9,500 cal BP and 6,000 cal BP, and were excavated in southwestern Norway (Hum1, Hum2), northern Norway (Steigen), and the Baltic islands of Stora Karlsö and Gotland (SF9, SF11, SF12, and SBj), and represent 18% (6 of 33) of all known human remains in Scandinavia older than 8,000 years [30]. All samples displayed fragmentation and cytosine deamination at fragment termini characteristic for ancient DNA (aDNA) (S3 Text). Mitochondrial (mt) DNA-based contamination estimates were <6% for all individuals (confidence intervals ranging from 0% to 9.5%) and autosomal contamination was <1% for all individuals except for SF11, which showed approximately 10% contamination (Table 1, S4 Text). Four of the seven individuals were inferred to be males and three were females. All the western and northern Scandinavian individuals and one eastern Scandinavian carried U5a1 mt haplotypes, whereas the remaining eastern Scandinavians carried U4a haplotypes (Table 1, S5 Text). These individuals represent the oldest U5a1 and U4 lineages detected so far. The Y chromosomal haplotype was determined for three of the four males, all carried I2 haplotypes, which were common in pre-Neolithic Europe (Table 1, S5 Text).

**Table 1:** Information on the seven SHGs investigated in this study, including cal BP (corrected for the marine reservoir effect, given as a range of two standard deviations), average genome coverage, average mt coverage, mt and Y chromosome haplogroups, and contamination estimates based on the mt, the X-chromosome for males and the autosomes.

Individual	Calibrated Date (cal BP, 2 sigma)	Genome Coverage	mt Coverage	Sex	mt Haplo-Group	Y Haplo-Group	Contamination Estimate		
							Based on mt	Based on X	Based on Autosomes
Hum1	9,452–9,275^$^	0.71	597	XX	U5a1	-	0.29%	-	0.00%
Hum2	9,452–9,275^$^	4.05	432	XY	U5a1d	I2-M438	0.15%	0.63%	0.73%
Steigen	5,950–5,764	1.24	277	XY	U5a1d	I2a1b-M423	0.00%	0.4%	0.00%
SF9	9,300–8,988	1.15	93	XX	U4a2	-	5.36%	-	0.00%
SF11	9,023–8,760	0.10	45	XY	U5a1	*	3.42%	*	10.16%
SF12	9,033–8,757	57.79	9774	XX	U4a1	-	0.34%	-	0.93%
SBj	8,963–8,579	0.43	102	XY	U4a1	I2-L68	3.72%	1.4%	0.06%

^$^Combined probability for the Hummervikholmen samples.

*Not enough genome coverage.

BP, before present; cal, calibrated; mt, mitochondrial; SHG, Scandinavian hunter-gatherer.

The high coverage and Uracil-DNA-glycosylase (UDG)-treated genome (used in order to reduce the effects of postmortem DNA damage) [31] of SF12 allowed us to confidently discover new and hitherto unknown variants at sites with 55× or higher sequencing depth (S3 Text). Based on SF12’s high-coverage and high-quality genome, we estimate the number of SNPs hitherto unknown (not recorded in dbSNP [v142]) to be approximately 10,600. This number is close to the median per European individual in the 1000 Genomes Project [32] (approximately 11,400, S3 Text), although a direct comparison is difficult due to the lower sequencing depth, different data processing, and larger sample sizes in the 1000 Genomes Project. At least 17% of these SNPs that are not found in modern-day individuals were in fact common among the Mesolithic Scandinavians (seen in the low coverage data conditional on the observation in SF12), and in total 24.2% were found in other prehistoric individuals (S3 Text), suggesting a substantial amount of hitherto unknown variation 9,000 years ago (S3 Text). Thus, many genetic variants found in Mesolithic individuals have not been carried over to modern-day groups. Among the novel variants in SF12, four (all heterozygous) are predicted to affect the function of protein coding genes [33] (S3 Text). The “heat shock protein” *HSPA2* in SF12 carries an unknown mutation that changes the amino acid histidine to tyrosine at a protein–protein interaction site, which likely disrupts the function of the protein (S3 Text). Defects in *HSPA2* are known to drastically reduce fertility in males [34]. It will be interesting to see how common such variants were among Mesolithic groups as more genome sequence data become available. The genomic data further allowed us to study the physical appearance of SHGs (S8 Text); for instance, they show a combination of eye color varying from blue to light brown and light skin pigmentation. This is strikingly different from the WHGs—who have been suggested to have the specific combination of blue eyes and dark skin [18,20,21,23] and EHGs—who have been suggested to be brown-eyed and light-skinned [19,20].

### Demographic history of Mesolithic Scandinavians

In order to compare the genomic sequence data of the seven SHGs to genetic information from other ancient individuals and modern-day groups, data were merged with shotgun sequence data and SNP capture data from six published Mesolithic individuals from Motala in central Scandinavia, and 47 published Stone Age (Upper Paleolithic, Mesolithic, and Early Neolithic) individuals from other parts of Eurasia (S6 Text) [17–22,26,27,29,35–38], as well as with a world-wide set of 203 modern-day populations [18,32,39]. All 13 SHGs—regardless of geographic sampling location and age—display genetic affinities to both WHGs and EHGs (Fig 1A and 1B, S6 Text). One individual, SF11, seems to be a slight genetic outlier in the principal component analysis (PCA), which could be due to the lower coverage or driven by nuclear contamination (Table 1, S6 Text). Generally, the pattern of dual ancestry is consistent with a scenario in which SHGs represent a mixed group tracing parts of their ancestry to both the WHGs and the EHGs [17–19,22,24,40].

The SHGs from northern and western Scandinavia show a distinct and significantly stronger affinity to the EHGs compared to the central and eastern SHGs (Fig 1). Conversely, the SHGs from eastern and central Scandinavia were genetically more similar to WHGs compared to the northern and western SHGs (Fig 1). Using *qpAdm* [19], the EHG genetic component of northern and western SHGs was estimated to 48.9% (± 5%) and differs from the 37.8% (± 3.2%) observed in eastern and south-central SHGs. The latter estimate is similar to ancestry estimates obtained for eastern Baltic hunter-gatherers from Latvia [29] (33.7% ± 4.7%, Fig 1A). Although the difference in ancestry estimates between northern and western SHG, and eastern and south-central SHG is only marginally significant (Z = 1.87, *p* = 0.062), this pattern is in agreement with other analyses such as ADMIXTURE and TreeMix (S6 Text). Furthermore, the direct comparison using D statistics with Chimpanzee (Chimp) as an outgroup (D(Chimp, WHG; eastern or south-central SHG, northern or western SHG) < 0, Z = −5.14 and D(Chimp, EHG; eastern or south-central SHG, northern or western SHG) > 0, Z = 1.72) show that WHG are genetically closer to eastern and south-central SHG, whereas EHG tend to share more alleles with northern and western SHGs (S2 Fig). These patterns of genetic affinity within SHGs are in direct contrast to the expectation based on geographic proximity with EHGs and WHGs.

**Fig 2:**
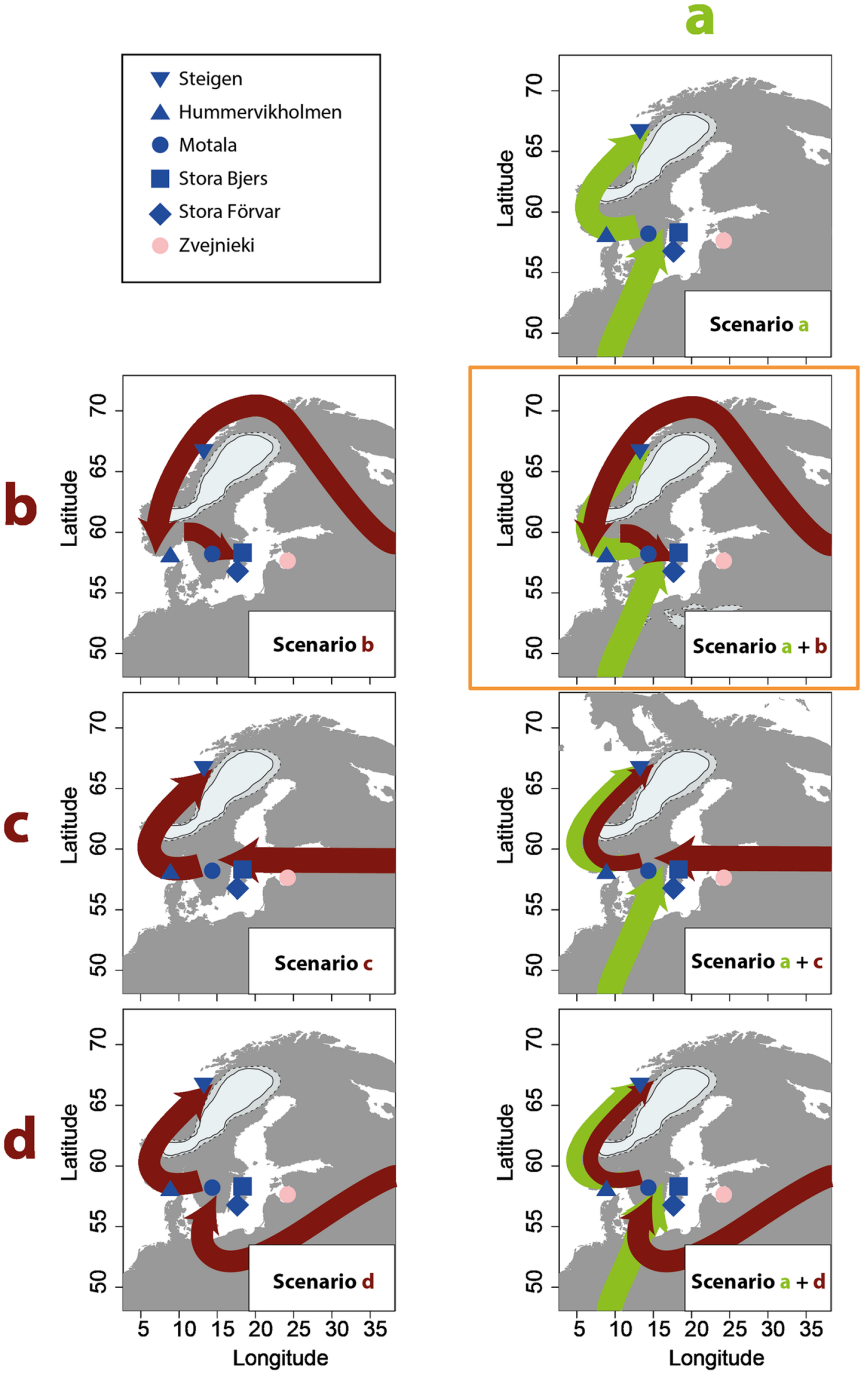
Migration scenarios into postglacial Scandinavia. Maps showing potential migration routes into Scandinavia. Scenario (a) shows a migration related to the Ahrensburgian tradition from the south (S1 Text). Scenarios (b), (c), and (d) show different possible routes into Scandinavia for the EHG ancestry. The scenarios are discussed in the text and the scenario most consistent with genetic data and stone tools is a combination of routes (a) and (b). All maps were plotted using the R package rworldmap [28]. EHG, eastern hunter-gatherer.

From about 11,700 cal BP, consistent archaeological evidence of human presence exists in southern Scandinavia following the retreat of the ice sheet [6,41,42] (S1 Text). Artifacts and tools found at these sites show similarities with the Ahrensburgian tradition of northern central Europe [15,43], suggesting that these hunter-gatherers likely had a southern origin from a WHG-like gene pool as no EHG ancestry has been found in central and western Europe [18,21,24,27]. Although this genetic component would have entered from today’s northern Germany and Denmark (Fig 2, Scenario a), it remains unclear how and where the EHG component entered Scandinavia (Fig 2, Scenarios b, c and/or d). The EHG-related migration likely took place after the migration of WHGs from the south as the earliest eastern-associated pressure blade finds postdate the southwestern-associated direct blade finds in Scandinavia (S1 Text). Two migrations with admixture at different time-periods would generate a genetic gradient with the highest contribution of a source close to its geographic region of entry. The observed genetic pattern is consistent with a migration of the EHGs from the northeast moving southwards along the ice-free Norwegian Atlantic coast where the two groups started mixing (Fig 2, Scenarios a and b), which would cause more EHG ancestry in western SHGs. If the EHG migration had crossed the Baltic Sea into Scandinavia, where it would meet and mix with a WHG-like population (Fig 2, combination of Scenarios a and c), a gradient with most EHG ancestry in eastern SHGs would have been created—exactly opposite to the observed pattern. A similar pattern would be expected if the EHG migration went around the Baltic Sea along current day’s Finnish west coast and down via today’s Swedish east coast (scenario not depicted in Fig 2). An EHG migration along the southern Baltic coast (Fig 2, Scenarios a and d) should cause a related pattern to a crossing of the Baltic Sea with more EHG ancestry in central and eastern SHGs. Furthermore, such a scenario would likely also make the Latvian Mesolithic hunter-gatherers the group with most EHG ancestry, which is in stark contrast to the empirical data in which the Latvian group shows the lowest proportion of EHG ancestry (33.7% ± 4.7%), and also not consistent with chronology, as the dated settlements east of the Baltic Sea are younger than the early settlements in Scandinavia (S1 Text). Thus, the only scenario consistent with both genetic and archaeological data is a migration of a WHG-related group migrating into Scandinavia from the south, followed by an EHG-related group migrating to Scandinavia from the northeast along the Norwegian Atlantic coast. Notably, such a migration along the Norwegian coast could have been facilitated by the use of the more specialized pressure blade technique (S1 Text) [12,14]. The individuals sequenced here postdate these migrations, but a genetic east-west gradient would be maintained over time in Scandinavia and only additional large-scale migrations from different sources would alter this pattern. This observation is important as the geographic pattern still holds without the chronologically much younger Steigen individual, which might represent local continuity or later migrations into north-western Scandinavia from the east.

Interestingly, stable nitrogen and carbon isotope analysis of northern and western SHGs revealed an extreme marine diet, suggesting a pronounced maritime subsistence, in contrast to the more mixed terrestrial and aquatic diet of eastern and central SHGs (S1 Text). Mobility is difficult to trace based solely on carbon and nitrogen isotope data; however, the patterns are consistent with a migration along the Norwegian Atlantic coast relying on local resources.

### Genetic diversity in Mesolithic Scandinavia

**Fig 3:**
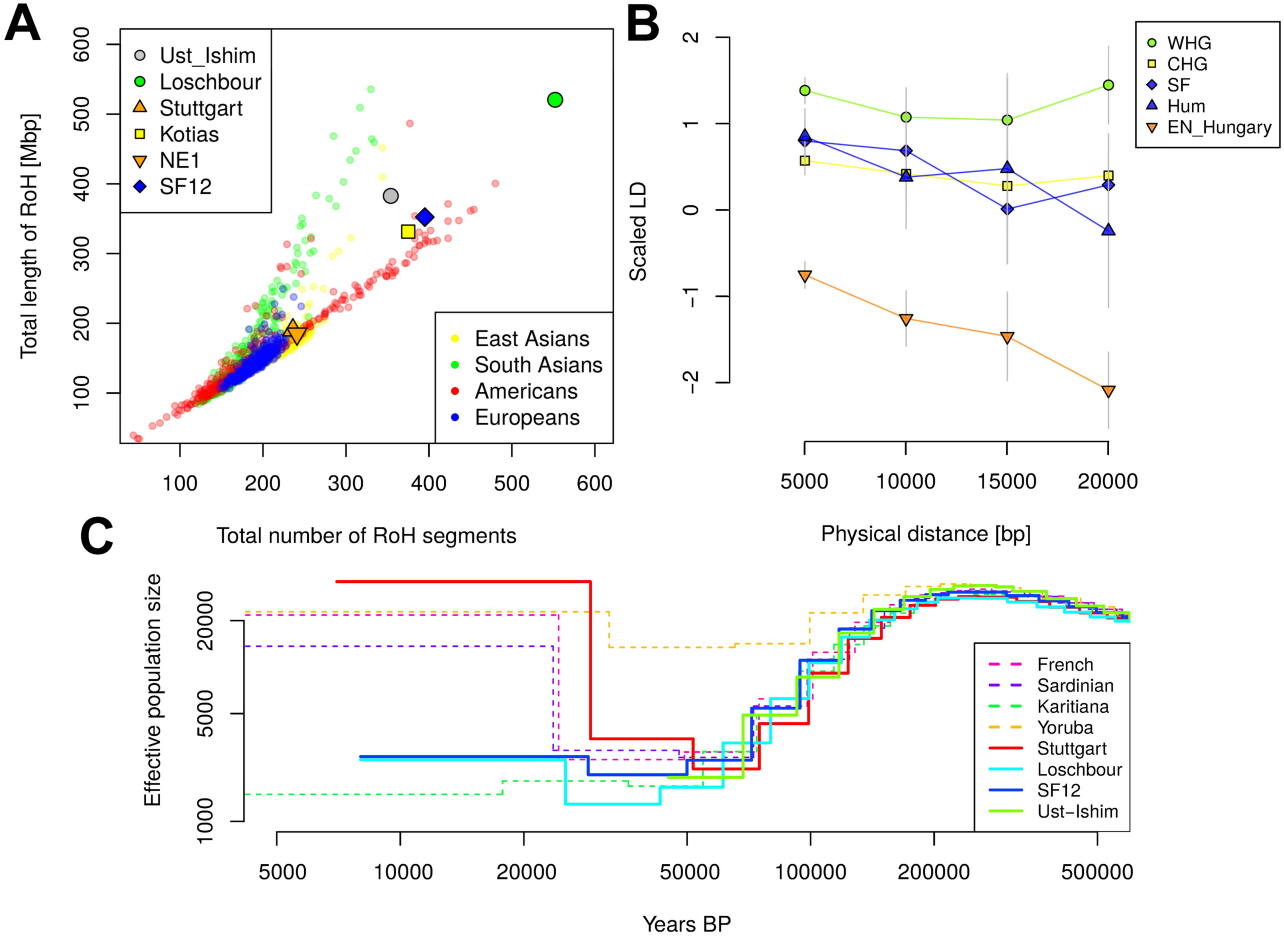
Genetic diversity in prehistoric Europe. (A) RoH for the six prehistoric humans that have been sequenced to >15× genome coverage, (Kotias is a hunter-gatherer from the Caucasus region [35], NE1 is an early Neolithic individual from modern-day Hungary [27], the other individuals are described in the text), compared to all modern-day non African individuals from the 1000 Genomes Project [32]. (B) LD decay for five prehistoric populations each represented by two individuals (eastern SHGs: SF [SF9 and SF12], western SHGs: Hum [Hum1 and Hum2], CHGs [35]: [Kotias and Satsurblia], WHGs [18,35] [Loschbour and Bichon], and early Neolithic Hungarians [27]: EN_Hungary [NE1 and NE6]). LD was scaled in each distance bin by using the LD for two modern populations [32] as 0 (TSI) and as 1 (PEL). LD was calculated from the covariance of derived allele frequencies of two haploid individuals per population (S7 Text). Error bars show two standard errors estimated during 100 bootstraps across SNP pairs. (C) Effective population size over time as inferred by PSMC’ [44] for four prehistoric humans with high genome coverage. The dashed lines show the effective population sizes for selected modern-day populations. All curves for prehistoric individuals were shifted along the x-axis according to their radiocarbon date. S4 Fig. shows 100 bootstrap replicates per individual. Data shown in this figure can be found in S1 Data. BP, before present; CHG, Caucasus hunter-gatherer; LD, linkage disequilibrium; PEL, modern-day Peruvian individual; PSMC’, pairwise sequentially Markovian coalescent; RoH, runs of homozygosity; SHG, Scandinavian hunter-gatherer; TSI, modern-day Tuscan individual; WHG, western hunter-gatherer.

By sequencing complete ancient genomes, we can compute unbiased estimates of genetic diversity, which are informative of past population sizes and population history. Here, we restrict the analysis to WHGs and SHGs because only SNP capture data are available for EHGs (S7 Text). In modern-day Europe, there is greater genetic diversity in the south compared to the north. During the Mesolithic period, by contrast, we find lower levels of runs of homozygosity (RoH) (Fig 3A) and linkage disequilibrium (LD) (Fig 3B) in SHGs compared to WHGs (represented by Loschbour and Bichon [18,35]). By using a multiple sequentially Markovian coalescent (MSMC) approach [44] for the high-coverage, high-quality genome of SF12, we find that right before the SF12 individual lived, the effective population size of SHGs was similar to that of WHGs (Fig 3C). At the time of the LGM and back to approximately 50,000 years ago, both the WHGs and SHGs go through a bottleneck, but the ancestors of SHGs retained a greater effective population size in contrast to the ancestors of WHGs who went through a more severe bottleneck (Fig 2C), which is consistent across 100 bootstrap replicates (S2 Fig). These differences in effective population size estimates may be attributed to the admixture in SHGs as migration events can have delayed effects on estimates of effective population size over time [45]. Around 50,000–70,000 years ago, the effective population sizes of the ancestors of SHGs, WHGs, Neolithic groups (represented by Stuttgart [18]), and Paleolithic Eurasians (represented by Ust-Ishim [38]) align, suggesting that these diverse groups all trace their ancestry back to a common ancestral group, which likely represents the early migrants out of Africa.

### Adaptation to high-latitude environments

**Fig 4:**
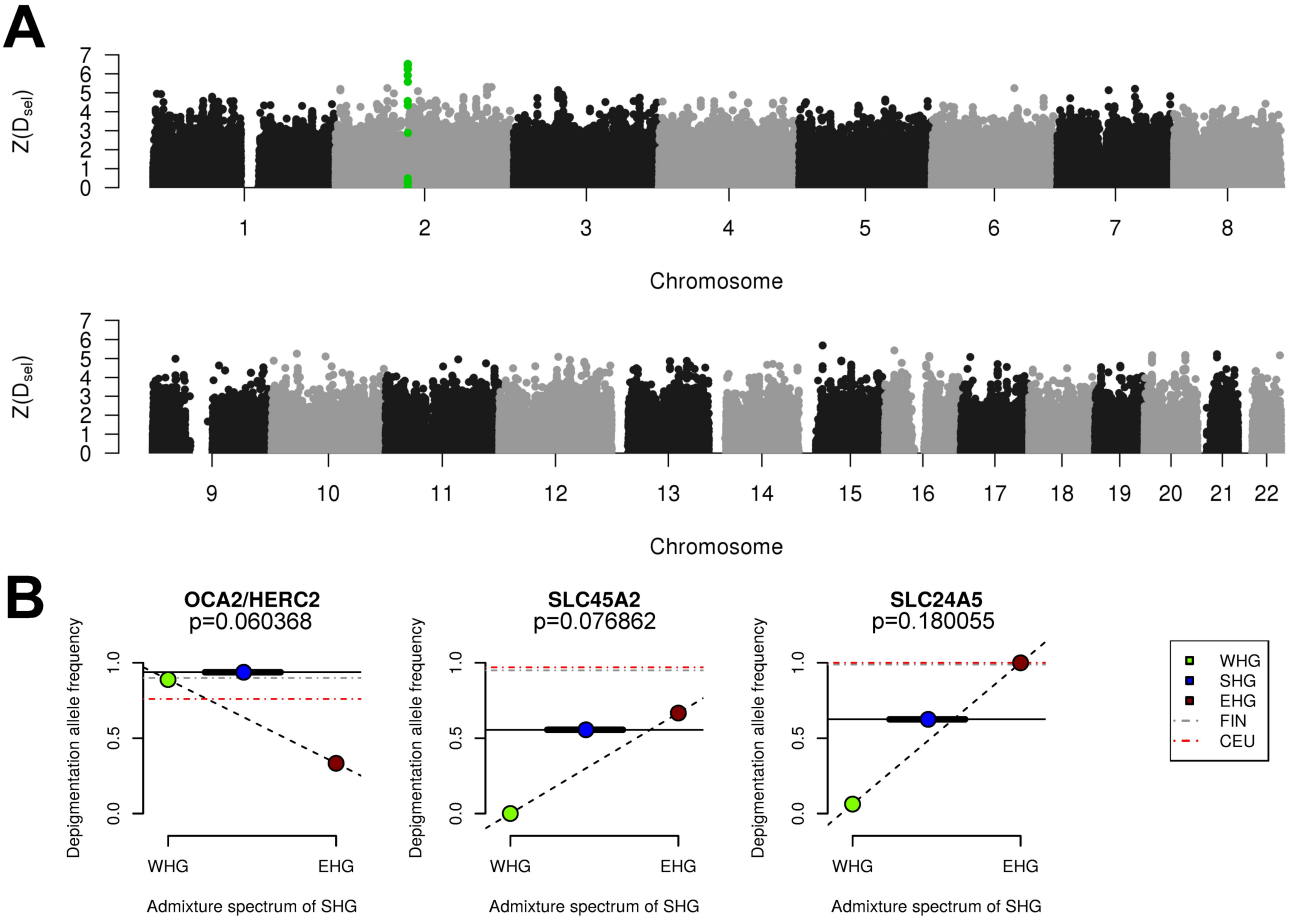
Adaptation to high-latitude environments. (A) Plot of similarity between Mesolithic allele frequency and FIN allele frequency in contrast to difference to TSI allele frequency using the statistic D_sel_. The figure shows all positive Z scores representing the number of standard deviations each SNP deviates from the mean. The green-highlighted SNPs are all located in the *TMEM131* gene. The plot was made with qqman [49]. (B) Derived allele frequencies for three pigmentation-associated SNPs (*SLC24A5*, *SLC45A2*, which are associated with skin pigmentation, and *OCA2/HERC2*, which is associated with eye pigmentation). The dashed line connecting EHG and WHG represents potential allele frequencies if SHG were a linear combination of admixture between EHG and WHG. The solid horizontal line represents the derived allele frequency in SHG. The blue symbols representing SHGs were set on the average genome-wide WHG and EHG mixture proportion (on x-axis) across all SHGs, and the thick black line represents the minimum and maximum admixture proportions across all SHGs. Dashed horizontal lines represent modern European populations (CEU). The *p*-values were estimated from simulations of SHG allele frequencies based on their genome-wide ancestry proportions (S9 Text). Data shown in this figure can be found in S1 Data. CEU, Utah residents with Central European ancestry; EHG, eastern hunter-gatherer; FIN, modern-day Finnish individual; SHG, Scandinavian hunter-gatherer; TSI, modern-day Tuscan individual; WHG, western hunter-gatherer.

With the aim of detecting signs of adaptation to high-latitude environments and selection during and after the Mesolithic period, we employed two different approaches that utilize the Mesolithic genomic data. In the first approach, we assumed that SHGs adapted to high-latitude environments of low temperatures and seasonally low levels of light, and searched for gene variants that carried over to modern-day people in northern Europe. Modern-day northern Europeans trace limited amounts of genetic material back to the SHGs (due to the many additional migrations during later periods), and any genomic region that displays extraordinary genetic continuity would be a strong candidate for adaptation in people living in northern Europe across time. We designed a statistic, D_sel_ (S9 Text), that captures this specific signal and scanned the whole genome for gene variants that show strong continuity (little differentiation) between SHGs and modern-day northern Europeans while exhibiting large differentiation to modern-day southern European populations [46] (Fig 4A; S9 Text). Six of the top 10 SNPs with greatest D_sel_ values were located in the *TMEM131* gene that has been found to be associated with physical performance [47], which could make it part of the physiological adaptation to cold [48]. This genomic region was more than 200 kbp (kilo base pairs) long and showed the strongest haplotypic differentiation between modern-day Tuscan individuals (TSIs) and modern-day Finnish individuals (FINs) across the genome (S9 Text). The particular haplotype was relatively common in SHGs, it is even more common among today’s Finnish population (S9 Text) and showed a strong signal of local adaptation (S9 Text). Other top hits included genes associated with a wide range of metabolic, cardiovascular, and developmental and psychological traits (S9 Text) potentially linked to physiological adaptation to cold environments [48].

In addition to performing this genome-wide scan, we studied the allele frequencies in three pigmentation genes (*SLC24A5*, *SLC45A2*, which have a strong effect on skin pigmentation, and *OCA2/HERC2*, which has a strong effect on eye pigmentation) in which the derived alleles are virtually fixed in northern Europeans today. The differences in allele frequencies of those three loci are among the highest between human populations, suggesting that selection was driving the differences in eye color, skin, and hair pigmentation as part of the adaptation to different environments [50–53]. All of the depigmentation variants at these three genes are in high frequency in SHGs in contrast to both WHGs and EHGs (Fig 4B). We conduct neutral simulations of the allele frequencies in an admixed SHG population to estimate *p*-values for observing these allele frequencies without selection (S9 Text). The *p*-values for all three SNPs are lower than 0.2; the combined *p*-value [54] for all three pigmentation SNPs is 0.028. Therefore, the unique configuration of the SHGs is not fully explained by the fact that SHGs are a mixture of EHGs and WHGs, but could rather be explained by a continued increase of the allele frequencies after the admixture event, likely caused by adaptation to high-latitude environments [50,52].

### Conclusion

By combining information from climate modeling, archaeology, and Mesolithic human genomes, we were able to reveal the complexity of the early migration patterns into Scandinavia and human adaptation to high-latitude environments. We disentangled two migration routes and linked them to particular archaeological patterns. We also demonstrated greater genetic diversity in Mesolithic northern Europe compared to southern and central Europe—in contrast to modern-day patterns—and showed that many genetic variants that were common during the Mesolithic period have been lost today. These findings reiterate the importance of human migration for dispersal of novel technology in human prehistory [14–20,27,40,55–58].

## Materials and methods

### Sample preparation

Genomic sequence data were generated from teeth and bone samples belonging to seven (eight, including SF13) Mesolithic SHGs (S1 Text). A detailed description on the archaeological background of the samples as well as post-LGM Scandinavia can be found in S1 Text. Additional libraries were sequenced for two previously published Neolithic hunter-gatherers, Ajvide58 and Ajvide70 [17] (S2 Text). All samples were prepared in the dedicated aDNA facilities at Uppsala University (SF9, SF11, SF12, SF13, SBj, Hum1, Hum2, Ajvide58, Ajvide70) and at Stockholm University (Steigen).

### DNA extraction and library building

Bones and teeth were decontaminated prior to analysis by wiping them with a 1% Sodiumhypoclorite solution and DNA-free water. Furthermore, all surfaces were UV irradiated (6 J/cm^2^ at 254 nm). After removing 1 millimeter of the surface, approximately 30–300 mg of bone was powderized and DNA was extracted following silica-based methods as in [59] with modifications as in [57,60] or as in [61] and eluted in 25–110 μl of EB buffer. Between one and 16 extractions were made from each sample and one extraction blank with water instead of bone powder was included per six to 10 extracts. Blanks were carried along the whole process until quantitative PCR (qPCR) and/or PCR and subsequent quantification.

DNA libraries were prepared using 20 μl of extract, with blunt-end ligation coupled with P5 and P7 adapters and indexes as described in [57,62]. From each extract one to five double stranded libraries were built. Because aDNA is already fragmented, the shearing step was omitted from the protocol. Library blank controls, including water as well as extraction blanks, were carried along during every step of library preparation. In order to determine the optimal number of PCR cycles for library amplification, qPCR was performed. Each reaction was prepared in a total volume of 25 μl, containing 1 μl of DNA library, 1X MaximaSYBRGreen mastermix, and 200 nM each of IS7 and IS8 [62] reactions were set up in duplicates. Each blunt-end library was amplified in four to 12 replicates with one negative PCR control per index-PCR. The amplification reactions had a total volume of 25 μl, with 3 μl DNA library, and the following in final concentrations: 1 X AmpliTaq Gold Buffer, 2.5 mM MgCl_2_, 250 μM of each dNTP, 2.5 U AmpliTaq Gold (Thermo Fisher Scientific, Waltham, MA), and 200 nM each of the IS4 primer and index primer [62]. PCR was done with the following conditions: an activation step at 94°C for 10 min followed by 10–16 cycles of 94°C for 30 s, 60°C for 30 s, and 72°C for 30 s, and a final elongation step of 72°C for 10 min. For each library, four amplifications with the same indexing primer were pooled and purified with AMPure XP beads (Agencourt; Beckman Coulter, Brea, CA). The quality and quantity of libraries was checked using Tapestation or BioAnalyzer using the High Sensitivity Kit (Agilent Technologies, Cary, NC). None of the blanks showed any presence of DNA comparable to that of a sample and were therefore not further analyzed. For initial screening, 10–20 libraries were pooled at equimolar concentrations for sequencing on an Illumina HiSeq 2500 using v.4 chemistry, and 125 bp paired-end reads or HiSeqX, 150 bp paired-end length using v2.5 chemistry at the SNP & SEQ Technology Platforms at Uppsala University and Stockholm University. After evaluation of factors such as clonality, proportion of human DNA, and genomic coverage samples were selected for resequencing, aiming to yield as high coverage as possible for each library.

### Generation of a high-coverage UDG-treated genome

Based on the results of the non-damage-repair sequencing, the SF12 individual was selected for large-scale sequencing in order to generate a high-coverage genome of high quality where damages had been repaired using UDG. In addition to the 15 extracts previously prepared and used for non-damage repair libraries, another 111 extracts were made based on a variety of silica-based methods [27,57,59,60]. From these 126 extracts, a total of 258 damage-repaired double-stranded libraries were built for Illumina sequencing platforms. Libraries were built as above, except a DNA repair step in which UDG and endonuclease VIII or USER enzyme (NEB) treatment was included in order to remove deaminated cytosines [63]. qPCR was performed in order to quantify the number of molecules and the optimal number of PCR cycles prior to amplification for each DNA library. Furthermore, this step included extraction blanks, library blanks, and amplification blanks to monitor potential contamination. All of these negative controls showed an optimal cycle of amplification significantly higher to those of our aDNA libraries (>10 cycles) and they were thus deemed as negative. Our experimental results show minimal levels of contamination, which is in concordance with mt DNA and X chromosome estimates of contamination (see S4 Text and Table 1). Each reaction was done in a total volume of 25 μl, containing 1 μl of DNA library, 1 X MaximaSYBRGreen mastermix (Thermo Fisher Scientific), and 200 nM each of IS7 and IS8 [62], reactions were set up in duplicate. The PCRs were set up using a similar system as for the nondamage repair samples (in quadruplicates that were pooled prior to cleanup of the PCR products), except for using AccuPrime DNA polymerase (Thermo Fisher Scientific) instead of AmpliTaqGold (Thermo Fisher Scientific) and the following PCR conditions: an activation step at 95°C for 2 min followed by 10–16 cycles of 95°C for 15 s, 60°C for 30 s, and 68°C for 1 min, and a final elongation step of 68°C for 5 min. Blank controls, including water as well as extraction blanks, were carried out during every step of library preparation. Amplified libraries were pooled, cleaned, quantified, and sequenced in the same manner as non-damage repaired libraries. A small proportion of the libraries (*n* = 14) were also subjected to whole genome capture (WGC) using European MYbaits from MYcroarray, and following the manufacturers protocol as done in [64].

In order to sequence libraries to depletion, two to eight libraries were pooled together and sequenced until reaching a clonality of >50%; if sequencing was halted before reaching that clonality level, it was either because the library was classified as unproductive based on the genome coverage generated, or that the sequencing goal (>55 × coverage) was already reached and further sequencing was deemed unnecessary. Sequencing was performed as above.

### Bioinformatic data processing and authentication

Paired-end reads were merged using MergeReadsFastQ_cc.py [65]; if an overlap of at least 11 base pairs was found, the base qualities were added together and any remaining adapters were trimmed. Merged reads were then mapped single-ended with bwa aln 0.7.13 [66] to the human reference genome (build 36 and 37) using the following nondefault parameters: seeds disabled -l 16500 -n 0.01 -o 2 [17,18]. To remove PCR duplicates, reads with identical start and end positions were collapsed using a modified version, to ensure random choice of bases, of FilterUniqSAMCons_cc.py [65]. Reads with less than 10% mismatches to the human reference genome, reads longer than 35 base pairs, and reads with mapping quality higher than 30 were used to estimate contamination.

The genetic data obtained from the two bone elements SF9 and SF13 showed extremely high similarities, which suggested that the two individuals were related. Using READ [67], a tool to estimate kin-relationship from aDNA, SF9 and SF13 were classified as either identical twins or the same individual. Therefore, we merged the genetic data for both individuals and refer to the merged individual as SF9 throughout the genetic analysis.

All data show damage patterns indicative of authentic aDNA (S3 Text). Contamination was estimated using three different sources of data: (1) the mt genome [68], (2) the X chromosome if the individual was male [69,70], and (3) the autosomes [71]. The data mapping to the human genome can be considered largely endogenous, as the contamination estimates were low across all three methods (S4 Text).

### Analysis of demographic history

Most population genomic analyses require a set of reference data for comparison. We compiled three different data sets from the literature and merged them with the data from ancient individuals (S6 Text). The three reference SNP panels were as follows:

- The Human Origins genotype data set of 594,924 SNPs genotyped in 2,404 modern individuals from 203 populations [18,39].
- A panel of 1,055,209 autosomal SNPs, which were captured in a set of ancient individuals by Mathieson et al. [20].
- To reduce the potential effect of ascertainment bias on SNP array data and of cytosine deamination on transition SNPs, we also ascertained 1,797,398 transversion SNPs with a minor allele frequency of at least 10% (to avoid the effect of Eurasian admixture into Yorubans) in Yorubans of the 1000 Genomes Project [32]. Those SNPs were extracted using vcftools [72].

These data sets were merged with ancient individuals of less than 15× genome coverage using the following approach: for each SNP site, a random read covering that site with minimum mapping quality 30 was drawn (using samtools 0.1.19 mpileup [73]) and its allele was assumed to be homozygous in the ancient individual. Transition sites were coded as missing data for individuals that were not UDG-treated, and SNPs showing additional alleles or indels in the ancient individuals were excluded from the data.

The six high-coverage ancient individuals (SF12, NE1 [27], Kotias [35], Loschbour [18], Stuttgart [18], and Ust-Ishim [38]) used in this study were treated differently, as we generated diploid genotype calls for them. First, the base qualities of all Ts in the first five base pairs of each read as well as all As in the last five base pairs were set to 2. We then used Picard [74] to add read groups to the files. Indel realignment was conducted with GATK 3.5.0 [66] using indels identified in phase 1 of the 1000 Genomes Project as reference [32]. Finally, GATK’s UnifiedGenotyper was used to call diploid genotypes with the parameters -stand_call_conf 50.0, -stand_emit_conf 50.0, -mbq 30, -contamination 0.02, and—output_mode EMIT_ALL_SITES using dbSNP version 142 as known SNPs. SNP sites from the reference data sets were extracted from the VCF files using vcftools [72] if they were not marked as low-quality calls. Plink 1.9 [75,76] was used to merge the different data sets.

We performed PCA to characterize the genetic affinities of the ancient Scandinavian genomes to previously published ancient and modern genetic data. PCA was conducted on 42 present-day west Eurasian populations from the Human Origins data set [18,39] using *smartpca* [77] with numoutlieriter: 0 and lsqproject: YES options. A total of 59 ancient genomes (52 previously published and 7 reported here) (S6 Text) were projected into the reference PCA space and computed from the genotypes of modern individuals. For all individuals, a single allele was selected randomly—making the data set fully homozygous. The result was plotted using the *ploteig* program of EIGENSOFT [77] with the–x and–k options.

### D and f statistics

*popstats* [78] was used to calculate D statistics to test deviations from a tree-like population topology of the shape ((*A*,*B*);(*X*,*Y*)) [39]. Standard errors were calculated using a weighted block jackknife of 0.5 Mbp. The tree topologies are balanced at zero, indicating no recent interactions between the test populations. Significant deviations from zero indicate a deviation from the proposed tree topology depending on the value. Positive values indicate an excess of shared alleles between A and X or B and Y, whereas negative values indicate more shared alleles between B and X or A and Y. Using an outgroup as population A limits the test results to depend on the recent relationships between B and Y (if positive) or B and X (if negative). Here, we used high-coverage Mota [37], Yoruba [32], and Chimp genome as (A) outgroups. *popstats* [78] was used to calculate f_4_ statistics in order to estimate shared drift between groups. Standard errors and Z scores for f_4_ statistics were estimated using a weighted block jackknife (Fig 1C).

### Model-based clustering

A model-based clustering algorithm, implemented in the *ADMIXTURE* software [79], was used to estimate ancestry components and to cluster individuals. *ADMIXTURE* was conducted on the Human Origins data set [18,39], which was merged with the ancient individuals as described above. Data was pseudo-haploidized by randomly selecting one allele at each heterozygous site of present-day individuals. Finally, the data set was filtered for LD using PLINK [75,76] with parameters (--indep-pairwise 200 25 0.4), this retained 289,504 SNPs. *ADMIXTURE* was run in 50 replicates with different random seeds for ancestral clusters from K = 2 to K = 20. Common signals between independent runs for each K were identified using the LargeKGreedy algorithm of *CLUMPP* [80]. Clustering was visualized using rworldmap, ggplot2, SDMTools, and RColorBrewer packages of GNU R version 3.3.0. Starting from K = 3, when the modern samples split up into an African and eastern and western Eurasian clusters, the Mesolithic Scandinavians from Norway show slightly higher proportions of the Eastern cluster than Swedish Mesolithic individuals. This pattern continues to develop across higher values of K and it is consistent with the higher Eastern affinities of the Norwegian samples seen in the PCA and *D-* and f4 statistics. The results for all Ks are shown in S1 Fig.

In addition to *ADMIXTURE*, we assessed the admixture patterns in Mesolithic Scandinavians using a set of methods implemented in *ADMIXTOOLS* [39], *qpWave* [81], and *qpAdm* [19]. Both methods are based on f_4_ statistics, which relate a set of test populations to a set of outgroups in different distances from the potential source populations. We used the following set of outgroup populations from the Human Origins data set: Ami_Coriell, Biaka, Bougainville, Chukchi, Eskimo_Naukan, Han, Karitiana, Kharia, and Onge. We first used *qpWave* to test the number of source populations for Mesolithic west Eurasians (WHG). *qpWave* calculates a set of statistics X(u,v) = f_4_(u0, u; v0, v) where u0 and v0 are populations from the sets of test populations L and outgroups R, respectively. To avoid having more test populations than outgroups, we built four groups consisting of (1) genetically western and central hunter-gatherers (Bichon, Loschbour, KO1, LaBrana), (2) EHGs (UzOO74/I0061, SVP44/I0124, UzOO40/I0211), (3) Norwegian hunter-gathers (Hum1, Hum2, Steigen), and (4) Swedish hunter-gatherers (individuals from Motala and Mesolithic Gotland). qpWave tests the rank of the matrix of all X(u,v) statistics. If the matrix has rank m, the test populations can be assumed to be related to at least m + 1 “waves” of ancestry, which are differently related to the outgroups. A rank of 0 is rejected in our case (*p* = 3.13e-81), whereas a rank of 1 is consistent with the data (*p* = 0.699). Haak et al. [19] already showed, using the same approach, that WHG and EHG descend from at least two sources (confirmed with our data as rank 0 is rejected with *p* = 1.66e-86, whereas rank 1 is consistent with the data), and adding individuals from Motala does not change these observations. Therefore, we conclude that European Mesolithic populations, including Swedish and Norwegian Mesolithic individuals, have at least two source populations.

We then used *qpAdm* to model Mesolithic Scandinavian individuals as a 2-way admixture of WHG and EHG. *qpAdm* was run separately for each Scandinavian individual x, setting T = x as target and S = (EHG, WHG) as sources. The general approach of *qpAdm* is related to *qpWave*: target and source are used as L (with T being the base population), and f_4_ statistics with outgroups from R (same as above) are calculated. The rank of the resulting matrix is then set to the number of sources minus one, which allows to estimate the admixture contributions from each population in S to T. The results are shown in Fig 1. We calculate a Z score for the difference between Norwegian and Swedish SHG as $Z=\frac{a_{nor}-a_{swe}}{\sqrt{{SE}_{nor}^{2}+{SE}_{swe}^{2}}}$ where *a* are the ancestry estimates and *SE* are the respective block-jackknife estimates of the standard errors.

### RoH

Heterozygosity is a measurement for general population diversity and its effective population size. Analyzing the extent of homozygous segments across the genome can also give us a temporal perspective on the effective population sizes. Many short segments of homozygous SNPs can be connected to historically small population sizes, whereas an excess of long RoH suggests recent inbreeding. We restricted this analysis to the six high-coverage individuals (SF12, NE1, Kotias, Loschbour, Stuttgart, Ust-Ishim) for which we obtained diploid genotype calls and we compared them to modern individuals from the 1000 Genomes Project. The length and number of RoH were estimated using Plink 1.9 [75,76] and the parameters--homozyg-density 50,--homozyg-gap 100,--homozyg-kb 500,--homozyg-snp 100,--homozyg-window-het 1,--homozyg-window-snp 100,--homozyg-window-threshold 0.05, and--homozyg-window-missing 20. The results are shown in Fig 3A.

### LD

Similar to RoH, the decay of LD harbors information on the demographic history of a population. Long-distance LD can be caused by a low effective population size and past bottlenecks. Calculating LD for aDNA data is challenging, as the low amounts of authentic DNA usually just yields haploid allele calls with unknown phase. In order to estimate LD decay for ancient populations, we first combine two haploid ancient individuals to a pseudo-diploid individual (similar to the approach chosen for conditional nucleotide diversity, S7 Text). Next, we bin SNP pairs by distance (bin size 5 kb) and then calculated the covariance of derived allele frequencies (0, 0.5, or 1.0) for each bin. This way, we do not need phase information to calculate LD decay because we do not consider multilocus haplotypes, which is similar to the approach taken by ROLLOFF [39,82] and ALDER [83] to date admixture events based on admixture LD decay. For Fig 3B, we used two modern 1000 Genomes Project populations to scale the LD per bin. The LD between two randomly chosen PELs (modern-day Peruvian individuals) was set to 1 and the LD between two randomly chosen TSIs was set to 0. This approach is used to obtain a relative scale for the ancient populations, and we caution against a direct interpretation of the differences to modern populations because technical differences in the modern data (e.g., SNP calling or imputation) may have substantial effects.

### Effective population size

We are using MSMC’s implementation of PSMC’ [44] to infer effective population sizes over time from single high-coverage genomes. We restrict this analysis to UDG-treated individuals (SF12, Loschbour, Stuttgart, Ust-Ishim) as postmortem damage would cause an excess of false heterozygous transition sites. Input files were prepared using scripts provided with the release of MSMC (https://github.com/stschiff/msmc-tools) and MSMC was run with the nondefault parameters--fixedRecombination and -r 0.88 in order to set the ratio of recombination to mutation rate to a realistic level for humans. We also estimate effective population size for six high-coverage modern genomes [84] (Fig 3C). We plot the effective population size assuming a mutation rate of 1.25x10e-8 and a generation time of 30 years. The curves for ancient individuals were shifted based on their average C14 date. Additionally, we used multihetsep_bootstrap.py to generate 100 bootstraps per individual. The results are shown in S4 Fig.

### Detecting adaptation to high-latitude environments

We scanned the genomes for SNPs with similar allele frequencies in Mesolithic and modern-day northern Europeans and contrasted it to a modern-day population from southern latitudes. Pooling all Mesolithic Scandinavians together, we obtain an allele frequency estimate for SHGs, which is compared to FINs and TSIs from the 1000 Genomes Project [32]. We use the Finnish population as representatives of modern-day northern Europeans (this sample contains the largest number of sequenced genomes from a northern European population). Tuscans are used as an alternative population, who also trace some ancestry to Mesolithic populations, but who do not trace their ancestry to groups that lived at northern latitudes in the last 7,000–9,000 years. Our approach is similar to PBS [85] and inspired by DAnc [46]. For each SNP, we calculated the statistic D_sel_, comparing the allele frequencies between one ancestral and two modern populations:

$${\text{D}}_{\text{sel}}\;=\;\left|{\text{DAF}}_{\text{SHG}}\;-\;{\text{DAF}}_{\text{TSI}}\right|\;-\;\left|{\text{DAF}}_{\text{SHG}}\;-\;{\text{DAF}}_{\text{FIN}}\right|$$

This scan was performed on all transversion SNPs extracted from the 1000 Genomes Project data. Only sites with a high-confidence ancestral allele in the human ancestor (as used by the 1000 Genomes Project [32]) and with coverage for at least six ancient Scandinavians were included in the computation. More information can be found in S9 Text.

## Supporting information

S1 Table Allelic states at phenotypically relevant SNPs (see also S8 Text). (XLSX)

S1 Fig Complete results of unsupervised ADMIXTURE K = 2 to K = 20. (PDF)

S2 Fig Distributions of all individual D statistics. Positive D statistics for WHGs are all involving the low quality and slightly contaminated SF11 as Swedish SHGs. Data shown in this figure can be found in S1 Data. SHG, Scandinavian hunter-gatherer; WHG, western hunter-gatherer.
(PDF)

S3 Fig f_4_ statistics (see also Fig 1C) plotted against chronological age and longitude of the samples. Pink symbols indicate Latvian samples. Data shown in this figure can be found in S1 Data.
(PDF)

S4 Fig MSMC results with 100 bootstraps. Data shown in this figure can be found in S1 Data. MSMC, multiple sequentially Markovian coalescent.
(PDF)

S1 Text Archaeological background. (PDF)

S2 Text DNA sample preparation. (PDF)

S3 Text Processing of NGS data. NGS, next-generation sequencing.
(PDF)

S4 Text Estimates of contamination. (PDF)

S5 Text Uniparental markers. (PDF)

S6 Text Basic population genomic analysis. (PDF)

S7 Text Diversity estimates. (PDF)

S8 Text Functional variation in ancient samples. (PDF)

S9 Text Adaptation to high-latitude climates. (PDF)

S1 Data Data shown in figures. (XLSX)

## Abbreviations

aDNA: ancient DNA
BP: before present
cal: calibrated
CEU: Utah residents with Central European ancestry
CHG: Caucasus hunter-gatherer
Chimp: Chimpanzee
EHG: eastern hunter-gatherer
FIN: modern-day Finnish individual
kbp: kilo base pairs
LD: linkage disequilibrium
LGM: Last Glacial Maximum
MSMC: multiple sequentially Markovian coalescent
mt: mitochondrial
PCA: principal component analysis
PEL: modern-day Peruvian individual
qPCR: quantitative PCR
RoH: runs of homozygosity
SHG: Scandinavian hunter-gatherer
TSI: modern-day Tuscan individual
UDG: Uracil-DNA-glycosylase
WGC: whole genome capture
WHG: western hunter-gatherer
