# Supplementary figures and images for: Population genomics of Mesolithic Scandinavia: Investigating early postglacial migration routes and high-latitude adaptation
Source: PLoS Biol. 2018 Jan 9;16(1):e2003703. doi: 10.1371/journal.pbio.2003703 (PMC5760011; doi:10.1371/journal.pbio.2003703)

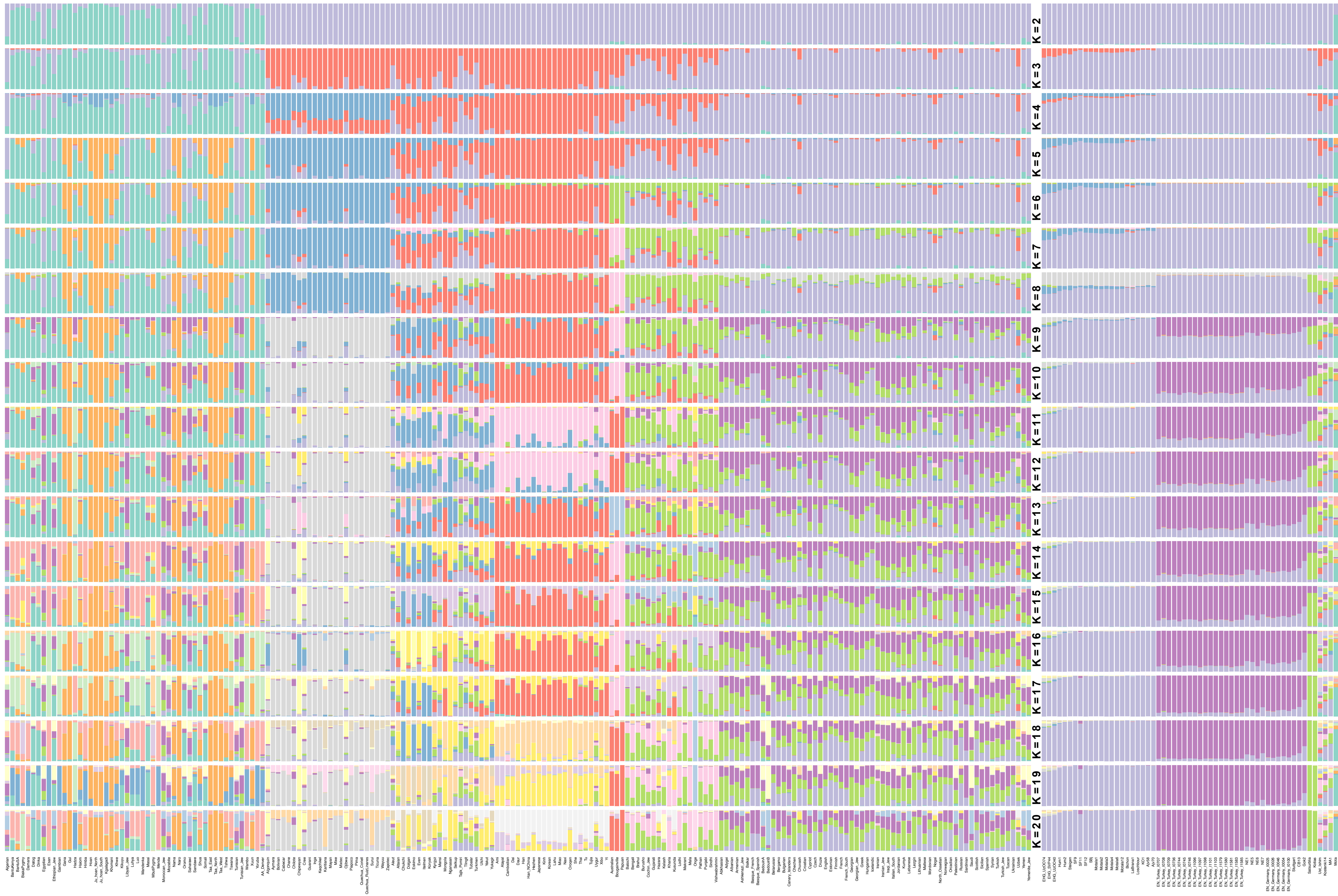

Supplement: S1 Fig — (PDF) [file pbio.2003703.s002.pdf]

D(Chimp, X; SHG\_swe, SHG\_nor)

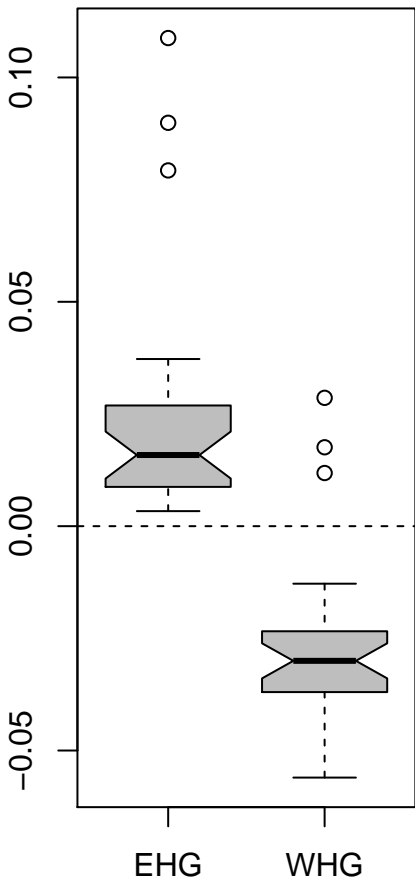

Supplement: S2 Fig — Positive D statistics for WHGs are all involving the low quality and slightly contaminated SF11 as Swedish SHGs. Data shown in this figure can be found in S1 Data. SHG, Scandinavian hunter-gatherer; WHG, western hunter-gatherer. (PDF) [file pbio.2003703.s003.pdf]

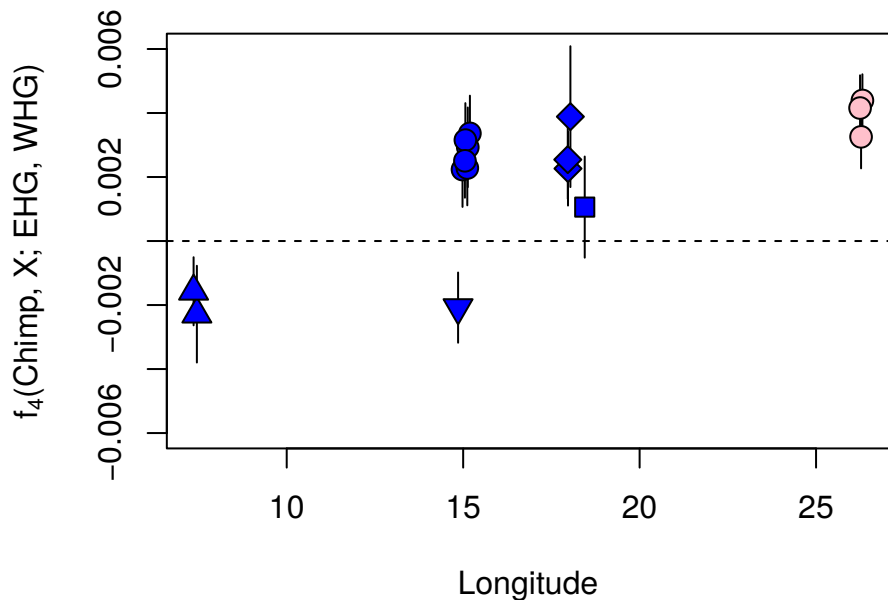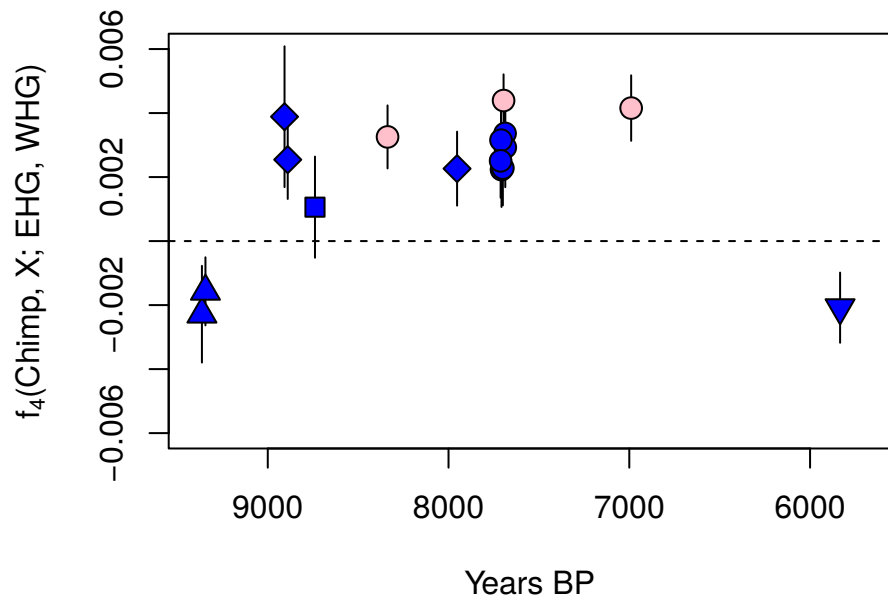

Supplement: S3 Fig — Pink symbols indicate Latvian samples. Data shown in this figure can be found in S1 Data. (PDF) [file pbio.2003703.s004.pdf]

**French**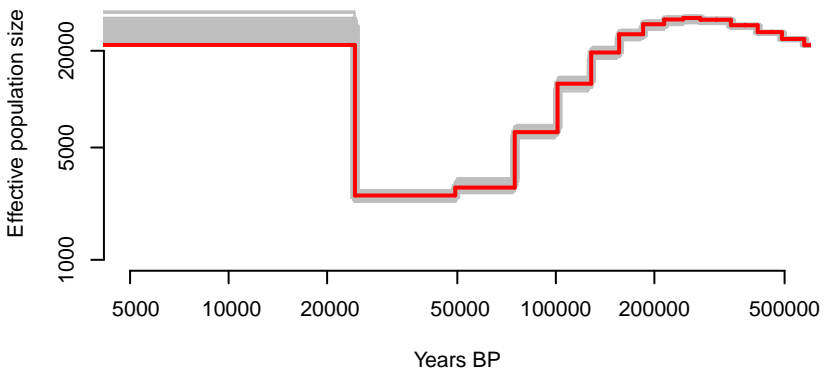**Sardinian**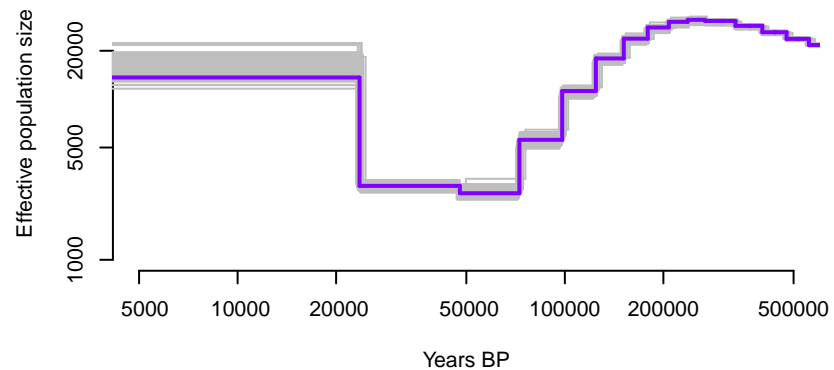**Karitiana**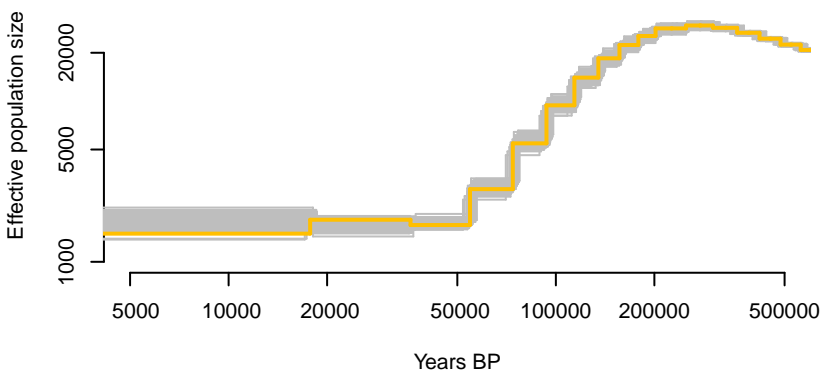**Yoruba**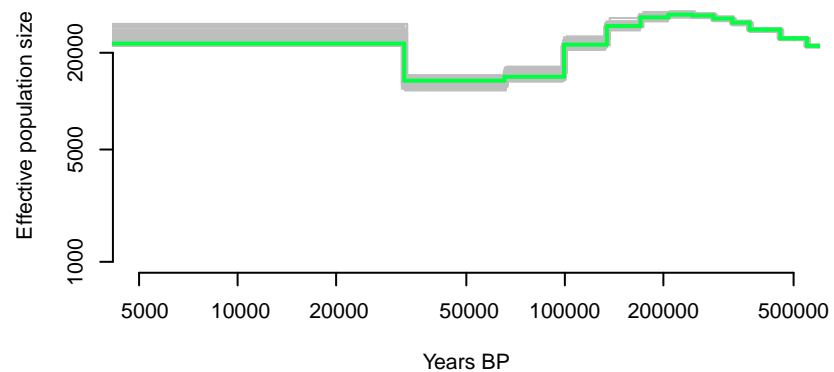**Stuttgart**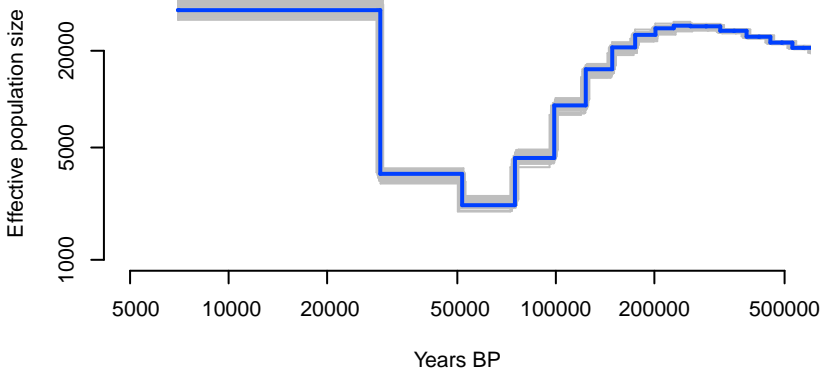**Ust-Ishim**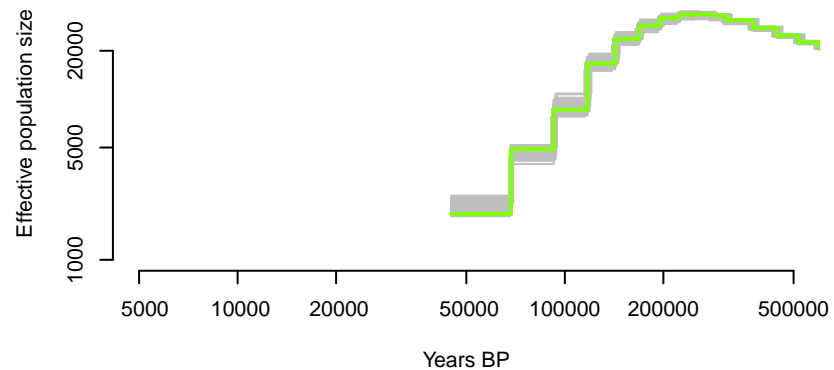**Loschbour**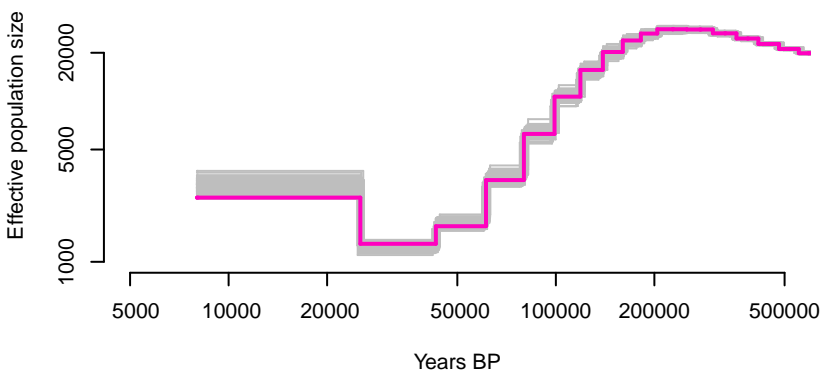**SF12**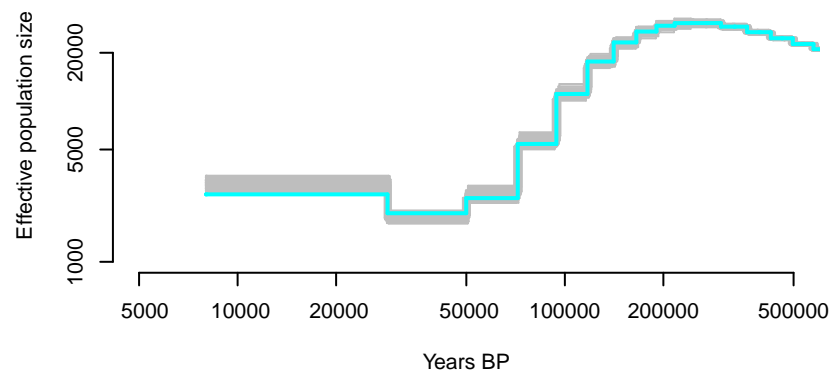

Supplement: S4 Fig — Data shown in this figure can be found in S1 Data. MSMC, multiple sequentially Markovian coalescent. (PDF) [file pbio.2003703.s005.pdf]
