# Supplementary material for: Population genomics of Mesolithic Scandinavia: Investigating early postglacial migration routes and high-latitude adaptation
Source: PLoS Biol. 2018 Jan 9;16(1):e2003703. doi: 10.1371/journal.pbio.2003703 (PMC5760011; doi:10.1371/journal.pbio.2003703)
Supplement: S1 Text — (PDF) [file pbio.2003703.s006.pdf]

S1 Archaeological background

S1.1 Introduction

The late-glacial and early post-glacial dispersal of humans to Scandinavia has been the focus of extensive archaeological debate. The complex history of climatic fluctuations during the Eurasian deglaciation (Figure S1.1) created a variety of habitats for the first humans in the region [1–7]. How the dispersal took place varied over time and in the beginning it followed a wave and advance model with expansions and contractions, linked both to changes in climate and to the geological development of Scandinavia and the Baltic Sea [8–14]. The fact that many Pleistocene coastal areas became submerged due to the post-glacial rise in sea level makes it particularly challenging to explore the earliest coastal activities. In Northern Scandinavia, however, where the Eurasian Ice sheet was at its thickest, isostatic land-uplift in many places more than compensated sea-level rise, thus permitting studies of early Holocene coastal settlement. The first human colonization of the region followed a wave of advance model with expansions and contractions linked to these climatic and landscape developments in Scandinavia and the Baltic Sea Area [8–14]. Our present knowledge of the various pulses of human colonization of the areas influenced by the Eurasian Ice Sheet rests on typological and technological analyses of lithic assemblages, information on subsistence and dietary pattern but also natural history proxy data. We here present novel genetic data of human remains dating between 9,500 and 6,000 cal BP that offer a unique possibility to examine the demographic history of the Early Holocene in northern Europe.

The earliest evidence for human presence in southern Scandinavia consists of lithic artifacts assigned to the Hamburgian culture. These date to c. 16,000 cal BP; thus roughly contemporaneous with the earliest radiocarbon dated finds of reindeers, the most important prey during this period. However, around 14,000 cal BP the evidence of human presence becomes scarce [10,11], only to be followed by a new advance of apparently episodic pioneer settlements from around 12,800 cal BP [15–18].

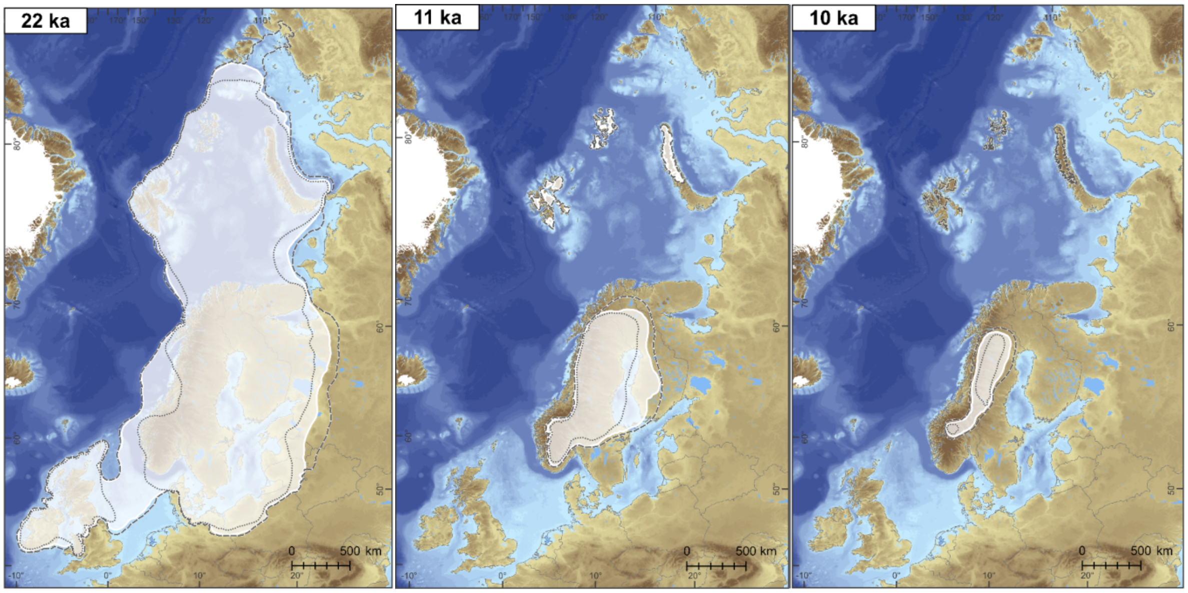

Figure S.1.1 Time-slice reconstruction of the extent of the Eurasian ice sheet, during the Last Glacial Maximum (LGM) and the relevant periods. Dates are expressed in calibrated years BP. From Hughes et al. [19], Figure 6. Published under CC-BY 4.0.

From around 11,700 cal BP the archaeological record indicates a more consistent northern expansion along the Norwegian coast, following the retreat of the ice sheet. By c. 11,500–11,300 cal BP there are approximately 800 settlements throughout the Norwegian coast-line connected to this pulse of migration, often linked to the Fosna and Komsa complexes in western and northern Norway [20–25]. Artefacts and tools associated with these pioneer settlements show links to mobile hunter-gatherer groups with a southern origin in the Ahrensburgian tradition [26]. The hunter-gatherers that had previously hunted reindeer, later on adopted a strictly marine economy. Seals and other marine mammals constituted an important pull factor for the terrestrial hunter-gatherers that became marine foragers in early Holocene Scandinavia [27–29]. The use of marine resources seems to have been the main mode of subsistence along the Norwegian coast, although terrestrial mammals like reindeer were also exploited during occasional mountain hunts [6,30–34]. Thus, the current hypothesis concerning the colonization of Scandinavia assumes that settlements rapidly advanced from the south to western Norway (Fosna) and finally to the Varanger area in the north (Komsa) [24–26,30,31,33,35–38]. Coastal areas of western Sweden and northern Norway were now free of ice and represented a partially protected archipelago rich in marine resources where boats were important [14,30,31,35,39].

The oldest settlements on the eastern side of the Baltic Sea, from southern Finland, are dated to around 11,100 cal BP [36–38], and for Northern Finland around 10,300 cal BP [37,38,40]. Further south, in the Baltic countries dates fall around 10,800-10,200 cal BP [41]. Archaeological finds indicate two pulses of migration into north eastern Fennoscandia [40]. The first pulse, however, did not reach far into northernmost Finland and Norway and was, followed by a decline, possibly because of a cold event around 10,300 cal BP. A stronger Late Preboreal migration reached northern Norway after around 10,150 cal BP. The material culture found at these sites, especially the lithic technology, labelled as Post-Swiderian [42], has its closest counterparts within the Late Glacial microlithic Swiderian complexes of northeastern Europe, found to the east of the Ahrensburgian complexes [16,43]. Further east, to the north of the Ural

Mountains in Russia, human settlements have been found on the northern coasts of the continent dating to the Late Paleolithic, possibly as early as around 43,000-40,000 cal BP [44]. Recent data on the deglaciation [19,45], and archaeological evidence [46], such as the Pymva Shor and other sites (dated to c. 26,300 - 11,600 cal BP) indicate that a north-eastern route for human migration into Scandinavia may also be considered. Several Early Mesolithic sites are known along the Kola Peninsula [47] and the White Sea coast, which are contemporaneous to the northern Scandinavian sites [35]. However, at present we do not have detailed knowledge of their chronology (Tikhonov, A. pers. comm.).

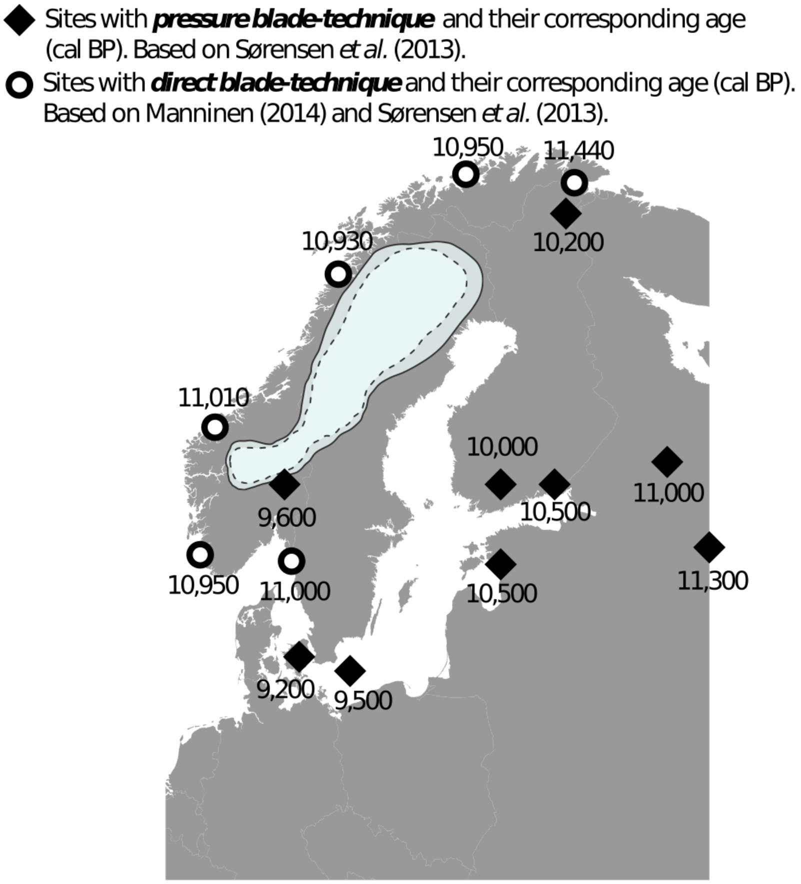

Figure S1.2 Distribution of 'direct blade' and 'pressure blade' lithic artifacts from radiocarbon dated archaeological sites. The map was produced using rworldmap [48]. Locations and dates are based on [5,49,50].

S1.2 Lithic technology in Mesolithic Northern Scandinavia

The pioneer settlements of central and northern Scandinavia c. 11,500-11,300 cal BP shared a flint industry characterized by a macro-flake technology and a direct knapping blade technique that can also be found in southern Scandinavia. Certain artefact types such as tanged points and single-edged points, have their closest counterparts in the Ahrensburgian tradition in south

Scandinavia and northern Europe [7,26,30,49–51]. This technology is widespread in western Sweden and Norway, the Hensbacka, Fosna and Komsa complexes, and evolved slowly for c. 1,200 years.

Around 10,300 cal BP archaeological finds of another lithic technology (pressure blade technique) appear in northern Finland and northernmost Norway, this time showing similarities with the post-Swiderian sites in north-western Russia dated to 11,300–7,700 cal BP [14,32,33,38,49–52]. This technology consists of slotted-bone tools with flint edges made of regular micro-blades that were pressed from conical blade cores. It has an eastern origin with its earliest appearance in northern East Asia between 30,000-20,000 cal BP [53–55] and is introduced to the Baltic area via the Upper Volga area in Russia [56]. From here, the technology spread, either by direct migration of people, or by knowledge transmission, via a northern or a southeastern route. Rankama & Kankaanpää [38] conclude that the closest technological influences on the north Finnish site at Sujala and the Norwegian site Fállegoahtesajeguolbba provide strong evidence for people of the post-Swiderian groups coming from north-western Russia and establishing themselves in northern Lapland.

The pressure blade technology developed in areas where flint was an important raw material, but was adapted to local raw materials in northern Scandinavia and Finland [14,32,33] and eventually in middle Sweden [52]. Raw materials such as quartz, quartzite, sandstone, slate and flint have variable and different fracture patterns and they offer different possibilities for tool production. In northern Norway, Sweden and all of Finland [52,57] other techniques were also adopted and in e.g. Finland a flake based technology was much more common than a blade technology [57].

It has been suggested that the pressure blade technology dispersed to southern Scandinavia from the north [14,32,33,38,49,52] (Figure S1.2). The technology is found in southern Norway as early as c. 10,300 cal BP [32,33,49,50,52,57] and in southern Scandinavia and western Europe around 9,000 cal BP to 7,800 cal BP [50,58]. It is interesting that the technology appears earlier in southern Norway than in both Denmark and south of the Baltic.

S1.3 Site description and analyzed individuals

We have studied the genetic variation in eight samples (representing 7 unique individuals) from four Mesolithic coastal sites: Hummervikholmen in Southern Norway, Steigen in Northern Norway, and Stora Förvar and Stora Bjers on islands off the Swedish coast in the Baltic proper. The samples and/or other skeletal elements from the same individuals have also been subjected to stable carbon and nitrogen isotope analysis and radiocarbon dating (Table 1). The individuals from Hummervikholmen, Stora Förvar and Stora Bjers all date between c. 9,500 and 8,600 cal BP, i.e. the Boreal chronozone in the Early Holocene, while the individual from Steigen dates considerably later, c. 6,000–5,800 cal BP, i.e. the Atlantic chronozone in the Middle Holocene. Previously published genetic, stable isotope and radiocarbon data from two Mesolithic sites in Motala on the Swedish mainland, spanning the period 8,200–6,800 cal BP (the Atlantic chronozone in the Middle Holocene) were used for comparison.

Table S.1.1 Overview of all samples analyzed sorted by site: individuals, elements, analysis performed, and lab codes used. Detailed data in Tables S1.1-2.

Site	Short name	Individual	Skeletal element	DNA	IRMS $\delta^{13}C$/ $\delta^{15}N$	^{14}C date	AMS $\delta^{13}C$	Lab codes used (DNA, IRMS, AMS)
Hummervikholmen, Norway	Hum1	Hummervikholmen Ind. 1	Cranial fragment		x	x	x	SOG105, TRa-954, TUa-1257
Hummervikholmen, Norway	Hum1	Hummervikholmen Ind. 1	Left 2nd maxillary incisor	x	x			H22, SOG106
Hummervikholmen, Norway	Hum		Occipital bone (cranium)		x	x	x	SOG103, TRa-952, TUa-2106
Hummervikholmen, Norway	Hum2	Hummervikholmen Ind. 2	Left 1st maxillary molar	x	x			H26, SOG107
Hummervikholmen, Norway	Hum		Frontal bone (cranium)		x	x	x	SOG101, TRa-951, TUa-2105
Hummervikholmen, Norway	Hum		Tibia		x	x	x	SOG104, TRa-953, TUa-2108
Hummervikholmen, Norway	Hum		Femur			x	x	TUa-2107
Steigen, Norway	Steigen	Steigen Ind. 1	Right 1st mandibular molar		x			STE 01
Steigen, Norway	Steigen	Steigen Ind. 1	Right 2nd mandibular molar	x	x			Stg001, STE 02
Steigen, Norway	Steigen	Steigen Ind. 1	Left 3rd mandibular molar		x			STE 03
Steigen, Norway	Steigen	Steigen Ind. 1	Mandible		x	x	x	STE 04, Beta-349961
Stora Förvar, Sweden	SF9		Parietal bone (cranium)	x	x	x		Sf9, Beta-399027
Stora Förvar, Sweden	SF11		Tibia	x	x	x	x	Sf11, Ua-45742, Beta-448532
Stora Förvar, Sweden	SF12		Femur	x	x	x	x	Sf12, Ua-45741, Beta-448531
Stora Förvar, Sweden	SF13		Os coxae	x	x	x	x	Sf13, Beta-386399, Beta-448533
Stora Bjers, Sweden	SBj	Stora Bjers Ind. 1	Right 1st, 2nd and 3rd mandibular molars, tibia	x		x	x	sbj001, Ua-46147

S1.3.1 Hummervikholmen, Norway

In 1994, human skeletal remains were found at Hummervikholmen, a small island in the Søgne archipelago in Vest-Agder County in southernmost Norway [34,59,60]. These remains turned out to be the oldest dated skeletal remains from Norway [34,59,60]. The submerged site was investigated by the Norwegian Maritime Museum (NMM) in 1994-95 [59]. Recent dredging had damaged c. 60% of the site, and a stone foundation had disturbed the original beach-zone. The

shallow sub-sea sediments had been partially removed down to the bedrock. During the sieving of the re-deposited sediments several human bone fragments were recovered. Only a large, almost complete skull, a tooth, and a thighbone appeared to be *in situ [59]*. The two samples included in the present study were found *in situ*: the cranium of Individual 1 (Hum1) and a tooth from another individual (Hum 2).

Five distinct stratigraphic layers were identified, described from the top down: (1) marine silty sand, (2) compact oyster bank, (3) clay deposit with decomposed organic material, (4) intermittently occurring organic layer with skeletal remains, covering, (5) sterile moraine and/or bedrock [59]. The dating of the layer sequence is consistent and suggests that the oyster bank was deposited during the Tapes maximum (Atlantic period) in the relatively warm, calm waters, thus preserving the skeletal remains by sealing them in the thin organic bottom layer.

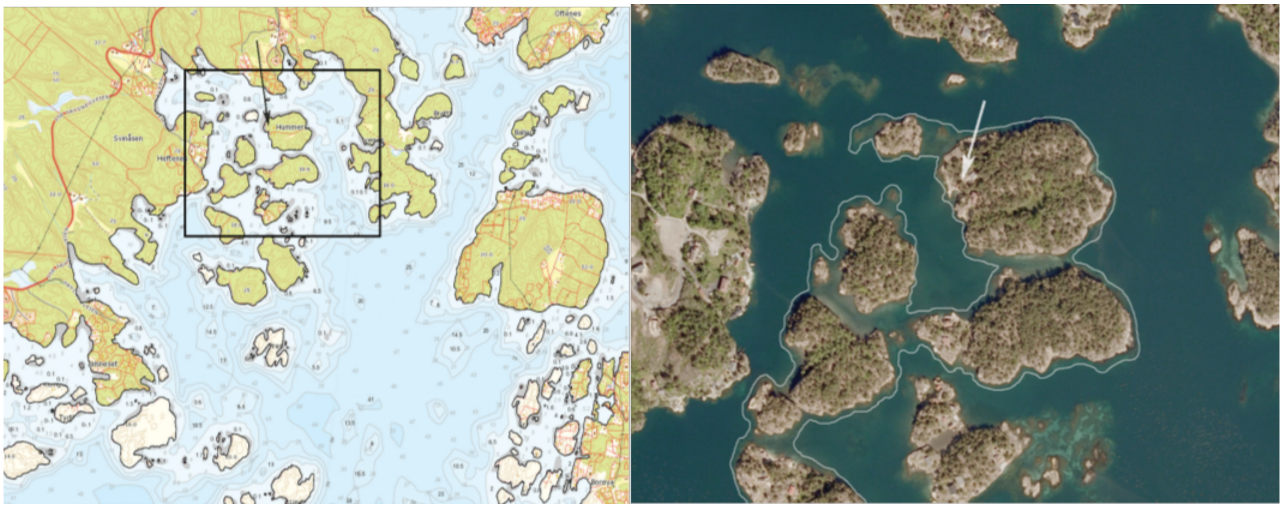

Figure S1.3 The Søgne archipelago indicating the location of Hummervikholmen (site marked with arrow) and the – 2 m a.s.l. contour line (marked in white). Drawing by Pål Nymoen. Source: www.kulturminnesok.no.

At least three to five individuals are represented in the material from Hummervikholmen, and the bone remains are well preserved, Figure S1.4. The assemblage consists of an almost complete skull (Individual1/Hum1), an occipital fragment (Individual2/Hum2), a frontal bone fragment (Individual 3), an almost intact left femur, and a damaged left tibia. The two postcranial bones are gracile; but it is not clear which of these adult bones belongs to which individual. The most complete skull (Individual1/Hum1) is probably a female, *c*. 35–40 years of age at death. The skull is robust, resembling other Scandinavian Mesolithic female skulls [59,61].

The findings indicate the presence of a burial tradition in Norway during the Middle Mesolithic. The remains represent unburnt burials, inhumations. The most complete skull was positioned with the back of the head downwards—this is the only potential indication of a dorsal position, but it could have been caused by marine erosion during the Tapes transgression. A more detailed knowledge of the original position of the bodies cannot be established. There are neither signs that the dead were provided with any grave goods nor of rituals, e.g. ochre or physical cutting. The Hummervikholmen grave site thus adds to a diverse image of Middle Mesolithic burial traditions in Scandinavia that is clearly distinguished from the later, larger and more elaborate

known Mesolithic grave sites from the Atlantic period like Skateholm and Vedbæk [62–64]. To date, Hummervikholmen is the most prominent submerged Mesolithic site in Norway.

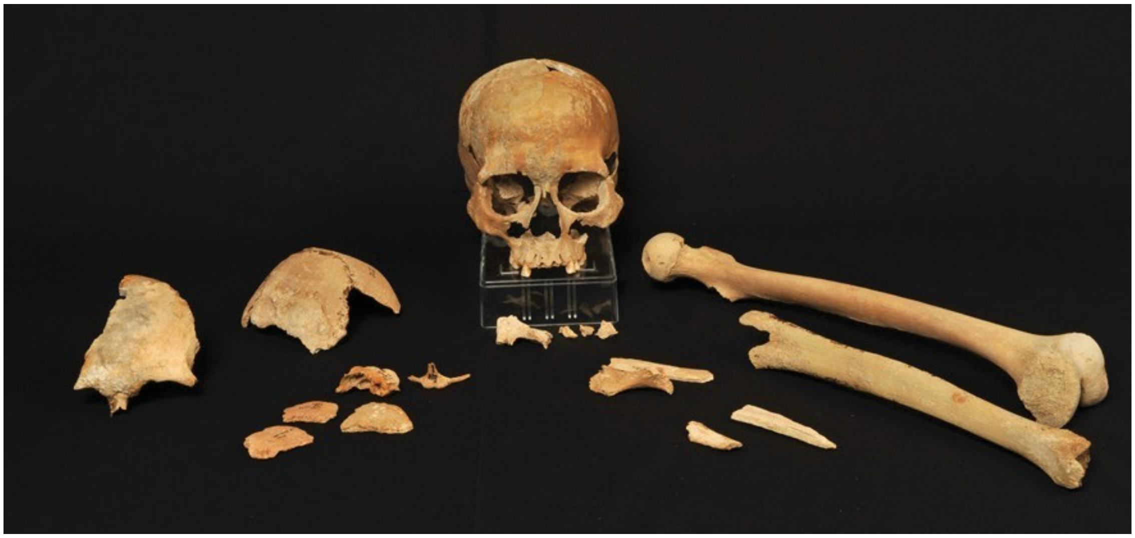

Figure S1.4 Skeletal fragments from the Hummervikholmen site (Photo: Beate Kjørslevik)

S1.3.2. Steigen, Norway

The Steigen human remains were discovered in a cave on a small island, Måløya, in Steigen municipality, Nordland County, Norway. The island is situated in a small archipelago some 5 km west of Sørskott on the Norwegian mainland (Figure S1.5). The cave, situated in a crevice running in a north-south direction, was discovered in 1996, but was not properly analyzed and documented until 2011, when a human mandible was found inside the cave and brought to Tromsø Museum (Figure S1.6). Today the opening of the cave is situated c. 30 m above present sea level, whereas the actual find place for the mandible is situated some 25 m above the present-day sea level, which coincides with the sea level at c. 8,500–5,500 cal BP. It is a natural cave with an inner height exceeding 20 m in some places. An investigation in 2013, with the purpose of measuring the cave and assessing the security and potential for an archaeological excavation, indicated there was a great possibility to recover more finds. A large piece of wood, later to be determined as spruce (*Picea* sp.) or larch (*Larix* sp.) was also retrieved. The radiocarbon date of the mandible of the individual demonstrated that these were the oldest human skeletal remains from northern Norway, further stressing the importance of an excavation in the cave. Accordingly, in 2014 two archaeologists, Keth Lind and Roger Jørgensen from Tromsø Museum, performed an archaeological investigation. The access to the cave is somewhat limited, mainly due to the fact that it has partly collapsed at the entrance, causing flooding of the cave, and it was consequently impossible to get to the inner part of the cave without an inflatable canoe. The site where the mandible was found is situated c. 100 m from the cave entrance some 10-15 m from the inner end of the cave. Here the cave was 2x3 m wide and the height was 2.5 m. The mandible was found on the gravel floor, next to a large boulder. The excavation of the surrounding area revealed no cultural layers and only a molar tooth belonging to the mandible, but no other skeletal remains. At some distance from the large boulder and the mandible, where the floor was more clayish, a few small fish bones were retrieved. It was decided that it was too risky to remove the large boulder where the mandible had been found, but also that the likelihood to find further human skeletal remains was small and no further excavations were carried out [65].

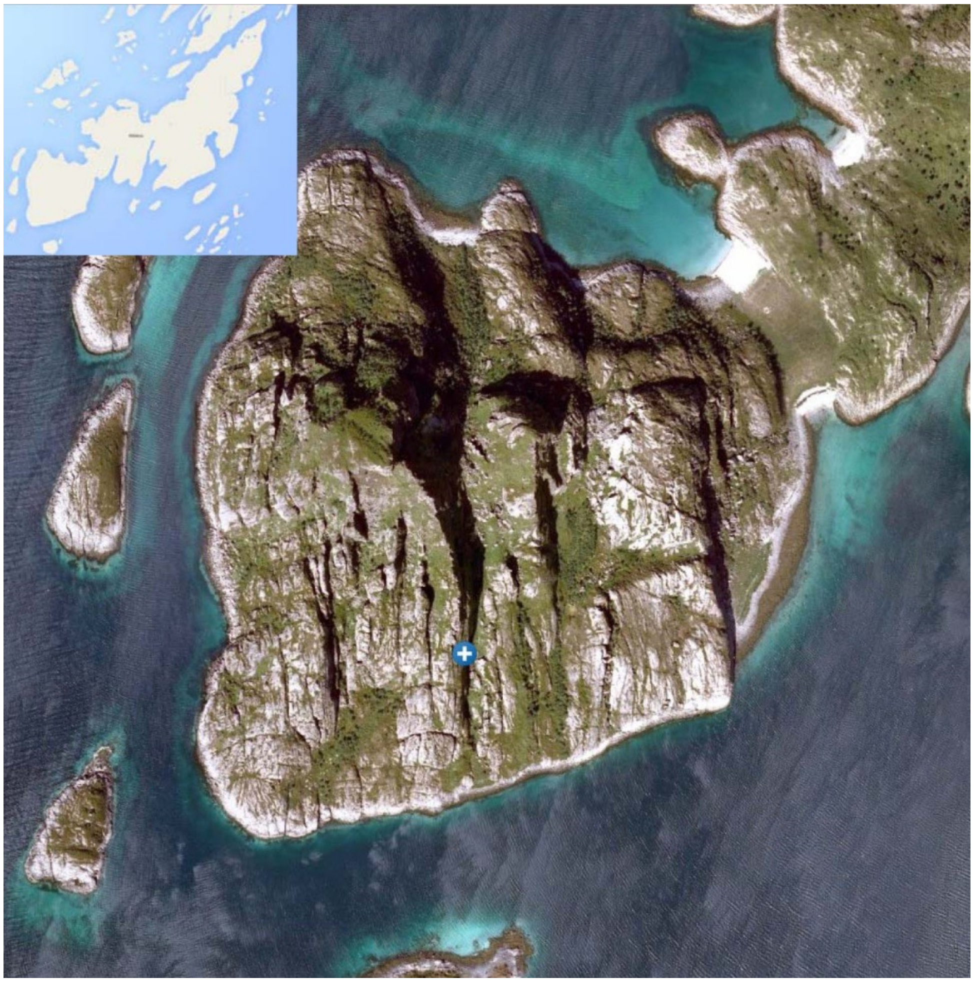

Figure S1.5. Måløya, in Steigen municipality, with the entrance to the cave marked. From [64].

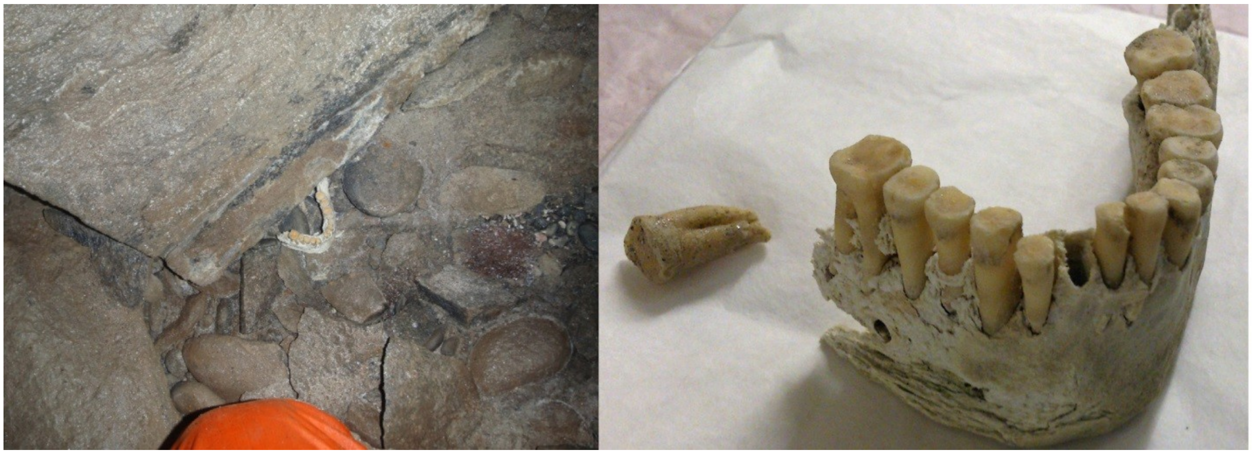

Figure S1.6 Steigen mandible in situ (left, photo by Gunnar Svalbjørg) and close-up (right, photo by Kerstin Lidén)

S1.3.3 Stora Förvar, Sweden

The cave of Stora Förvar, situated on the small island of Stora Karlsö, off the west coast of Gotland in the Baltic Sea, was excavated between 1888 and 1893 when extensive cultural layers of up to 4.5 m thickness of the cave were removed [66] (Figure S1.7). Large amounts of finds dating from the Mesolithic and up to the medieval period were recovered. Radiocarbon dates show that the duration of the Mesolithic occupation lasted from around 9,300 cal BP and the ending around 7,000 cal BP. The zooarchaeological finds indicate that the site was mainly used for seal hunting, but the importance of fish and birds is difficult to evaluate because of the crude recovery techniques [67,68]. The layers were excavated by hand and finds were recovered in 0.3 m thick layers (one foot) in ten different sections/columns (Sw. *parcell*), labelled A to J. The Mesolithic layers (approximately Layers 13-9) produced more than 1,500 kg of bones. The assemblage included scattered human remains from up to ten individuals [69], among them two almost complete crania that unfortunately were lost during storage. The identified individuals are represented by a few skeletal elements each and in some cases, the bones may in fact originate from the same individual.

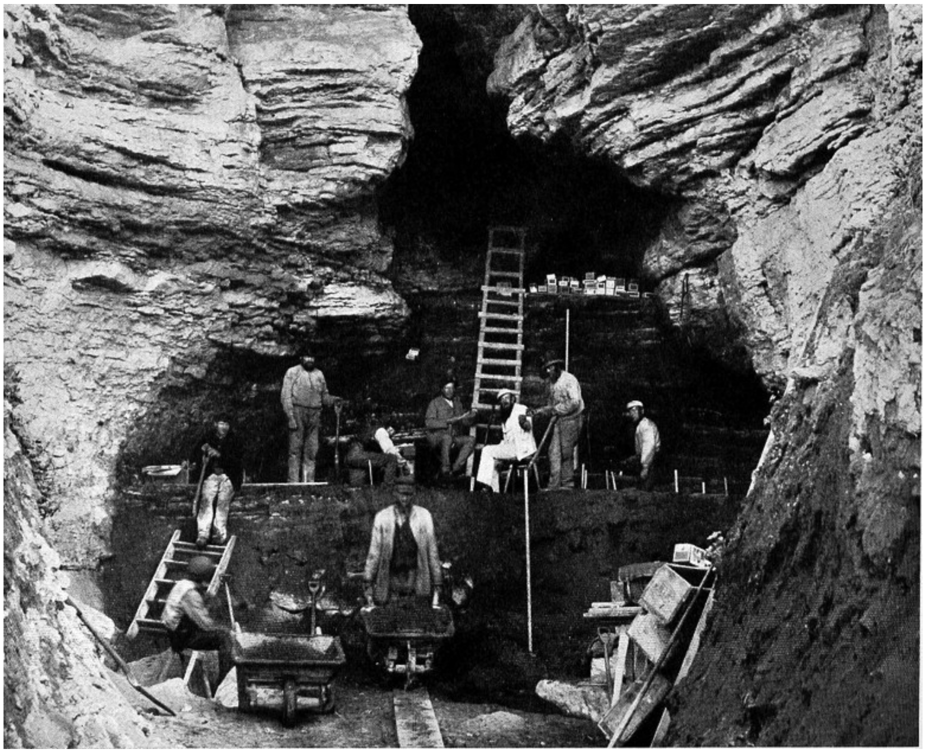

Figure S1.7 Photograph of the excavations of Stora Förvar on Stora Karlsö. Photo: Hjalmar Stolpe. Antiquarian Topographical Archives (ATA), Stockholm.

The field documentation shows that a few human ribs and vertebrae were found in anatomical position indicating the presence of at least one – probably damaged – inhumation burial [69]. It is evident that the long-term use of the cave to some extent has affected the stratigraphic integrity and also the potential to estimate the number of individuals present in the assemblage. It is, however, clear that the human bones originate from at least one infant (maximum 6 months of age) and one or possibly two children at the age of 10-14 years. At least one juvenile male (10-21 years) is present and finally, bones from 3-5 adult individuals (this is a more conservative

estimate than the one presented in Lindqvist and Possnert [69]. We took four samples from adult individuals: SF12 recovered in Section A and Layers 12-14, SF9 in Section G/Layer 9, SF11 from Section G/Layer 11 and SF13 found in Section F/Layer 13.

Lithic tools on the Mesolithic sites on Gotland were mostly manufactured from local Ordovician flint and procured with a simple direct technique in a manner similar to the tradition in the south (the Maglemose techno group I and II [67,70]). There are occasional examples of pressure technique and slotted bone point technology, but these are from a slightly younger period (9,000–8,500 cal BP) than those found in northern Scandinavia. At that time, the pressure technique was also known in Southern Scandinavia. The first pioneers on Gotland seem to have practiced a technology that had more in common with a south-west Scandinavian tradition than the south-east/northern tradition. Such a view is also supported by the find of a core axe of south Scandinavian flint in the Mesolithic culture layers from the Stora Förvar cave [66,67] and of several finds on Gotland of polished Limhamn axes with a south Scandinavian origin.

S1.3.4. Stora Bjers, Sweden

The Stora Bjers burial, located in Stenkyrka parish, is one of the oldest burials on Gotland. We included the c. 45-year-old male that was found in a crouched position in a sandbank [71]. The grave was found in 1954 and the complete burial was brought to the Museum of Gotland where it is exhibited today. Interestingly, a fragmented slotted bone point with an associated regular microblade with a straight profile (Figure S1.8) were found, still attached to the pelvis bone. The regular shape and straight profile of the microblades are strong indicators of the use of pressure technique [58], they likely originate from a pressure blade core. The arrow had likely been shot into the body since only the tip of the point is preserved. The male from Stora Bjers also had severe crush wounds on the skull and left jaw, further indicating a violent death.

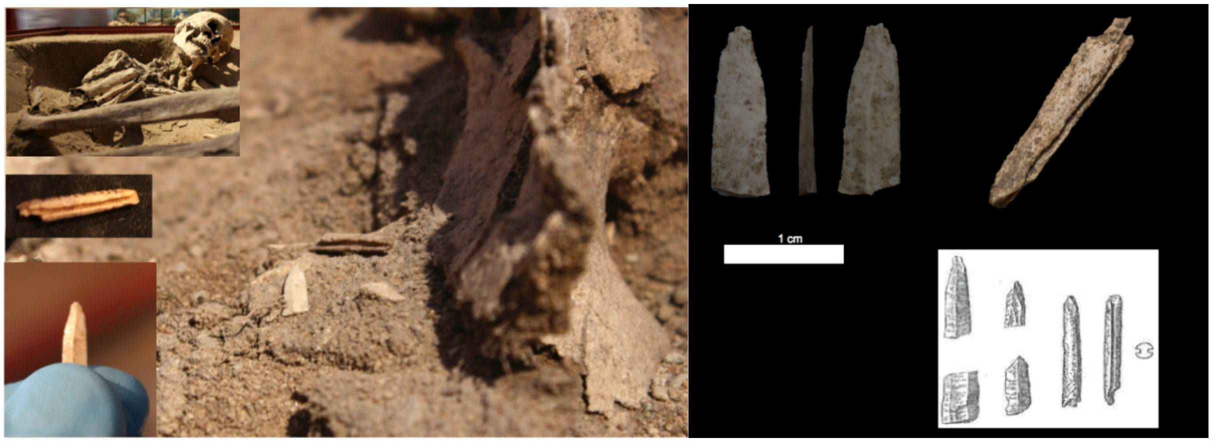

Figure S1.8 Fragmented slotted bone point and two blade fragments was found near the pelvis of the Stora Bjers male (left, photo by Jan Apel, right, photo by Johan Norderäng, Gotland Museum).

S1.3.5. Motala, Sweden

Motala is situated at the river Motala Ström, by the outlet of Lake Vättern in the province of Östergötland, in east central Sweden. Two sites on opposite sides of the river, Kanaljorden and Strandvägen, have been excavated during the past two decades, yielding a large amount of well-preserved organic remains [72]. Radiocarbon dates of faunal and human remains from the two

sites span the period 8,200–6,800 cal BP. The two sites overlap chronologically but they differ contextually. While the Strandvägen site includes a cemetery, associated settlement remains and disarticulated human remains deposited along the shore of the river, the Kanaljorden site consists of a ceremonial wetland deposition of mainly human calvaria on a stone-packing. The lithic assemblages have common features with both the Lihult/Nøstvet Culture of south-west Sweden and south-east Norway [73–75] and the late Kongemose Culture of southern Scandinavia [76–80]. But it also resembles the stone technology found further to the north east [78–80], demonstrating that this is a border zone where different stone technologies met, a cultural mixture contact zone [81]. Previous genetic analyses on the skeletal material from Kanaljorden seem to confirm this hypothesis, suggesting that the population has ancestry both from eastern and western hunter-gatherers [82,83].

Stable carbon and nitrogen isotope analysis demonstrate that all humans were high aquatic-protein consumers, where the aquatic resources ranged from freshwater to brackish/marine origin (Figure S1.9). The overall pattern in the dietary life histories for the Kanaljorden individuals is one of a stable diet throughout childhood, as reflected in $\delta^{13}C$ and $\delta^{15}N$ values [72]. Strontium isotope analysis ($^{87}Sr/^{86}Sr$) of human tooth enamel from both Kanaljorden and Strandvägen shows that there is a clear difference in strontium isotopic compositions between the two sites. While the Strandvägen humans generally exhibit strontium isotope values which fall within the local strontium bio-available range, by contrast, the Kanaljorden individuals generally have strontium isotopic values which fall outside the local range, indicative of a non-local population. The results of stable and radiogenic isotope analysis of human remains from Motala indicate a considerable variation in diet within the two sites as well as a substantial difference in origin between the sites.

S1.4 Stable isotope analysis and diet

Human bones and, where available, teeth from Hummervikholmen, Steigen and Stora Förvar were subjected to stable carbon and nitrogen isotope analysis to investigate dietary patterns. The $\delta^{13}C$ and $\delta^{15}N$ data are reported in Table S1.3 and Figure S1.9. The sampling, collagen extraction and EA-IRMS analysis were performed accordingly:

For the Hummervikholmen and Steigen samples, bone and dentine powder was obtained using a dentist's drill. Collagen was extracted following Brown et al. [84], which includes demineralization in a weak acid (0.25 M HCl) for two days, gelatinization with 0.01 M HCl in 58°C overnight, followed by ultrafiltration to remove remnants <30 kDa, and subsequent lyophilization. Approximately 0.5 mg of collagen was weighed into tin capsules, and combusted in a Carlo Erba NC2500 elemental analyzer connected to a Finnigan MAT Delta+ isotope ratio mass spectrometer run in continuous flow. All the sampling and extractions were performed at the Archaeological Research Laboratory, and the subsequent EA-IRMS analysis took place at the Stable Isotope Laboratory (SIL), Dept. of Geological Sciences, both at Stockholm University. The precision of the $\delta^{13}C$ and $\delta^{15}N$ measurements was ±0.15‰ or better. For the Stora Förvar samples, all sampling, extraction and EA-IRMS analysis were performed at the Beta analytic radiocarbon facility. No detailed data on extraction and measurement precision was reported, other than that collagen was extracted using HCl, followed by treatment with NaOH, and that no ultrafiltration took place.

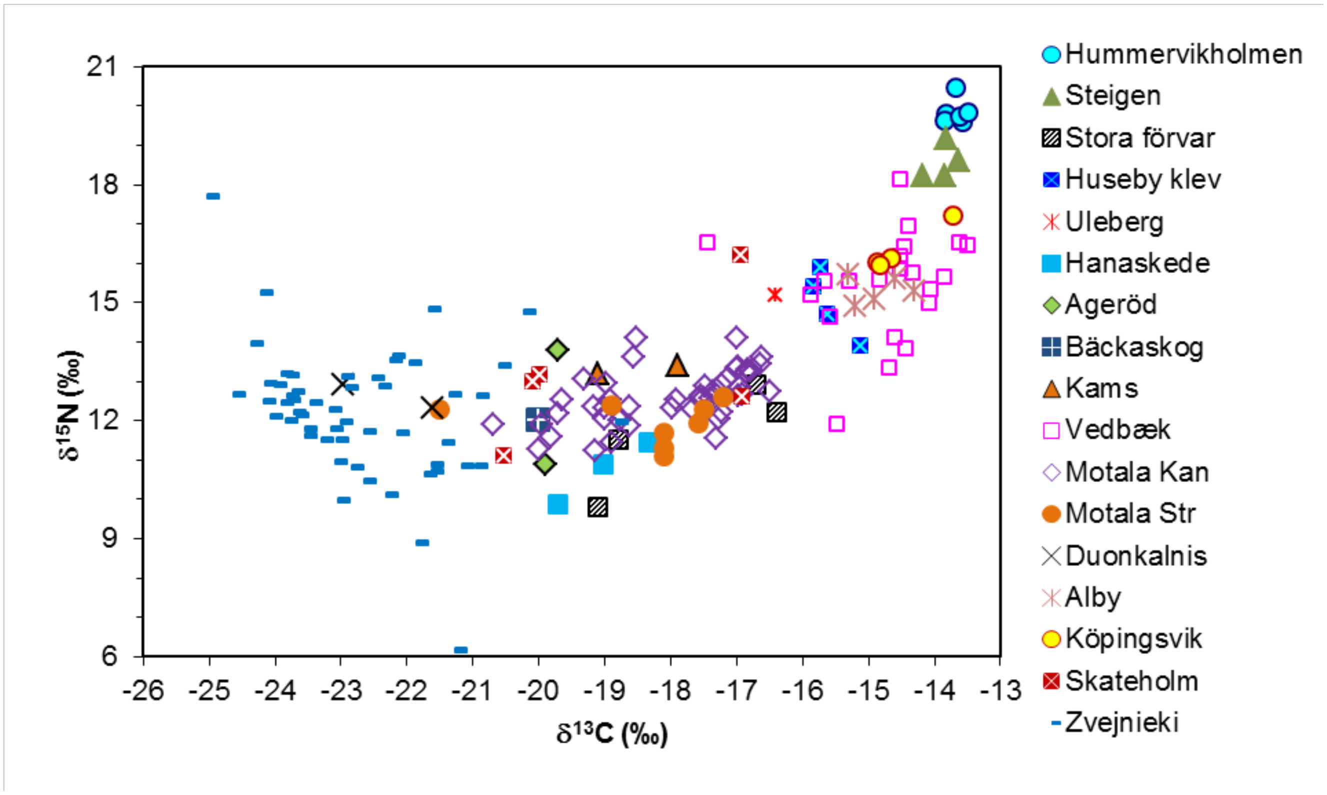

Figure S1.9 Stable carbon and nitrogen isotope data for Scandinavian and East Baltic individuals dated pre-6,000 cal BP. Data from [34,72,85–90], Table S1.2 and Table S1.3.

The Norwegian samples have values ranging between –14.2‰ and –13.5‰ for $\delta^{13}C$, and 18.2–20.5‰ for $\delta^{15}N$, indicative of a massive intake of marine mammal protein in both Hummervikholmen and Steigen (Figure S1.9). Although no faunal reference data from the sites are available to enhance interpretation, the human isotopic signatures are so elevated that they are only comparable to previously analyzed high marine-protein consumers [91,92]. For the two individuals with life history data, Hummervikholmen Individual 1 (Hum1) and Steigen, there is no suggestion of any significant changes in diet during their lifetimes.

The Stora Förvar samples have considerably lower values, between –18.8‰ and –16.4‰ for $\delta^{13}C$ and 9.8–12.9‰ for $\delta^{15}N$. The nitrogen isotope values suggest a diet predominated by protein from fish rather than seals. This is further corroborated by stable isotope data from a Stora Förvar grey seal (*Halichoerus grypus*) of approximately the same radiocarbon age (8100±30 BP, Beta-399028), and $\delta^{13}C$ and $\delta^{15}N$ values of –19.6‰ and 13.1‰, respectively. Because of the trophic-level effect, consumption of seal would result in human $\delta^{15}N$ values around 16‰, which is much higher than the measured human values. With regard to the $\delta^{13}C$ values, the present-day brackish Baltic Sea was at this time in the Ancylus Lake Phase, with freshwater conditions, which should typically render $\delta^{13}C$ values even lower than the ones for the Stora Förvar humans. However, consumption of littoral fish, such as pike and perch, or possibly of migrating seals, may help to explain the range of human $\delta^{13}C$ values.

The genetic data indicate that the samples SF9 and SF13 derive from one single individual. The $\delta^{13}C$ values match (–18.8‰ and –19.1‰, respectively), while the $\delta^{15}N$ values differ by 1.7‰

(11.5‰ and 9.8‰, respectively). This is a larger difference than one would expect if the bones actually derive from the same person. However, since the analysis was performed on different parts of the skeleton, the cranium and the pelvis, respectively, it is possible that this difference merely reflects seasonal variation in protein intake, or a shift in diet [93]. Variation in collagen turnover rate between different bone elements could thus explain why the $\delta^{15}N$ values do not match, and accordingly still be compatible with the assumption that SF9 and SF13 represent one individual. The stable isotope data in this case are not conclusive.

For Stora Bjers there are no IRMS stable isotope data, only an AMS $\delta^{13}C$ measurement, for which the precision is poorer. Given that uncertainty, however, the AMS $\delta^{13}C$ value, –16.1‰, indicates that also this individual may have consumed substantial amounts of aquatic resources.

The stable isotope data from Motala confirms a high level of mobility in hunter-gatherers during the Mesolithic, as demonstrated by intra-individual data from dentine and bone collagen. However, in high marine-protein consumers, such mobility is more difficult to trace, based solely on carbon and nitrogen isotope data. The isotope data from the Hummervikholmen and Steigen individuals do not contradict high mobility. They do, however, demonstrate a pronounced coastal adaptation which requires transportation by/use of boats [31,39,94].

Table S1.2 Previously unpublished stable isotope data for human individuals from Vedbæk (Vedbæk Boldbaner and Henriksholm-Bøgebakken in Denmark) and Donkalnis (Lithuania), plotted in Figure S1.9. All samples were processed at the Archaeological Research Laboratory and SIL, in the same manner as the Hummervikholmen and Steigen samples.

Site	Grave no.	Lab code	Sex (age)	Skeletal element	$\delta^{13}C$ (‰)	$\delta^{15}N$ (‰)	Collagen yield (%)	%C	%C	C/N
Vedbæk Boldbaner	-	VED 29	M	Radius	-15.9	15.2	6.2	42.6	15.2	3.3
Henriksholm-Bøgebakken	1	VED 30	juv. M	Cranium	-14.3	15.8	0.5	40.0	14.4	3.2
Henriksholm-Bøgebakken	3	VED 02	F	Femur	-14.1	15.0	2.6	41.7	14.7	3.3
Henriksholm-Bøgebakken	4	VED 03	M	Ulna	-15.6	14.6	4.5	43.1	15.2	3.3
Henriksholm-Bøgebakken	5	VED 04	M	Femur	-13.5	16.4	1.2	35.9	12.9	3.2
Henriksholm-Bøgebakken	5	VED 48	M	Second mandibular molar	-14.4	16.4	0.6	37.7	12.8	3.4
Henriksholm-Bøgebakken	5	VED 49	M	Third mandibular molar	-14.5	15.8	0.5	36.9	12.7	3.4
Henriksholm-Bøgebakken	6	VED 09	M	Metatarsal	-14.5	16.2	4.2	41.1	14.6	3.3
Henriksholm-Bøgebakken	8A	VED 25	F	Cranium	-14.0	15.3	1.1	40.7	14.8	3.2
Henriksholm-Bøgebakken	8A	VED 24	F	Premolar	-13.9	15.6	1.5	38.9	14.0	3.3
Henriksholm-Bøgebakken	12	VED 28	M	Long bone	-14.8	15.6	1.2	38.4	13.7	3.3
Henriksholm-Bøgebakken	14	VED 05	M	Femur	-13.6	16.5	1.3	36.1	12.9	3.3
Henriksholm-Bøgebakken	14	VED 52	M	Premolar	-15.5	11.9	5.6	40.6	15.4	3.1
Henriksholm-Bøgebakken	15A	VED 13	F	First mandibular molar	-17.4	16.5	1.1	39.6	14.0	3.3
Henriksholm-Bøgebakken	15A	VED 14	F	Second mandibular molar	-15.3	15.5	1.4	40.3	13.6	3.5
Henriksholm-Bøgebakken	19A	VED 23	F	Incisor	-14.6	14.1	0.9	36.0	12.9	3.3
Henriksholm-Bøgebakken	19A	VED 21	F	Mandible	-14.7	13.3	0.4	38.4	13.5	3.3
Henriksholm-Bøgebakken	19A	VED 22	F	Third mandibular molar	-14.4	13.8	1.9	40.5	14.9	3.2
Henriksholm-Bøgebakken	19B	VED 26	infant	Cranium	-14.4	16.9	1.6	42.0	15.3	3.2
Henriksholm-Bøgebakken	19B	VED 18	infant	Second deciduous molar (germ)	-14.5	18.1	2.4	42.6	15.2	3.3
Henriksholm-Bøgebakken	20	VED 06	F	Femur	-15.7	15.5	0.8	33.4	10.8	3.6
Donkalnis	2	DON 02+04	M	Cranium	-23.0	12.9	0.6	16.7	5.6	3.5
Donkalnis	3	DON 01+03	F	Cranium	-21.6	12.3	0.4	21.4	7.7	3.2

S1.5 Radiocarbon dating and reservoir effects

S1.5.1 Radiocarbon reservoir correction

As all the human individuals from the investigated sites included portions of aquatic protein in their diets, it is likely that the radiocarbon dates on the bones have been affected by radiocarbon reservoir effects. It is, consequently, crucial to correct for any reservoir age in order to get an accurate estimate of the true age of a sample. The radiocarbon dates and associated calibrated ranges, with and without reservoir correction for comparison, are presented in Table S1.4.

For the dates on human bone from Hummervikholmen and Steigen, we have applied a maximum reservoir age correction of 380±30 radiocarbon years, based on Mangerud et al. [95]. This correction is based on a 100% marine diet, which seems adequate in both cases, considering their stable isotope signatures. For Hummervikholmen, situated in southern Norway, this correction should be regarded as an absolute maximum, whereas for Steigen, located considerably further

north, it may be closer to the truth. Applying an age offset correction, rather than a ΔR correction, results in differences in calibrated ranges of <30 years, which can be considered irrelevant given the time scale.

For the dates on human bone from Stora Förvar and Stora Bjers, we have applied a reservoir age correction of 70±40 radiocarbon years, following Eriksson [96]. Although this age offset was calculated for human seal hunters on Gotland during the Littorina stage of the Baltic Sea, with admittedly higher salinity, there is no better estimate for the prehistoric Baltic proper that we are aware of. Without more detailed dietary data it is currently not possible to make a more accurate estimate than this rather conservative approximation.

Table S1.3 Stable carbon and nitrogen isotope data (measured by IRMS) for the analyzed samples and individuals. Hummervikholmen data from Skar et al. [34]; Steigen data this study; Stora Förvar data obtained from Beta analytic, reported in conjunction with radiocarbon data. 2016. Nd = no data.

Short name	lab code	Skeletal element	$\delta^{13}C$ (‰)	$\delta^{15}N$ (‰)	collagen yield (%)	%C	%N	C/N
Hum1	SOG105	Cranial fragment	-13.6	19.7	11.2	40.2	14.6	3.2
Hum1	SOG106	Left 2nd maxillary incisor	-13.7	20.5	4.1	39.7	14.0	3.3
Hum	SOG103	Occipital bone (cranium)	-13.8	19.6	7.9	41.0	14.8	3.2
Hum2	SOG107	Left 1st maxillary molar	-13.5	19.8	4.2	39.1	14.1	3.2
Hum	SOG101	Frontal bone (cranium)	-13.8	19.8	5.2	40.5	14.5	3.2
Hum	SOG104	Tibia	-13.6	19.6	6.4	41.0	14.8	3.2
Steigen	STE 01	Right 1st mandibular molar	-13.6	18.6	3.7	40.2	14.8	3.2
Steigen	STE 02	Right 2nd mandibular molar	-14.2	18.2	0.7	41.8	14.7	3.3
Steigen	STE 01	Right 1st mandibular molar	-13.6	18.6	3.7	40.2	14.8	3.2
Steigen	STE 03	Left 3rd mandibular molar	-13.8	18.2	4.0	40.0	14.7	3.2
Steigen	STE 04	Mandible	-13.8	19.2	0.6	37.2	13.4	3.2
SF9	Beta-399027	Parietal bone (cranium)	-18.8	11.5	nd	nd	nd	nd
SF11	Beta-448532	Tibia	-16.7	12.9	nd	43.5	15.8	3.2
SF12	Beta-448531	Femur	-16.4	12.2	nd	43.4	15.9	3.2
SF13	Beta-448533	Os coxae	-19.1	9.8	nd	43.1	15.4	3.3

S1.5.2 Calibrated ranges

There are nine radiocarbon dates from five human skeletal elements from Hummervikholmen (Table S1.4). The archaeological context strongly suggests that the human bones were deposited during one single event, so all the dates should match. Combining all the nine dates does not fulfill the chi-squared test for internal consistency (R_Combine [97]), but the exclusion of the two youngest dates (TUa-2105, TUa-2108) results in a combined date that does not violate the statistical test, giving a combined date of 8,703±27 BP. Alternatively, the selection of the four TRa dates only (which were produced on collagen extracted for stable isotope analysis, fulfilling the collagen quality criteria), results in a combined date of 8,730±34 BP. In both cases, the calibrated 2σ range (95.4% probability) is roughly 9,500–9,300 cal BP. For the Steigen individual, there was only one radiocarbon date, with a 2σ range of approximately 6,000–5,800 cal BP.

From the four Stora Förvar bones, seven radiocarbon dates were produced. The samples do not originate from a closed context, and therefore they should not be considered coeval. The three dates on SF9 and SF13, suspected to derive from one single individual, correspond well and thus support the suspicion, giving an approximate 2σ range of 9,300–9,000 cal BP. Of the four

remaining dates, one (for SF11) is considerably younger (Ua-45742). Since there was a discrepancy between this date and the dates of other individuals, a replicate dating was performed. This replicate date (Beta-448532) falls within the same range as the other two, and is also in accordance with stratigraphic observations. The resulting 2σ range for SF11 and SF12 is around 9,000–8,800 cal BP, or possibly slightly younger, taking the higher $\delta^{13}C$ values for these two individuals into account. The dates further suggest that the horizontal and vertical stratigraphic units (Sections and Layers) within the larger Mesolithic stratum cannot be used for chronological differentiation. The bone from the Stora Bjers individual (SBj) generated a radiocarbon date with an approximate 2σ range of 9,000–8,600 cal BP, that is, roughly contemporaneous with SF11 and SF12.

Table S1.4 Radiocarbon dates sorted by site. Dates have been calibrated with Oxcal 3.10 [98], using the atmospheric curve Intcal13 [99]. The calibrated ranges (2σ, 95.4% probability) are given without and with correction for the marine reservoir effect, respectively. For the Norwegian samples, an offset of 380±30 radiocarbon years were applied, following Mangerud et al. [95], and for the Swedish samples from Gotland, an offset of 70±40 radiocarbon years was applied, following Eriksson [96].

Site	Short name	AMS lab code	Skeletal element	AMS ^{14}C date (BP)	AMS $\delta^{13}C$ (‰)	Calibrated date (cal BP, 2σ, no reservoir correction)	Calibrated date (cal BP, 2σ, reservoir corrected)
Hummervikholmen	Hum1	TRa-954	Cranial fragment	8690±50	-13.0	9865–9539	9471–9225
Hummervikholmen	Hum1	TUa-1257	Cranial fragment	8600±95	-13.4	9890–9438	9461–9011
Hummervikholmen	Hum	TRa-952	Occipital bone (cranium)	8850±65	-13.4	10176–9700	9732–9368
Hummervikholmen	Hum	TUa-2106	Occipital bone (cranium)	8635±75	-13.3	9867–9488	9462–9102
Hummervikholmen	Hum	TRa-951	Frontal bone (cranium)	8665±100	-13.0	10125–9476	9555–9065
Hummervikholmen	Hum	TUa-2105	Frontal bone (cranium)	8095±55	-13.6	9249–8777	8789–8441
Hummervikholmen	Hum	TRa-953	Tibia	8680±85	-13.2	10115–9501	9534–9125
Hummervikholmen	Hum	TUa-2108	Tibia	8455±75	-12.9	9546–9297	9275–8895
Hummervikholmen	Hum	TUa-2107	Femur	8700±70	-12.6	9909–9534	9524–9191
Steigen	Steigen	Beta-349961	Mandible	5450±30	-13.0	6300–6203	5950–5764
Stora Förvar	SF9	Beta-399027	Parietal bone (cranium)	8260 ±30	-18.8	9400–9128	9300–8988
Stora Förvar	SF11	Ua-45742	Tibia	6459±70	-19.1	7495–7255	7434–7147
Stora Förvar	SF11	Beta-448532	Tibia	8070±30	-16.7	9089–8786	9023–8760
Stora Förvar	SF12	Ua-45741	Femur	7952±53	-17.5	8992–8639	8955–8553
Stora Förvar	SF12	Beta-448531	Femur	8080±30	-16.4	9093–8798	9033–8757
Stora Förvar	SF13	Beta-386399	Os coxae	8330±40	-19.3	9467–9152	9421–9110
Stora Förvar	SF13	Beta-448533	Os coxae	8220±30	-19.1	9290–9033	9252–8978
Stora Bjers	SBj	Ua-46147	Tibia	7974±49	-16.1	8997–8649	8963–8579

References

1. Björck S. A review of the history of the Baltic Sea, 13.0-8.0 ka BP. Quaternary international. 1995;27: 19–40.

2. Andrén T, Björck J, Johnsen S. Correlation of Swedish glacial varves with the Greenland (GRIP) oxygen isotope record. Journal of Quaternary Science. 1999;14: 361–371.

3. Mayewski PA, Rohling EE, Curt Stager J, Karlén W, Maasch KA, David Meeker L, et al. Holocene climate variability in Northernmost Europe. Quaternary Research. 2004;62: 243–255.

4. Allen JRM, Long AJ, Ottley CJ, Graham Pearson D, Huntley B. Holocene climate variability in northernmost Europe. Quaternary Science Reviews. 2007;26: 1432–1453.

5. Manninen MA. Culture, Behaviour, and the 8200 cal BP Cold Event: Organisational Change and Culture Environment Dynamics in Late Mesolithic Northern Fennoscandia. 2014. Available:

6. Skar B, Breivik HM. Introduction: Environment and adaptation of forager pioneers in the North-western regions of Europe - The Ecology of Early Settlement in Northern Europe. In: Persson P, Riede F, Skar B, editors. Ecology of early settlement in Northern Europe: conditions for subsistence and survival. Sheffield, UK ; Bristol, CT: Equinox Publishing Ltd; 2017.

7. Larsson L. The earliest settlement of Scandinavia and its relationship with neighbouring areas. Almquist & Wiksell International; 1996.

8. Mörner N-A. The Baltic Ice Lake-Yoldia Sea transition. Quaternary International. 1995;27: 95–98.

9. Jensen J., Bennike O, Witkowski A, Lemke W, Kuijpers A. Early Holocene history of the southwestern Baltic Sea: the Ancylus Lake stage. Boreas. 1999;28: 437–453.

10. Riede F. The Laacher See-eruption (12,920 BP) and material culture change at the end of the Aller{ø}d in Northern Europe. Journal of Archaeological Science. 2008;35: 591–599.

11. Riede F. Climate and demography in early prehistory: using calibrated 14C dates as population proxies. Human Biology. 2009;81: 309–337.

12. Riede F. The resettlement of Northern Europe. In: Cummings V, Jordan P, Zvelebil M, editors. The Oxford Handbook of the Archaeology and Anthropology of Hunter-Gatherers. Oxford University Press; 2014.

13. Gyllencreutz R. Late Glacial and Holocene paleoceanography in the Skagerrak from high-resolution grain size records. Palaeogeography, Palaeoclimatology, Palaeoecology. 2005;222: 344–369.

14. Knutsson K, Knutsson H. The postglacial colonization of humans, fauna and plants in northern Sweden. Arkeologi i norr. 2012;13: 004618–461003.

15. Sørensen R. Late Weichselian deglaciation in the Oslofjord area, south Norway. Boreas. 1979;8: 241–246. doi:10.1111/j.1502-3885.1979.tb00806.x

16. Svendsen JI, Mangerud J. Late Weichselian and holocene sea-level history for a cross-section of western Norway. Journal of Quaternary Science. 2010;2: 113–132.

17. Prøsch-Danielsen L. New light on the Holocene shore displacement curve on Lista, the southernmost part of Norway. Norsk Geografi sk Tidsskrift. 1997;51: 83–101.

18. Prøsch-Danielsen L, Høgestøl M. A Late Weichselian site found at Galta, Rennesøy, southwest Norway. In: Fischer A, editor. Man and Sea in the Mesolithic Coastal Settlement Above and Below Present Sea Level. Oxford: Oxbow Books Limited; 1995. pp. 123–130.

19. Hughes AL, Gyllencreutz R, Lohne ØS, Mangerud J, Svendsen JI. The last Eurasian ice sheets–a chronological database and time-slice reconstruction, DATED-1. Boreas. 2016;45: 1–45.

20. Petersen EB. The human settlement of southern Scandinavia 12 500–8700 cal BC. In: Barton N, Street M, Terberger T, editors. Humans, Environment and Chronology of the Late Glacial of the North European Plain. Mainz: Römisch-Germanisches Zentral museum; 2009. pp. 89–129.

21. Bang-Andersen S. The Myrvatn group, a Preboreal find-complex in southwest Norway. Contributions to the Mesolithic in Europe Papers Presented at the Fourth International Symposium‘ The Mesolithic in Europe. Leuven University Press; 1990. pp. 215–226.

22. Thommessen T. The Early Settlement of Northern Norway. In: Larsson L, editor. The Earliest Settlement of Scandinavia. Stockholm: Almquist & Wiksell International; 1996. pp. 233–240.

23. Åhrberg ES, Pauler I. En Tidligmesolitisk boplats. In: Jaksland L, editor. E18 Brunlanesprosjektet, Bind II: Undersøkte lokaliteter fra tidligmesolitikum og senere. Kulturhistorisk museum, Fornminneseksjonen, Universitet i Oslo; 2012.

24. Breivik HM. Palaeo-oceanographic development and human adaptive strategies in the Pleistocene–Holocene transition: A study from the Norwegian coast. The Holocene. 2014;24: 1478–1490.

25. Kleppe J. Desolate landscapes or shifting landscapes? Late glacial/ early postglacial settlement of northernmost Norway in the light of new data from Eastern Finnmark. In: Riede F, Tallavaara M, editors. Lateglacial and Postglacial Pioneers in Northern Europe BAR International Series 2599. Oxford: Oxford Archaeopress; 2014. pp. 121–145.

26. Fuglestvedt I. The Ahrensburgian Galta 3 site in SW Norway. Dating, technology and cultural affinity. Acta Archaeologica. 2007;78: 87–110.

27. Bjerck HB, Breivik HM, Piana EL, Zangrando AF. Exploring the role of pinnipeds in the human colonization of the seascapes of Patagonia and Scandinavia. In: Bjerck HB, Breivik HM, Fretheim SE, Piana EL, Skar B, Tivoli AM, et al., editors. Equinox Sheffield; 2016. pp. 53–74.

28. Schmitt L, Larsson S, Burdukiewicz J, Ziker J, Svedhage K, Zamon J, et al. Chronological insights, cultural change, and resource exploitation on the west coast of Sweden during the Late Palaeolithic/Early Mesolithic transition. Oxford Journal of Archaeology. 2009;28: 1–27.

29. Schmitt L, Svedhage K. Chronological aspects of the Hensbacka–a group of hunter-gatherers/fishers on the west coast of Sweden during the Pleistocene/Holocene transition: an example of early coastal colonization. Danish Journal of Archaeology. 2015;4: 75–81.

30. Bjerck HB. Norwegian Mesolithic trends: a review. In: Bailey GN, Spikins, editors. Mesolithic Europe. Cambridge University Press; 2008. pp. 60–106.

31. Glørstad H. Where are the missing boats? The pioneer settlement of Norway as long-term history. Norwegian Archaeological Review. 2013;46: 57–80.

32. Damlien H. Eastern pioneers in westernmost territories? Current perspectives on Mesolithic hunter–gatherer large-scale interaction and migration within Northern Eurasia. Quaternary International. 2014;X: 1–12.

33. Damlien H. Between Tradition and adaptation. Long-term trajectories of lithic tool-making in South Norway during the postglacial colonization and its aftermath (c. 9500-7500 cal BC). 2016.

34. Skar B, Lidén K, Eriksson G, Sellevold B. A submerged Mesolithic grave site reveals remains of the first Norwegian seal hunters. In: Bjerck HB, al E, editors. Marine Ventures Archaeological perspectives on Human–Sea Relations. Sheffield: Equinox Publishing; 2016.

35. Möller P, Östlund O, Barnekow L, Sandgren P, Palmbo F, Willerslev E. Living at the margin of the retreating Fennoscandian Ice Sheet: The early Mesolithic sites at Aareavaara, northernmost Sweden. The Holocene. 2013;23: 104–116.

36. Tallavaara M, Pesonen P, Oinonen M. Prehistoric population history in eastern Fennoscandia. Journal of Archaeological Science. 2010;37: 251–260.

37. Tallavaara M, Seppä H. Did the mid-Holocene environmental changes cause the boom and bust of hunter-gatherer population size in eastern Fennoscandia? The Holocene. 2011;22: 215–225.

38. Rankama T, Kankaanpää J. First evidence of eastern Preboreal pioneers in arctic Finland and Norway. Quartär. 2011;58: 183–206.

39. Bjerck HB. Tidligmesolittisk tid og fosnatradisjon 9500–800 BC. In: Bjerck HB, Åstveit LI, editors. Ormen Lange Nyhamna: NTNU Vitenskapsmuseets arkeologiske undersøkkelser. Trondheim: Tapir; 2008. pp. 552–570.

40. Tallavaara M, Manninen M, Pesonen P, Hertell E. Radiocarbon dates and postglacial dynamics in eastern Fenneoscandia. In: Riede F, Tallavaara M, editors. Lateglacial and Postglacial Pioneers in Northern Europe. Oxford: Archaeopress; 2014. pp. 161–175.

41. Veski S, Heinsalu A, Klassen V, Kriiska A, Lõugas L, Poska A, et al. Early Holocene coastal settlements and palaeoenvironment on the shore of the Baltic Sea at Pärnu, southwestern Estonia. Quaternary International. 2005;130: 75–85.

42. Kankaanpää J, Rankama T. Fast or slow pioneers? A view from northern Lapland. In: Riede F, Tallavaara M, editors. Lateglacial and Postglacial Pioneers in Northern Europe, BAR International Series. Oxford: Oxford Archaeopress; 2014. pp. 147–154.

43. Zvelebil M. Innovating hunter-gatherers: the Mesolithic in the Baltic. In: Bailey G, Spikins P, editors. Mesolithic Europe. Cambridge: Cambridge University Press; 2008. pp. 18–59.

44. Pitulko VV, Nikolsky PA, Girya EY, Basilyan AE, Tumskoy VE, Koulakov SA, et al. The Yana RHS site: humans in the Arctic before the last glacial maximum. Science. 2004;303: 52–56.

45. Stroeven AP, Hättestrand C, Kleman J, Heyman J, Fabel D, Fredin O, et al. Deglaciation of Fennoscandia. Quaternary Science Reviews. 2016;147: 91–121.

46. Östlund O. Aareavaara and the Pioneer Period in Northern Sweden. In: Blankholm HP, editor. The early economy and settlement in Northern Europe: pioneering, resource use, coping with change. 2017.

47. Gurina NN. The main stages in the cultural development of the ancient population of the Kola peninsula. Fennoscandia Archaeologica. 1987;IV: 35–49.

48. South A. rworldmap: A New R package for Mapping Global Data. The R Journal. 2011;3: 35–43.

49. Sørensen M, Rankama T, Kankaanpää J, Knutsson K, Knutsson H, Melvold S, et al. The First Eastern Migrations of People and Knowledge into Scandinavia: Evidence from Studies of Mesolithic Technology, 9th-8th Millennium BC. Norwegian Archaeological Review. 2013;46: 19–56.

50. Sørensen M. The arrival and development of pressure blade technology in southern Scandinavia. In: Desrosier P, editor. The emergence of pressure blade making: from origin to modern experimentation. Montreal: Springer; 2012. pp. 237–259.

51. Bjerck HB, Fisher A. The North Sea Continent and the pioneer settlement of Norway. Man and Sea in the Mesolithic. 1995. p. 1.

52. Knutsson H, Knutsson K, Molin F, Zetterlund P. From flint to quartz: Organization of lithic technology in relation to raw material availability during the pioneer process of Scandinavia. Quaternary International. 2016;424: 32–57.

53. Goebel T. The "microblade adaptation" and recolonization of Siberia during the late Upper Pleistocene. In: Elston RG, Kuhn S.-, editors. Archeological Papers of the American Anthropological Association. Virginia: Archaeological Papers of the American Anthropological Association; 2002. pp. 117–131.

54. Inizan M-L. Pressure Débitage in the Old World: Forerunners, Researchers, Geopolitics–Handing on the Baton. In: Desrosier PM, editor. The emergence of pressure blade making: from origin to modern experimentation. New York: Springer; 2012. pp. 11–42.

55. Yi M, Barton L, Morgan C, Liu D, Chen F, Zhang Y, et al. Microblade technology and the rise of serial specialists in north-central China. Journal of Anthropological Archaeology. 2013;32: 212–223.

56. Hartz S, Terberger T, Zhilin M. New AMS-dates for the Upper Volga Mesolithic and the origin of microblade technology in Europe. Quartär. 2010;57: 155–169.

57. Manninen MA, Knutsson K. Lithic raw material diversification as an adaptive strategy—Technology, mobility, and site structure in Late Mesolithic northernmost Europe. Journal of Anthropological Archaeology. 2014;33: 84–98.

58. Pelegrin J. Long blade technology in the Old World: an experimental approach and some archaeological results. In: Apel J, Knutsson K, editors. Skilled production and Social Reproduction. Uppsala: Societas Archaeologica Upsaliensis Stone Studies Uppsala; 2006. pp. 37–68.

59. Sellevold BJ, Skar B. The first lady of Norway. NIKU 1994-1999 Kulturminneforskningens mangfold NIKU Temahefte 31. Oslo: Norsk institutt for kulturminneforskning; 1999. pp. 6–11.

60. Nymoen P, Skar B. The Unappreciated Cultural Landscape: indications of submerged Mesolithic settlement along the Norwegian southern coast. In: Benjamin J, Bonsall C, Pickard C, Fischer A, editors. Submerged Prehistory. Oxford: Oxbow Books; 2011. pp. 38–54.

61. Bennike P, Alexandersen V. Danmarks urbefolkning. Nationalmuseets arbejdsmark. 1997; 143–156.

62. Nilsson Stutz L. The way we bury our dead. Reflections on mortuary ritual, community and identity at the time of the Mesolithic-Neolithic transition. Documenta praehistorica. 2010;37: 33–42.

63. Petersen EB, Jønsson JH, Juel C, Kj\a er A. Diversity of Mesolithic Vedb\a ek. Acta Archaeologica. 2015;86: 7–13.

64. Larsson L. Ethnicity and traditions in Mesolithic mortuary practices of southern Scandinavia. In: Shennan S, editor. Archaeological Approaches to Cultural Identity, Unwin Hyman, London. London: Routledge; 1989. pp. 210–218.

65. Jørgensen R. Måløyhatten. Undersøkelse av funnsted for menneske-kjeve i grotte på Måløyhatten, Måløya, gnr. 30. Steigen commune, Nordland fylke; 2015.

66. Schnittger B, Rydh H, Kolmodin L, Stolpe H. Grottan Stora förvar på Stora karlsö. Wahlström & Widstrand; 1940.

67. Apel J, Storå J. The Pioneer Settlements of Gotland–Early Holocene Maritime Relations in the Baltic Sea Area. In: Persson, editor. The Early Settlement of Northern Europe: An Ecological Approach. Sheffield: Equinox Publishing; p. (in press).

68. Lindqvist C, Possnert G, Burenhult G. The subsistence economy and diet at Jakobs/Ajvide, Eksta parish and other prehistoric dwelling and burial sites on Gotland in long-term perspective. Remote sensing. 1997. pp. 29–90.

69. Lindqvist C, Possnert G. The first seal hunter families on Gotland : on the Mesolithic occupation in the Stora Förvar cave. Current Swedish archaeology. Current Swedish archaeology Stockholm : Swedish Archaeological Society [Svenska arkeologiska samfundet], [1993]-; 1999.

70. Sørensen M. Teknologiske traditioner i Maglemosekulturen. En diakron analyse af maglemosekulturens flækkeindustri. In: Eriksen V, editor. Århus: Jysk Arkæologisk Selskab; 2006. pp. 19–75.

71. Arwidsson G. Stenåldersmannen från Stora Bjärs i Stenkyrka. Arkeologi på Gotland, Gotlandica 14: 17. 1979;25.

72. Eriksson G, Frei KM, Howcroft R, Gummesson S, Molin F, Lidén K, et al. Diet and mobility among Mesolithic hunter-gatherers in Motala (Sweden)-The isotope perspective. Journal of Archaeological Science: Reports. 2016

73. Nordqvist B. Coastal adaptations in the Mesolithic: a study of coastal sites with organic remains from the Boreal and Atlantic periods in Western Sweden. Gothenburg University. 2000.

74. Andersson S, Wigforss J. Senmesolitikum i Göteborgs-och Alingså asområdena (GOTARC. Serie C. Arkeologska skrifter, 58). Göteborg University,; 2004.

75. Glørstad H. The structure and history of the Late Mesolithic societies in the Oslo fjord area 6300-3800 BC. Lindome: Bricoleur Press; 2010.

76. Sørensen SA. Kongemosekulturen i Sydskandinavien. Frederikssund: Egnsmuseet Færgegården; 1996.

77. Johansson AD. Ældre stenalder i Norden. Farum: SDA; 2000.

78. Åkerlund A. Human responses to shore displacement: living by the sea in Eastern Middle Sweden during the Stone Age. UV Stockholm, Avd. för arkeologiska undersökningar, Riksantikvarieämbetet; 1996.

79. Lindgren C. Människor och kvarts: sociala och teknologiska strategier under mesolitikum i östra Mellansverige. 2004.

80. Pettersson M, Wikell R. Tidigmesolitiska säljägare i Tyresta för 10 000 år sedan. Späckbetong, gråsäl och tomtning på en kobbe i Ancylussjön 120 km från fastlandet. Fornvännen. 2013;108: 73–92.

81. Carlsson T, Gruber G, Molin F. The Mesolithic in Östergötland. An Introduction. In: Gruber G, editor. Identities in Transition Mesolithic Strategies in the Swedish Province of Östergötland. Linköping: Riksantikvarieämbetet; 2005. pp. 8–23.

82. Lazaridis I, Patterson N, Mittnik A, Renaud G, Mallick S, Kirsanow K, et al. Ancient human genomes suggest three ancestral populations for present-day Europeans. Nature. 2014;513: 409–413. doi:10.1038/nature13673

83. Haak W, Lazaridis I, Patterson N, Rohland N, Mallick S, Llamas B, et al. Massive migration from the steppe was a source for Indo-European languages in Europe. Nature. 2015;522: 207–211.

84. Brown TA, Nelson DE, Vogel JS, Southon JR. Improved collagen extraction by modified Longin method. Radiocarbon. 2006;30: 171–177.

85. Lidén K. A dietary perspective on Swedish hunter-gatherer and Neolithic populations. Laborativ Arkeologi. 1996;9: 5–23.

86. Sten S. Barumkvinnan: nya forskningsrön. Fornvännen. 2000;

87. Eriksson G, Lidén K. Skateholm revisited: New stable isotope evidence on humans. In: Eriksson, Gunilla, editor. Norm and difference: Stone Age dietary practice in the Baltic region. 2003.

88. Lidén K, Eriksson G, Nordqvist B, Gotherstrom A, Bendixen E. “ The wet and the wild followed by the dry and the tame”-or did they occur at the same time? Diet in Mesolithic-Neolithic southern Sweden. Antiquity. 2004;78: 23.

89. Eriksson G. Stable isotope analysis of human and faunal remains from Zvejnieki. Acta archaeologica Lundensia Series in 8. 2006; 183–215.

90. Eriksson G, Linderholm A, Fornander E, Kanstrup M, Schoultz P, Olofsson H, et al. Same island, different diet: cultural evolution of food practice on Öland, Sweden, from the Mesolithic to the Roman Period. Journal of Anthropological Archaeology. 2008;27: 520–543.

91. Chu PP. Dietary variation among the prehistoric Asiatic Eskimo. Simon Fraser University. 1998.

92. Honch NV, McCullagh JS, Hedges RE. Variation of bone collagen amino acid δ13C values in archaeological humans and fauna with different dietary regimes: developing frameworks of dietary discrimination. American journal of physical anthropology. 2012;148: 495–511.

93. Eriksson G, Lidén K. Dietary life histories in Stone Age northern Europe. Journal of Anthropological Archaeology. 2013;32: 288–302.

94. Bjerck HB. Colonizing seascapes: comparative perspectives on the development of maritime relations in Scandinavia and Patagonia. Arctic Anthropology. 2009;46: 118–131.

95. Mangerud J, Bondevik S, Gulliksen S, Karin Hufthammer A, Høisæter T. Marine 14C reservoir ages for 19th century whales and molluscs from the North Atlantic. Quaternary Science Reviews. 2006;25: 3228–3245.

96. Eriksson G. Part-time farmers or hard-core sealers? Västerbjers studied by means of stable isotope analysis. Journal of Anthropological Archaeology. 2004;23: 135–162.

97. Ramsey CB, Others. Bayesian analysis of radiocarbon dates. Radiocarbon. 2009;51: 337–360.

98. Ramsey CB. OxCal Program v3. 10. Online at: http://www rlaha ox ac uk/orau. 2005;

99. Reimer PJ, Bard E, Bayliss A, Beck JW, Blackwell PG, Bronk Ramsey C, et al. IntCal13 and Marine13 radiocarbon age calibration curves 0-50,000 years cal BP. 2013;
