# Supplementary material for: Population genomics of Mesolithic Scandinavia: Investigating early postglacial migration routes and high-latitude adaptation
Source: PLoS Biol. 2018 Jan 9;16(1):e2003703. doi: 10.1371/journal.pbio.2003703 (PMC5760011; doi:10.1371/journal.pbio.2003703)
Supplement: S2 Text — (PDF) [file pbio.2003703.s007.pdf]

S2 Details on DNA Sample preparation

Table S2.1 Summary of the lab work and results from sequencing of the Mesolithic samples.

Sample name	Bone element	Number of Extracts	Number of libraries	Average read length	Average non-duplicate proportion human [%] (Minimum mapping quality 30)
Hum1	Left 2nd maxillary incisor	2	10	60.639	3.4
Hum2	Left 1st maxillary molar	1	5	82.9063	5.6
Steigen	Mandibular molar	1	1	92.821	26.8
SF9	Parietal bone, Os coxae	8	29	74.447	3.1
SF11	Tibia	4	11	79.8543	1.7
SF12_damage repair	Femur	126*	258	74.9771	4.7
SF12	Femur	15	25	70.9709	6.8
SBj	Teeth, right M1 and M2 and M3 mandible, Tibia	5	15	78.5173	3.4

*This includes the initial 15 extracts for which also non-damage repair libraries were built.

Table S2.2 Summary of two Ajvide samples previously published in Skoglund *et al.* [1] for which additional data was generated.

					Mitochondrial contamination estimate					
Sample	Average read length	Genome coverage	Mitochondrial coverage	Genetic sex	Point estimate	Informative sites	Consenus alleles	Total alleles	Lower C.I	Higher C.I
Ajv58	64.386	2.70	378.97	XY	2.90	5	1505	1550	2.07	3.74
Ajv70	61.365	1.34	108.56	XY	1.61	5	551	560	0.57	2.65

The endogenous DNA content in an ancient sample can vary greatly between different bone elements, as observed in [2,3], however even within the same bone element the proportion of endogenous DNA can vary [4,5]. Here we make the same observation, even though all extracts were generated from powder sampled from a 10cm^2 area of the femur the proportion of human DNA varied between 2 and 14%, except for a few extracts where some of the libraries generated were subjected to WGC show an average human proportion of 20% (Table S2.3). The average proportion of human DNA for each extract was calculated after removal of duplicates.

Table S2.3 proportion of human DNA and average read length for extracts for which damage repair was done.

Extract	Non-duplicate human proportion	Read length
f1	0.0568636598	72.0681
f2	0.0655421383	75.6630625
g1	0.0487412387	75.95864
g2	0.0582619406	78.7907875
h1	0.0742552347	73.45664
h2	0.0851679098	79.2090625
i1	0.0388759714	74.79602
i2	0.0434989209	75.993525

j1	0.0389066373	71.93756
j2	0.0514995509	71.1007875
k1	0.0319647247	74.22806
k2	0.0381907159	76.683275
l1	0.0302095219	75.86332
l2	0.0335597919	76.181225
m1	0.0496837664	81.58628
m2	0.0605230021	79.3211625
n1	0.0376078974	71.80102
n2	0.0747283112	75.343825
o1	0.0271022471	73.86608
o2	0.032300346	76.2626375
s	0.0102179429	68.3368
q	0.0194859721	74.2623571429
t1	0.0553612316	78.79745
t2	0.0562593462	83.47505
t3	0.0475910668	76.0176666667
t4	0.0415985974	86.1800888889
t5	0.0243231032	76.9118125
t6	0.0310185891	75.2801111111
t7	0.0189539249	74.9423777778
t8	0.0352912319	81.48415
u1	0.0339274622	78.7452166667
u2	0.0295915419	80.1884916667
u3	0.0337740423	78.258825
u4	0.0627580416	77.1813846154
u5	0.0571196928	66.2621888889
u6	0.0557168508	72.52325
u7	0.0619772871	78.0147333333
u8	0.042687556	76.3665142857
v1	0.0894996154	86.7739166667
v2	0.0716081869	81.0637125
v3	0.0436063595	88.65478
v4	0.0425226296	78.1164625
v5	0.0485653944	72.2904
v6	0.0475934678	80.149575
v7	0.0390909118	74.27605
v8	0.0149852434	75.9553571429
w1	0.0454193567	88.8353
w2	0.0222316397	59.6168
w3	0.0169109659	80.5732166667
w4	0.0378082747	64.6882
w5	0.0513133156	82.05932
w6	0.0309709702	60.5789666667
w7	0.0657496994	57.1689

w8	0.0499932644	64.5477
y1	0.0690055037	70.51875
y2	0.0657477034	74.6471666667
y3	0.0278241066	82.696825
y4	0.0290456901	64.77865
y5	0.0389971138	65.16305
y7	0.0381935681	78.70615
y8	0.0367056605	81.9648
x1	0.0327776036	80.0742833333
x2	0.0616322748	78.5798714286
x3	0.0278713637	84.03546
x4	0.0496015207	82.5797571429
x5	0.0895367753	82.1414571429
x6	0.0632386086	83.2141
x7	0.0613428268	84.6820166667
x8	0.1000110298	81.43512
z1	0.0536108278	76.785975
z2	0.050434322	85.89214
z3	0.0450561749	79.46525
z4	0.0511667235	78.0629
z5	0.0315288233	86.9599333333
z6	0.0521637816	68.0875
z7	0.058310996	73.6346
z8	0.0833501902	70.0847666667
ls01	0.0394372544	83.9703
ls02	0.0532810862	86.9569333333
ls03	0.0574895137	81.6832
ls04	0.0350745193	84.2945
ls05	0.0661043144	103.8751333333
ls06	0.0677302422	85.0050666667
ls07	0.0756146962	91.6787666667
ls08	0.0534335421	85.5634666667
ls09	0.077843796	87.8827
ls10	0.0690244817	79.72172
ls11	0.032577006	100.4325333333
ls12	0.04943783	106.5389333333
ls13	0.0678540753	92.67056
ls14	0.0746394852	92.49592
ls15	0.1008940535	86.22418
ls16	0.0622933008	97.734925
ls17	0.0489750257	95.362
ls18	0.0473854069	93.418975
ls19	0.0543604918	104.4772
ls20	0.0478468645	93.2154
ls21	0.019070989	89.3835333333

ls22	0.0323669834	83.7892
ls23	0.0224001226	93.5419666667
ls24	0.0219683976	83.1180666667
ls25	0.0598419126	81.68712
ls26	0.0581526455	72.48468
ls27	0.0496427629	76.380025
ls28	0.0391103919	79.221575
ls29	0.0364761661	75.604925
ls30	0.0285452055	82.5539666667
ls31	0.0525504216	75.4277625
ls32	0.0523450033	72.78292
ls33	0.0383775415	71.79542
ls34	0.0444801881	66.2718
ls35	0.0368812536	72.338
ls36	0.0258846489	75.7962
ls37	0.026390841	71.8117909091
ls38	0.0184480892	74.56752
ls39	0.0210260316	74.9166222222
ls40	0.0215502871	72.8480333333
em	0.0369780467	86.1493925581

References

1. Skoglund P, Malmstrom H, Omrak A, Raghavan M, Valdiosera C, Gunther T, et al. Genomic Diversity and Admixture Differs for Stone-Age Scandinavian Foragers and Farmers. Science. 2014;344: 747–750. doi:10.1126/science.1253448

2. Pinhasi R, Fernandes D, Sirak K, Novak M, Connell S, Alpaslan-Roodenberg S, et al. Optimal Ancient DNA Yields from the Inner Ear Part of the Human Petrous Bone. PLoS ONE. 2015;10: e0129102. doi:10.1371/journal.pone.0129102

3. Gamba C, Jones ER, Teasdale MD, McLaughlin RL, Gonzalez-Fortes G, Mattiangeli V, et al. Genome flux and stasis in a five millennium transect of European prehistory. Nature Communications. 2014;5: 5257. doi:10.1038/ncomms6257

4. Seguin-Orlando A, Korneliussen TS, Sikora M, Malaspinas A-S, Manica A, Moltke I, et al. Genomic structure in Europeans dating back at least 36,200 years. Science. 2014;346: 1113–1118. doi:10.1126/science.aaa0114

5. Fu Q, Li H, Moorjani P, Jay F, Slepchenko SM, Bondarev AA, et al. Genome sequence of a 45,000-year-old modern human from western Siberia. Nature. 2014;514: 445–449. doi:10.1038/nature13810
