# Supplementary material for: Population genomics of Mesolithic Scandinavia: Investigating early postglacial migration routes and high-latitude adaptation
Source: PLoS Biol. 2018 Jan 9;16(1):e2003703. doi: 10.1371/journal.pbio.2003703 (PMC5760011; doi:10.1371/journal.pbio.2003703)
Supplement: S3 Text — NGS, next-generation sequencing. (PDF) [file pbio.2003703.s008.pdf]

S3 Processing of NGS data

S3.1 Authentication of aDNA

Each library, with an estimated contamination lower than 6% using the method developed by Green et al. [1] (see description below) (10 Hum1 libraries, 5 Hum2 libraries, 11 SBj libraries, 29 SF9 libraries, 11 SF11 libraries, 254 SF12 damage-repair libraries, and 1 Steigen library), was then merged (per sample) into a final bam-file using samtools merge [2]. The mtDNA contamination per library was estimated to between 0-4.5% (Table 1 and S4.1) after removal of potentially contaminated libraries (n=4) in SF12 (contamination higher than 5%). The data from all libraries also show the, for aDNA, characteristic deamination patterns towards the fragment-end [3] (Figure S3.1).

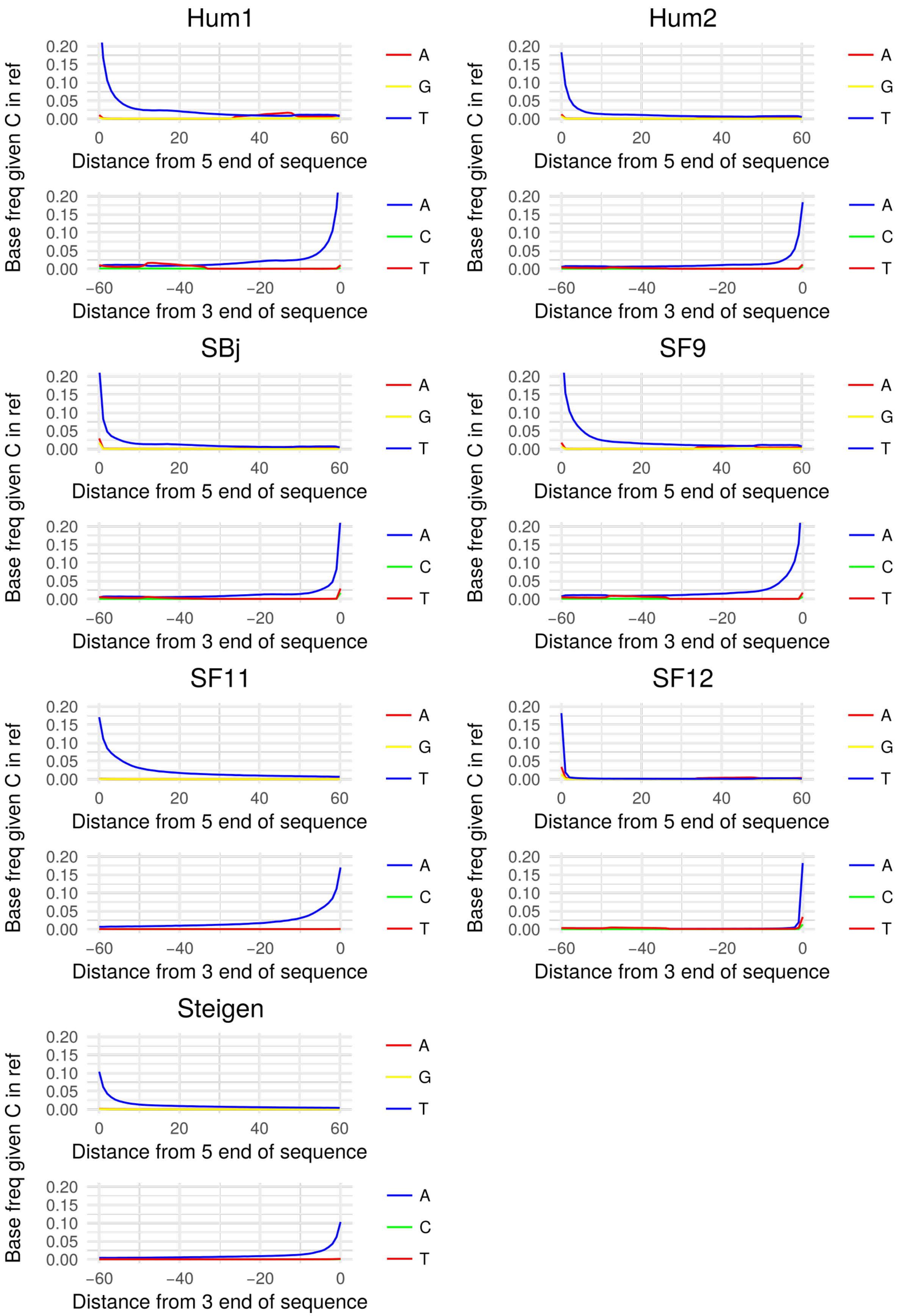

Figure S3.1 Damage patterns for all newly sequenced samples.

S3.2 Novel variants in SF12

The high sequencing coverage and the UDG treatment of the SF12 individual made it possible to call new variants in her genome. The number of unique variants per genome largely differed between populations among the individuals sequenced in the 1000 genomes project. The numbers ranged from an average of about 6,000 singletons per sequenced FIN individual to slightly more than 20,000 per sequenced individual from LWK [4]. SF12 represents a population that contributed to modern day European´s ancestry but with no direct continuity to any extant population [5–7]. Therefore, it is likely that some of the genetic variation present in SF12 has been lost since.

First, the base qualities of all Ts in the first five base pairs of each read together with all As in the last five base pairs were set to 2. This was done in order to avoid residual deamination among the last bases of each fragment. Further, we used Picard [8] to add read groups to the files. Indel realignment was conducted with GATK 3.5.0 [9] using indels identified in phase 1 of the 1000 genomes project as reference [4]. Finally, GATK's UnifiedGenotyper was applied to call diploid genotypes with the parameters -stand_call_conf 50.0, -stand_emit_conf 50.0, -mbq 30, -contamination 0.02 and --output_mode EMIT_ALL_SITES using dbSNP version 142 as known SNPs.

GATK's VariantFiltration was used to filter variants applying the conservative filters QD < 3.0 || FS > 60.0 || MQ < 35.0 || MQRankSum < -12.5 || ReadPosRankSum < -8.0 || MQ0 >=5 and GQ < 50 || DP > 100. Call sets were created with different minimum coverages between 10 and 80. Last, we used bedtools [10] to restrict to regions uniquely mappable with 35 base pair reads [11] and evaluated the results using GATK's VariantEval. The transition-transversion ratio (Ti/Tv ratio) of called novel SNPs can be used to assess the quality of the SNP calling as the expected ratio would be between 2.0 and 2.1 for human whole genome sequencing data [12,13]. Comparing the transition-transversion ratio to these expectations and to comparable sites in dbSNP, we observe that the Ti/Tv ratio of novel SNPs in SF12 is too low for minimum coverages <45 (Figure S3.2). The Ti/Tv ratio grows slightly for higher coverage cutoffs but it remains close to the expected range and for minimum coverages >90, the estimates are noisy due to the low total number of novel SNPs (Figure S3.2b). This likely suggests an enrichment of false positives as the Ti/Tv ratio of random calls would be 0.5. We conclude that restricting the calls of new SNPs to sites with at least 55x coverage should provide high quality calls (Figure S3.2). This resulted in 5,502 autosomal SNP sites not reported in dbSNP. As this analysis excludes more than 40% of the human genome, we estimate that the total number of unknown SNP sites in SF12 would be approximately 10,600. This number is similar to the numbers of singletons found per European genome in the 1000 genomes project: 6,000 SNPs per Finnish genome, 9,500 SNPs per British genome, 12,000 SNPs per Spanish or CEU genome, and 14,500 per Tuscan genome [4]. A direct comparison to these numbers, however, is difficult since sample sizes, sequencing coverage and data processing differed between the studies. Furthermore, demographic effects may have effected the number of private variants in Finns [14].

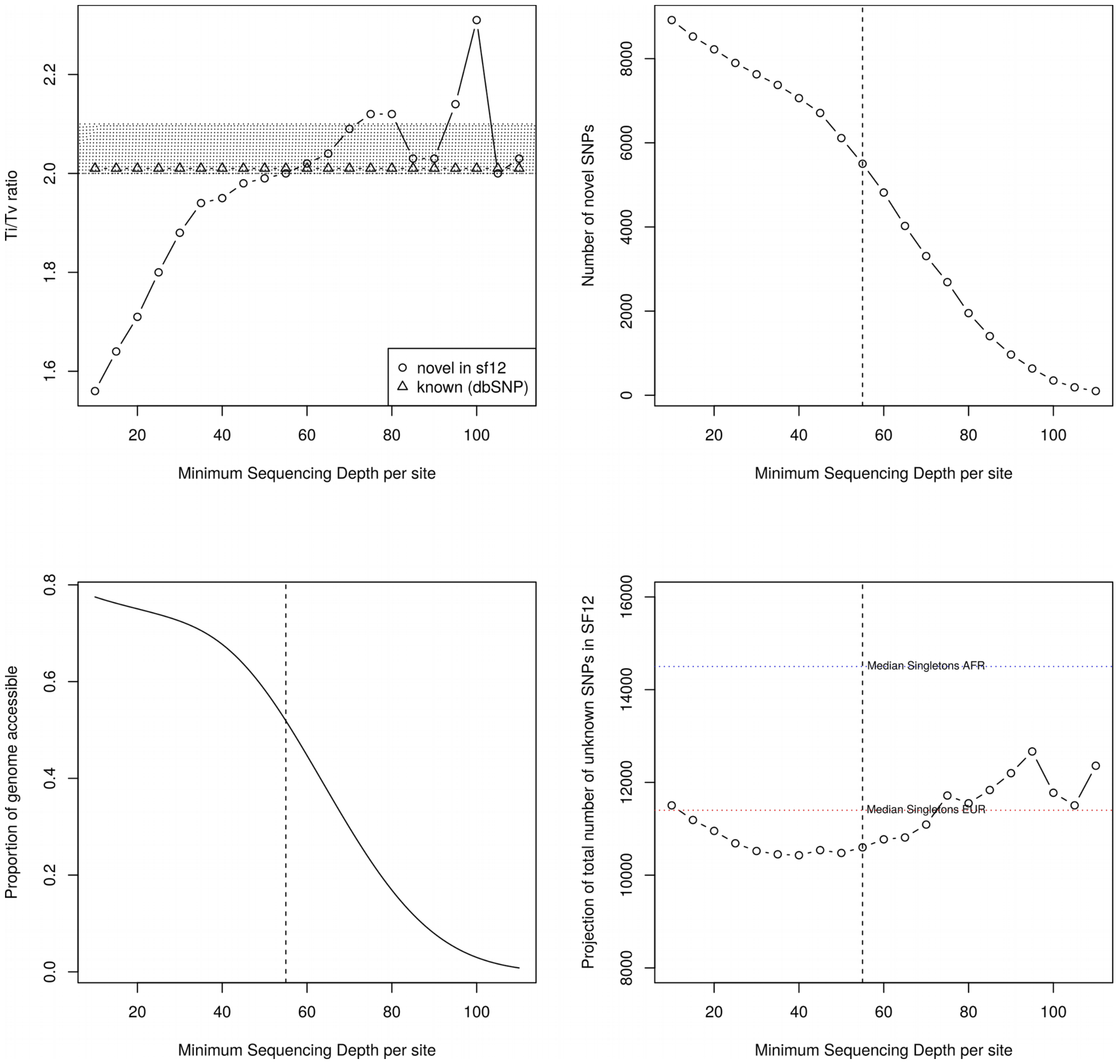

Figure S3.2 Quality control and number of novel variants in SF12. (a) Transition/transversion ratio of novel and known SNPs as a function of the minimum sequencing depth per site considered in the analysis. The shaded area shows the expected range for human whole genome sequencing data [12,13]. (b) Number of novel variants called as a function of the minimum sequencing depth per site. (c) Proportion of the genome accessible when applying a minimum sequencing depth filter as a function of the minimum sequencing depth per site. (d) Projected number of novel variants (assuming the full human reference genome was accessible for SF12) as a function of the minimum sequencing depth per site. Dotted horizontal lines represent median numbers from the 1000 genomes project.

We also annotated the previously unobserved variants in SF12's genome using SNPeff 4.2 [15]. The novel SNPs are more common in genic regions than known SNPs also called in SF12 (Figure S3.3) which suggests that these novel variants could be younger and that they have not been subject to as much purifying selection. Only four of the novel SNPs in SF12 are annotated as "high impact" (Table S3.1), which includes such annotations as START_LOST, STOP_GAINED and mutations at splice sites [15]. One of those high impact SNPs falls on a splice acceptor site in *RP11-110I1.12*, the second SNP adds a stop codon to *REP15* and the third SNP affects a splice donor site in *PIGW*. Finally, a SNP affects a protein-protein binding site in *HSPA2*, a heat shock protein known to be involved in response to cold and heat. We did not find sequencing reads supporting these high impact variants in the other SHGs which suggests that they are either at low frequencies in the SHG population or some of them represent false positives. In order to obtain an upper bound on how many of the novel variants in SF12 are singletons, we checked all other SHGs at all 3,883 SNP sites that might not be due to deamination damage (reference allele C and alternative allele T or reference allele G and alternative allele A). 3,874 of these SNP sites were covered by reads in at least one of the other SHGs and at 668 sites at least one of the reads represented the alternative allele. This suggests that at least 17.2% of those novel variants were more frequent in Mesolithic Scandinavians. Extending this analysis to other prehistoric genomes (Table S3.2) studied in this paper increases this percentage to 24.2%.

Table S3.1: Novel "high impact SNPs" as suggested bu SNPeff.

Chromosome	Position	Reference allele	Alternative allele	Gene	Consequence
11	118867987	C	G	RP11-110I1.12	splice_acceptor_variant&intron_variant
12	27849733	A	T	REP15	stop_gained
14	65008255	C	T	HSPA2	protein_protein_contact
17	34891442	T	C	PIGW	splice_donor_variant&intron_variant

Table S3.2: Individuals screened for presence of variants found in SF12

Individual
Bichon
Labrana1
KO1
NE1
NE5
NE6
NE7
CB13
Ajvide58
Ajvide70
Zv313
Zv93
Zv121
Stuttgart
Loschbour
NE1
I0061
I0124
I0211
I0707
I0708
I0709
I0736
I0744
I0745
I0746
I1096
I1097
I1098
I1101
I1103
I1579
I1580
I1581
I1583
I1585
I0025
I0026
I0046
I0054
I0100

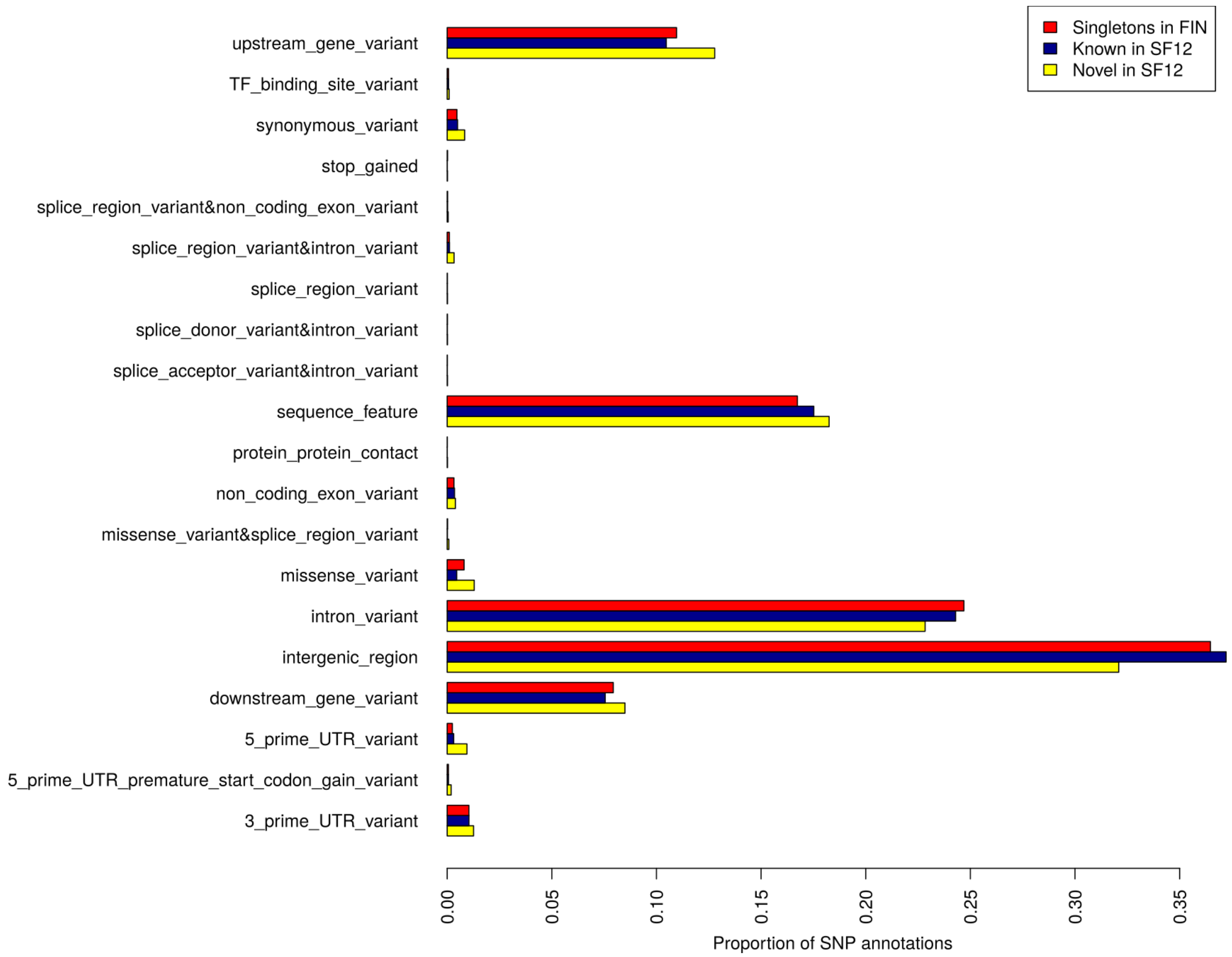

Figure S3.3 Annotation of novel and known SNPs called in SF12 compared to all singletons in the 1000 genomes FIN population.

References

1. Green RE, Malaspinas A-S, Krause J, Briggs AW, Johnson PLF, Uhler C, et al. A Complete Neandertal Mitochondrial Genome Sequence Determined by High-Throughput Sequencing. Cell. 2008;134: 416–426. doi:10.1016/j.cell.2008.06.021

2. Li H, Handsaker B, Wysoker A, Fennell T, Ruan J, Homer N, et al. The Sequence Alignment/Map format and SAMtools. Bioinformatics. 2009;25: 2078–2079. doi:10.1093/bioinformatics/btp352

3. Sawyer S, Krause J, Guschanski K, Savolainen V, Pääbo S. Temporal Patterns of Nucleotide Misincorporations and DNA Fragmentation in Ancient DNA. Lalueza-Fox C, editor. PLoS ONE. 2012;7: e34131. doi:10.1371/journal.pone.0034131

4. Auton A, Abecasis GR, Altshuler DM, Durbin RM, Abecasis GR, Bentley DR, et al. A global reference for human genetic variation. Nature. 2015;526: 68–74. doi:10.1038/nature15393

5. Lazaridis I, Patterson N, Mittnik A, Renaud G, Mallick S, Kirsanow K, et al. Ancient human genomes suggest three ancestral populations for present-day Europeans. Nature. 2014;513: 409–413. doi:10.1038/nature13673

6. Skoglund P, Malmstrom H, Omrak A, Raghavan M, Valdiosera C, Gunther T, et al. Genomic Diversity and Admixture Differs for Stone-Age Scandinavian Foragers and Farmers. Science. 2014;344: 747–750. doi:10.1126/science.1253448

7. Skoglund P, Malmstrom H, Raghavan M, Stora J, Hall P, Willerslev E, et al. Origins and Genetic Legacy of Neolithic Farmers and Hunter-Gatherers in Europe. Science. 2012;336: 466–469. doi:10.1126/science.1216304

8. Broad Institute. Picard tools. https://broadinstitute.github.io/picard/. 2016; Available: http://broadinstitute.github.io/picard/

9. Schmidt S. (GATK) The Genome Analysis Toolkit: A MapReduce framework for analyzing next-generation DNA sequencing data. Proceedings of the International Conference on Intellectual Capital, Knowledge Management & Organizational Learning. 2009;20: 254–260. doi:10.1101/gr.107524.110.20

10. Quinlan AR, Hall IM. BEDTools: A flexible suite of utilities for comparing genomic features. Bioinformatics. 2010;26: 841–842. doi:10.1093/bioinformatics/btq033

11. Prüfer K, Racimo F, Patterson N, Jay F, Sankararaman S, Sawyer S, et al. The complete genome sequence of a Neanderthal from the Altai Mountains. Nature. 2014;505: 43–9. doi:10.1038/nature12886

12. Freudenberg-Hua Y. Single Nucleotide Variation Analysis in 65 Candidate Genes for CNS Disorders in a Representative Sample of the European Population. Genome Research. 2003;13: 2271–2276. doi:10.1101/gr.1299703

13. DePristo MA, Banks E, Poplin R, Garimella KV, Maguire JR, Hartl C, et al. A framework for variation discovery and genotyping using next-generation DNA sequencing data. Nature Genetics. 2011;43: 491–498. doi:10.1038/ng.806

14. Chheda H, Palta P, Pirinen M, McCarthy S, Walter K, Koskinen S, et al. Whole-genome view of the consequences of a population bottleneck using 2926 genome sequences from Finland and United Kingdom. Eur J Hum Genet. 2017;25: 477–484. doi:10.1038/ejhg.2016.205

15. Cingolani P, Platts A, Wang LL, Coon M, Nguyen T, Wang L, et al. A program for annotating and predicting the effects of single nucleotide polymorphisms, SnpEff: SNPs in the genome of Drosophila melanogaster strain w^{1118} ; iso-2; iso-3. Fly. 2012;6: 80–92. doi:10.4161/fly.19695
