# Supplementary material for: Population genomics of Mesolithic Scandinavia: Investigating early postglacial migration routes and high-latitude adaptation
Source: PLoS Biol. 2018 Jan 9;16(1):e2003703. doi: 10.1371/journal.pbio.2003703 (PMC5760011; doi:10.1371/journal.pbio.2003703)
Supplement: S4 Text — (PDF) [file pbio.2003703.s009.pdf]

S4 Estimates of contamination

S4.1 Mitochondrial contamination

In order to estimate mtDNA contamination we used private or near-private consensus alleles [1] (<5% in 311 modern mtDNAs) with a base and mapping quality of 30 or higher as well as minimum 10x coverage for the ancient samples. To compensate for post-mortem damage, we filtered away positions with a consensus allele of either C or G, and where a transition substitution was detected. To obtain an estimate, the counts of consensus and alternative alleles were added together across all sites (Table S4.1).

Table S4.1 Mitochondrial contamination estimates

Sample	Point estimate [%]	Informative sites	Consensus alleles	Total alleles	Lower C.I. boundary [%]	Higher C.I boundary [%]
Hum1	0.2898550725	2	1032	1035	0	0.6173812545
Hum2	0.1531393568	2	652	653	0	0.4506258182
SBj	3.719008264	6	466	484	2.033164827	5.404851702
SF9	5.357142857	4	106	112	1.869360277	9.527349687
SF11	3.418803419	2	113	117	0.1261463881	6.711460449
SF12	0.337938306	10	36864	36989	0.2787952963	0.3970813157
Steigen	0	2	506	506	0	0.5902928365

S4.2 X chromosome contamination

Contamination in male individuals (Hum2, Steigen, SF11 and SBj) was estimated using a method that examines heterozygous sites within the X chromosome. The method was first described in Rasmussen et al. [2] and is now implemented as part of ANGSD [3]. The X chromosome contamination module of ANGSD v.0.902 was run with two steps as described in the software manual. In the first step, a binary count file was built using the command “angsd -r X:5000000-154900000 -doCounts 1 -setMinDepth 3 -setMaxDepth 100 -iCounts 1 -minMapQ 30 -minQ 30”. In the second step the contamination estimate was obtained with the command “contamination -d 3 -e 100”. In this latter step, only transversion polymorphisms were screened to avoid bias due to post-mortem DNA damage. We report contamination estimates and the confidence interval from method 1, which samples all reads from each site, thereby producing a more precise and sensitive estimate compared to method 2, which randomly samples one read per site (Table S4.2).

S4.3 Contamination in the nuclear genome

In addition to the estimates obtained from the two approaches described above, we used the *verifyBamId* tool [4], previously used by [5], to estimate nuclear contamination on ancient samples. This method estimates autosomal contamination using the 1000 Genomes reference panel, and thus provides a direct estimate of the nuclear contamination.

To estimate contamination at the sample level, we ran VerifyBamID v.1.1.2 [4] with the following command “verifyBamID -vcf <1000GenomesSitesFile> -bam <bamfile> -out <output> -verbose -ignoreRG”. The 1000 genome vcf file (ftp://ftp.1000genomes.ebi.ac.uk/vol1/ftp/release/20130502/ALL.wgs.phase3_shapeit2_mvncall_integrated_v5b.20130502.sites.vcf.gz) was further filtered to contain only transversion sites to avoid overestimation of contamination due to post-mortem damage. The program reports the contamination estimates in the FREEMIX column. It is

important to note that while this method is a powerful direct tool to estimate nuclear contamination in modern samples, its accuracy to estimate contamination in ancient low coverage samples has not been formally tested. However, the consistency and low contamination estimates obtained using the three different methods for our low coverage samples such as SBj, indicate that our estimations are robust (Table S4.1 and S4.2).

Table S4.2 Two Nuclear contamination estimates.

	ANGSD Xchr		VerifyBamID			
**Sample**	**Contam [%]**	**# SNPs used**	**Contam [%]**	**Avgr Cov at SNPs**	**#Reads used**	**#SNPs used**
Steigen	0.4	2170	0	1.33	7042446	5286678
SF9	NA	NA	0	0.7	3682899	5286678
SF11	NA	NA	10.159	0.11	592735	5286678
SBj	1.4	133	0.063	0.49	2599852	5286678
Hum2	0.63	22209	0.73	4.42	23343202	5286678
Hum1	NA	NA	0	0.82	4323981	5286678
SF12	NA	NA	0.932	19.25	101772227	5286678

References

1. Green RE, Malaspinas A-S, Krause J, Briggs AW, Johnson PLF, Uhler C, et al. A Complete Neandertal Mitochondrial Genome Sequence Determined by High-Throughput Sequencing. Cell. 2008;134: 416–426. doi:10.1016/j.cell.2008.06.021

2. Rasmussen M, Guo X, Wang Y, Lohmueller KE, Rasmussen S, Albrechtsen A, et al. An Aboriginal Australian genome reveals separate human dispersals into Asia. Science. 2011;334: 94–98. doi:10.1126/science.1211177

3. Korneliussen TS, Albrechtsen A, Nielsen R. ANGSD: Analysis of Next Generation Sequencing Data. BMC Bioinformatics. 2014;15: 356. doi:10.1186/s12859-014-0356-4

4. Jun G, Flickinger M, Hetrick KN, Romm JM, Doheny KF, Abecasis GR, et al. Detecting and estimating contamination of human DNA samples in sequencing and array-based genotype data. American Journal of Human Genetics. 2012;91: 839–848. doi:10.1016/j.ajhg.2012.09.004

5. Schiffels S, Haak W, Paajanen P, Llamas B, Popescu E, Loe L, et al. Iron Age and Anglo-Saxon genomes from East England reveal British migration history. Nature communications. 2016;7: 10408. doi:10.1038/ncomms10408
