# Supplementary material for: Population genomics of Mesolithic Scandinavia: Investigating early postglacial migration routes and high-latitude adaptation
Source: PLoS Biol. 2018 Jan 9;16(1):e2003703. doi: 10.1371/journal.pbio.2003703 (PMC5760011; doi:10.1371/journal.pbio.2003703)
Supplement: S5 Text — (PDF) [file pbio.2003703.s010.pdf]

S5 Uniparental markers

S5.1 Y chromosome analyses

We used BAM files mapped to hg19 to call all single base substitutions from Phylotree [1] (version of 09/03/2016) using samtools mpileup. We extracted sites with mapping and base quality of at least 30. We excluded insertions and deletions and sites that displayed multiple alleles. We adopted a less stringent strategy, where we kept transition sites and A>T and G>C SNPs, to recover as many haplogroup defining substitutions as possible. Therefore, we report all derived states in the hierarchal phylogeny for each individual. We also report ancestral states downstream of the most derived allele to show the certainty of the haplogroup call within that branch. Additionally, we double checked that we had no ancestral alleles upstream of the defined haplogroup that would contradict the call. We used the nomenclature of the International Society of Genetic Geneaology (ISOGG) version 11.224 (http://isogg.org). For sites not present in ISOGG, we used the definitions in the minimal reference phylogeny of Phylotree (http://www.phylotree.org/Y/tree/). We caution that the allele state at a few sites may be erroneous due to post mortem deaminations, contamination or strand misidentifications. These were mainly seen as derived alleles sporadically scattered over the phylogeny and were usually contradicted by ancestral alleles at upstream positions.

Hum2
Individual Hum2 displayed the derived state at 20 sites leading in a hierarchal order to haplogroup I2-M438 (Table S5.1). We could not determine if Hum2 follows the I2c lineage as there were no sequence data for the defining markers L596 and L597. We could, however, exclude I2a and its subhaplogroups as Hum2 was ancestral for I2a-L460 as well as for an additional 20 downstream sites.

We noted a discrepancy in ancestral and derived allele state for CTS616 in the Y-SNP marker list of ISOGG and Phylotree, where the former describe the mutation as G>C and the latter as C>G. We assume that ISOGG is correct as it was most recently updated (9 December 2016) and as it makes phylogenetic sense with our data. Four additional sites displayed derived alleles for M118 (A>T), M236 (G>C), Y40 (C>T) and M417 (G>A). These are most likely erroneous because (I) they do not fit into the I2 phylogeny indicated by the majority of the sequence data (they define A1b1b2b1, B1, R1a and R1a1a1), (II) multiple upstream sites displayed ancestral alleles, and (III) they may have been caused by post mortem damage and/or possible strand misidentifications.

Steigen
Steigen belonged to I2a1b-M423 and displayed derived alleles at 14 sites leading to this haplogroup (Table S5.2). As this individual was ancestral for L161 and L621 downstream of I2a1b we conclude that he does not belong further down in this lineage. Ancestral states at 13 additional sites within the I2 phylogeny exclude the lineages I2a2 and I2c to further corroborate the I2a1b haplogroup call.

Steigen also displayed derived alleles for M281 (C>T) and Y40 (C>T) that defines E1b1b2 and R1a. As this individual displayed ancestral states for upstream sites (five and nine such sites respectively), these haplogroups do not make phylogenetic sense. Both sites were covered by a single read and they were C>T transitions that are likely caused by post mortem deamination.

SBj
SBj shows derived allele states at six sites leading to I2-L68 (xL597) (Table S5.3). We can exclude further downstream haplogroup calls within I2a2 (five sites with ancestral alleles, including I2a2-P217)

and I2c (L597). As we had no data for the basal sites within I2a we could not determine if this individual belongs to I2 or to a subhaplogroup within I2a.

SBj displayed one additional site with a derived allele state at PF2259 (G>A) that defines G2a2b2b. This is likely erroneous and does not make phylogenetic sense as SBj also displayed ancestral alleles at three positions upstream of this haplogroup.

Haplogroup I

Three Mesolithic males in this study belong either to haplogroup I2 (Hum2 and SBj) or I2a1b Steigen). Unfortunately, there was not enough Y-chromosomal data from the SF11 male to define his haplogroup. Haplogroup I is one of the most frequently occurring lineages in pre-Neolithic samples from Europe from which both Y-chromosomal and genome-wide data have been generated. The earliest hg I individual has been found in a Gravettian context in the Paglicci cave in Italy and is dated to 34,580-31,210 BP [2]. This date is slightly older than a previous estimate of the age of the haplogroup (ca 20,000-25,000 years) [3], but more in line with newer estimates (ca 21,390-36,863 years) [4]. Haplogroups I and I2 have also been found in individuals dated to 16,000-12,830 BP from Magdalenian (Burkhardtshohle and HohleFels49), Azilian (Bichon) and Epipaleolithic (Rochedane) contexts in Germany, Switzerland and France respectively [2,5]. In line with our results, five other Mesolithic males from Sweden (Motala2, Motala3, Motala6, Motala9 and Motala12), belong to I2 lineages (I2a1, I2a1b and I2c) [2,6–8]. Three additional Mesolithic individuals from France (BerryAuBac and Chaudardes1) and Luxembourg (Loschbour) belong to I and I2a1b respectively [2,6]. Other, less prevalent, lineages found in pre-Neolithic European samples are C, F, J and R [2,5,7–12].

Haplogroup I is less common than other haplogroups in samples from the Early Neolithic and onwards. We note, however, that I lineages have been found in Early Neolithic males from Turkey (I0724 and I1096), Hungary (KO1, NE7) and Spain (Troc5) [7,8,13] as well as in Middle Neolithic to Bronze Age individuals from Germany (ESP30, ESP24, EUL57, ESP2, ESP4), Hungary (RISE254, RISE247), Italy (RISE487, RISE489, RISE486), Russia (RISE552) and Spain (Mina4, MIR5/MIR6, MIR14, MIR19, MIR21, MIR25, MIR11, ATP12-1420) [7,8,14,15].

Four post-Mesolithic males from Scandinavia also belong within haplogroup I. These are one Scandinavian Middle Neolithic individual from a Pitted Ware hunter-gatherer context (Ajv58), who most likely belong to I2a1, and three Late Neolithic to Bronze Age individuals (RISE179, RISE210, RISE175) that were defined as haplogroup I [15,16]. The I and I2 lineages found in ancient Scandinavia are more common in the Balkans and Eastern Europe today [17]. Instead another I-lineage (I1-M253) is more prevalent in present-day Scandinavia [17–20].

Table S5.1 Y-chromosomal support for haplogroup I2 in individual Hum2. All markers are found in the ISOGG and Phylotree SNP databases (http://isogg.org/tree/ ISOGG_YDNA_SNP_Index.html and http://www.phylotree.org/Y/marker_list.htm), except for markers with and asterisk (*) that are only found in Phylotree.

Haplogroup	SNP/ marker	Position (hg19)	Substitution	Observed state	No. reads	Inference
A0-T	L1085	2790726	T>C	C	3	derived
A0-T	L1130	16661010	T>G	G	3	derived
A1	V168	17947672	G>A	A	5	derived
A1	V171	4898665	C>G	G	3	derived
A1b	P108	15426248	C>T	T	3	derived
A1b	V221	7589303	G>T	T	3	derived
BT	M42	21866840	A>T	T	1	derived
BT	P97	14886273	G>T	T	2	derived
CT	M168	14813991	C>T	T	1	derived
CF	P143	14197867	G>A	A	3	derived
F	M213	15526751	T>C	C	1	derived
F	M89	21917313	C>T	T	4	derived
F	P14	17398598	C>T	T	4	derived
GHIJK	F1329	8589031	C>T	T	2	derived
IJK	M523	6753519	A>G	G	3	derived
I	M170	14847792	A>C	C	2	derived
I	M258	15023364	T>C	C	1	derived
I	U179	16354708	G>A	A	1	derived
I2	M438	16638804	A>G	G	3	derived
I2	L68	18700150	C>T	T	3	derived
I2a	L460	7879415	A>C	A	4	ancestral
I2a1a	CTS595	6874115	C>T	C	1	ancestral
I2a1a1	M26	21865821	G>A	G	3	ancestral
I2a1a2a1	L1287	21970862	G>T	G	3	ancestral
I2a1b	M423	19096091	G>A	G	1	ancestral
I2a1b2	L621	18760081	G>A	G	2	ancestral
I2a2	M436	18747493	G>C	G	4	ancestral
I2a2	P217	7628484	C>T	C	1	ancestral
I2a2a	M223	21717307	G>A	G	2	ancestral
I2a2a	U250	18888200	C>G	C	2	ancestral
I2a2a1	CTS616	6906332	G>C	G	6	ancestral
I2a2a1	CTS9183	18732197	A>G	A	2	ancestral
I-M284	CTS4544*	15707131	C>T	C	1	ancestral
I2a2a1a2a	L1229	14937828	C>A	C	5	ancestral
I2a2a1b	CTS10057	19232160	C>T	C	1	ancestral
I2a2a1b	CTS10100	19255890	G>A	G	5	ancestral
I2a2a1b1	L701	6753316	C>T	C	2	ancestral
I2a2a1b2a	L801	21763755	A>C	A	2	ancestral
I2a2a1b2a1	CTS1977	14140273	G>A	G	5	ancestral
I2a2a1b2a2	CTS6433	16889964	T>C	T	3	ancestral
I2a2b	L38	15668070	A>G	A	4	ancestral

Table S5.2 Y-chromosomal support for haplogroup I2a1b in individual Steigen. All markers are found in the ISOGG and Phylotree SNP databases (http://isogg.org/tree/ ISOGG_YDNA_SNP_Index.html and http://www.phylotree.org/Y/marker_list.htm), except for markers with and asterisk (*) that are only found in Phylotree.

Haplogroup	SNP/marker	Position (hg19)	Substitution	Observed state	No. reads	Inference
A0-T	L1155	22191266	G>C	C	1	derived
CT	M168	14813991	C>T	T	4	derived
F	M213	15526751	T>C	C	1	derived
F	M89	21917313	C>T	T	1	derived
GHIJK	F1329	8589031	C>T	T	1	derived
IJK	M522	7173143	G>A	A	1	derived
IJ	M429	14031334	T>A	A	1	derived
IJ	P126	21225770	C>G	G	1	derived
I	M170	14847792	A>C	C	2	derived
I	M258	15023364	T>C	C	1	derived
I	U179	16354708	G>A	A	1	derived
I2	M438	16638804	A>G	G	1	derived
I2a	L460	7879415	A>C	C	1	derived
I2a1b	M423	19096091	G>A	A	1	derived
I2a1b1	L161	22513718	C>T	C	3	ancestral
I2a1b2	L621	18760081	G>A	G	1	ancestral
I2a1a1	M26	21865821	G>A	G	2	ancestral
I2a1a2a1	L1287	21970862	G>T	G	1	ancestral
I2a2	M436	18747493	G>C	G	4	ancestral
I2a2a	U250	18888200	C>G	C	1	ancestral
I-M284	CTS4544*	15707131	C>T	C	1	ancestral
I2a2a1a2a	L1229	14937828	C>A	C	3	ancestral
I2a2a1b	CTS10057	19232160	C>T	C	1	ancestral
I2a2a1b	CTS10100	19255890	G>A	G	3	ancestral
I2a2a1b2	Z161	2696497	C>G	C	1	ancestral
I2a2a1b2a	L80	14640715	A>G	A	1	ancestral
I2a2a1b2a1	CTS1977	14140273	G>A	G	1	ancestral
I2c	L596	14197631	G>A	G	1	ancestral
I2c	L597	18887888	T>A	T	1	ancestral

Table S5.3 Y-chromosomal support for haplogroup I2 in individual SBj. All markers are found in the ISOGG and Phylotree SNP databases (http://isogg.org/tree/ ISOGG_YDNA_SNP_Index.html and http://www.phylotree.org/Y/marker_list.htm), except for markers with and asterisk (*) that are only found in Phylotree.

Haplogroup	SNP/ marker	Position (hg19)	Substitution	Observed state	No. reads	Inference
A0-T	L1130	16661010	T>G	G	3	derived
BT	P97	14886273	G>T	T	1	derived
GHIJK	F1329	8589031	C>T	T	1	derived
HIJK	M578	7202703	C>T	T	2	derived
I	U179	16354708	G>A	A	1	derived
I2	L68	18700150	C>T	C	1	derived
I2a1a2a1	L1287	21970862	G>T	G	1	ancestral
I2a1b2a1a	S17250	15531354	G>A	G	1	ancestral
I2a2	P217	7628484	C>T	C	1	ancestral
I2a2a	M223	21717307	G>A	G	1	ancestral
I2a2a1a2a	L1229	14937828	C>A	C	1	ancestral
I2a2a1b1	L701	6753316	C>T	C	2	ancestral
I2a2a1b2	Z161	2696497	C>G	C	1	ancestral
I2c	L597	18887888	T>A	T	4	ancestral

S5.2 Mitochondrial DNA results

Strict consensus sequences were generated using samtools' mpileup and vcfutils.pl [21,22]. A minimum base quality and mapping quality score of 30, and a coverage of at least three, were used to call the consensus sequences. Ambiguous SNPs in the consensus sequences were examined manually using samtools v1.3 and the mpileup command. The majority nucleotide (>75% of the reads) was called if the ambiguous position was covered by at least 10 reads with a minimum base quality and map quality score of 30. Haplogroups were assigned to the sequences using HaploFind [23] and PhyloTree mtDNA Build 17 [1]. The mutations are reported against the Reconstructed Sapiens Reference Sequence, RSRS [24]. The mitochondrial coverage, haplogroups, mutations supporting the called haplogroup and private mutations are reported in Table S5.4. There were a few regions where none of the consensus sequences (or only SF12) had any data after filtering. The majority of these positions are situated between the *ND1* and *CO3* genes and has previously been reported as regions that are difficult to map when working with short sequence reads [25].

SF9 belongs to U4a2 (Table S5.4). This mitochondrial genome has 46 haplogroup defining mutations (and no data for defining sites at np T4646C, T5999C, A6047G, A7521G and C8818T). SF9 also has two private mutations leading to U4a2f (A1978G and A12397G). As SF9 displays the ancestral state for the two additional defining positions for U4a2f (T1189C and G15172A), it is likely an ancestral lineage to U4a2f and we report it as U4a2.

SF11 belongs to U5a1, as previously reported [16]. This individual displayed 49 mutations and lacked data for one site leading to this haplogroup (A7521G, Table S5.4). SF11 had one private mutation, which is known to be recurrent (C16519T).

SF12 displays derived alleles at all 51 positions leading to U4a1 (Table S5.4). SF12 also has two private mutations, where one is a unique C6617T transition and the second is a recurrent G13708A mutation. Given these private mutations, it is likely that SF12 belongs to a previously unknown U4a1 sub-haplogroup.

SBj belongs to U4a1 (Table S5.4). This individual has 48 of the haplogroup defining mutations, and for the remaining three positions, there is no data available (np 4646, 5999 and 6047). Like SF12, SBj also has the private G to A mutation at np 13708A. However, SBj is ancestral at np 6617 where SF12 is derived.

Hum1 has all 50 SNPs leading to U5a1 (Table S5.4). This individual has two private mutations at positions that are known to be recurrent (G4769A and 16519T). Hum1 also has two chimeric sites with both C and T reads. These are most likely caused by deamination (C4799T and C7115T), but as the number of C reads were below the 75% SNP calling cut off (65% and 69% respectively), they were kept as Y in the consensus sequences.

Hum2 has 50 mutations leading to U5a1d (Table S5.4) but lacks the C152T transition. As this mutation defines an early branch in the phylogeny (L2'3'4'5'6), the most likely explanation for C at this site in Hum2 is that a back-mutation has occurred. Hum2 has a private mutation at a position that is known to be recurrently mutating (C16519T). Hum2 may share the G4769A transition that Hum1 had. However, Hum2 is chimeric and the amount of A reads were 62% (not above the 75% we use for SNP calling) which would mean that 38% of the reads could be due to G to A damage. Therefore Hum2 was kept as R at this particular site in the consensus sequence.

Steigen, like Hum2, has 50 mutations leading to U5a1d and lacks the C to T transition at np 152 (Table S5.4). Steigen has two private SNPs at positions that mutate recurrently (A189G and C16519T). Steigen is also chimeric for G4769A (as Hum2) and as 56% of the reads were As and 44% Gs, and was, like Hum2, kept as R in the consensus sequence.

The three newly reported Swedish Mesolithic individuals belong to U4 lineages. More specifically, SF12 from Stora Karlsö and SBj from Gotland belong to U4a1, and SF9 to U4a2 (Table S5.4, Figure S5.1). Only one other complete pre-Neolithic U4a genome, from an eastern hunter-gatherer from Karelia (I0211) contextually dated to 7,450-6,950 cal BP, has been reported [8]. This makes the Swedish Mesolithic U4 lineages the earliest thus far. However, the estimated coalescence time for the haplogroup is about 20,000 years BP [26,27]. The upstream haplogroup U and U2’3’4’7 are present in pre- and post-glacial Europe (Cioclovina1, Malta1, Paglicci108 and Rigney1) [2,28,29], making it plausible to discover more U4 haplotypes as more ancient samples are analyzed.

The three Mesolithic Norwegian samples from Hummerviksholmen and Steigen belong to U5a1 lineages. Hum1 belongs to U5a1, and Hum2 and Steigen belong to U5a1d (Table S5.4, Figure S5.1). Also the Swedish Stora Förvar sample, SF11 belongs to the same lineage as Hum1 (U5a1) [16]. In addition to our Mesolithic samples, two U5a1 haplotypes are found in Scandinavian hunter-gatherers from mainland Sweden dated to 7,900-7,500 cal BP (Motala1 and Motala3), one U5a1c has been found in a hunter-gatherer dated to 8,417-8,199 cal BP from Latvia (Latvia_HG1) [12], and one U5a1d in an eastern hunter-gatherer from Russia dated to 7,490-7,505 cal BP (Samara) [6–8] (Figure S5.1). The Norwegian Hum1 and Hum2 are the oldest representatives of U5a1 lineages found thus far. U5 is by far the most commonly found haplogroup among European Mesolithic individuals (24 of 36 individuals). U5a2 lineages are found in seven individuals from Sweden, France and Germany (Motala4, Motala6, Motala9, LesCloseaux13, MareuilLesMeaux1, Felsdach and Blätterhöhle20) and U5b lineages are found in 11 individuals from Spain, France, Luxembourg, Germany, Italy and Ukraine (LaBrana1, Ranchot88, BerryAuBac, Chaudardes1, Loschbour, Bockstein, Ofnet, Falkenstein and HohlensteinStadel, Continenza, Ukraine_HG1) [2,6–8,11,12,28,30,31] (Figure S5.1). U5 is thought to have arisen in Upper Paleolithic Europe and molecular dating of modern mitochondrial genomes estimates the age of the haplogroup to ca. 25,000-36,000 years [26,32–34]. This is in line with recent findings of six pre-LGM individuals dated to between 31,000 and 26,000 BP carrying U5 haplotypes (KremsWA3, Vestonice15, Pavlov1, Vestonice16, Vestonice43 and Goyet2878-21) [2,28,35] as well as with U5b lineages found in individuals from Spain, Italy, France, Germany and Switzerland dated to 19,000-12,000 BP (ElMiron, Paglicci71, Villabruna, Iboussieres31-2, Iboussieres25-1, Iboussieres39, Rochedane, Oberkassel and Bichon) [2,5,28]. Except for the U4 and U5 lineages, there are also a few less common haplogroups (and subtypes thereof) in individuals from Mesolithic Europe. These are U2e lineages found in Sweden (Motala2 and Motala12) and Latvia (Latvia_HG2), C1 in Russia (Karelia), H13c in Georgia (Kotias) and R3 in Hungary (KO1, which is Neolithic but has been shown to be part of the Mesolithic gene pool) [6–8,12,13,36] (Figure S5.1).

Table S5.4 Mitochondrial coverage, haplogroup assignment, polymorphisms supporting the assigned haplogroup and private mutations in the Swedish and Norwegian Mesolithic consensus sequences.

Sample	MT coverage	MT hg	Polymorphisms for called hg (against RSRS)	Private mutations
SF9	93	U4a2	146T 152T 247G 310C 499A 769G 825T 1018G 1811G 2758G 2885T 3594C 4104A 4312C 7146A 7256C 7521G 8468C 8655C 8701A 9540T 10398A 10664C 10688G 10810T 10873T 10915T 11332T 11467G 11914G 12308G 12372A 12705C 13105A 13276A 13506C 13650C 14620T 15693C 16129G 16187C 16189T 16223C 16230A 16278C 16311T 16356C	1978G 12397G
SF11	45.	U5a1	146T 152T 195T 247G 769G 825T 1018G 2758G 2885T 3197C 3594C 4104A 4312C 7146A 7256C 8468C 8655C 8701A 9477A 9540T 10398A 10664C 10688G 10810T 10873T 10915T 11467G 11914G 12308G 12372A 12705C 13105A 13276A 13506C 13617C 13650C 14793G 15218G 16129G 16187C 16189T 16192T 16223C 16230A 16256T 16270T 16278C 16311T 16399G	16519T
SF12	9774	U4a1	146T 247G 499A 769G 825T 1018G 1811G 2758G 2885T 3594C 4104A 4312C 4646C 5999C 6047G 7146A 7256C 7521G 8468C 8655C 8701A 8818T 9540T 10398A 10664C 10688G 10810T 10873T 10915T 11332T 11467G 11914G 12308G 12372A 12705C 12937G 13105A 13276A 13506C 13650C 14620T 15693C 16129G 16134T 16187C 16189T 16223C 16230A 16278C 16311T 16356C	6617T 13708A
SBj	102	U4a1	146T 247G 499A 769G 825T 1018G 1811G 2758G 2885T 3594C 4104A 4312C 7146A 7256C 7521G 8468C 8655C 8701A 8818T 9540T 10398A 10664C 10688G 10810T 10873T 10915T 11332T 11467G 11914G 12308G 12372A 12705C 12937G 13105A 13276A 13506C 13650C 14620T 15693C 16129G 16134T 16187C 16189T 16223C 16230A 16278C 16311T 16356C	13708A
Hum1	597	U5a1	146T 152T 195T 247G 769G 825T 1018G 2758G 2885T 3197C 3594C 4104A 4312C 7146A 7256C 7521G 8468C 8655C 8701A 9477A 9540T 10398A 10664C 10688G 10810T 10873T 10915T 11467G 11914G 12308G 12372A 12705C 13105A 13276A 13506C 13617C 13650C 14793G 15218G 16129G 16187C 16189T 16192T 16223C 16230A 16256T 16270T 16278C 16311T 16399G	4769A 16519T
Hum2	432	U5a1d	146T 195T 247G 769G 825T 1018G 2758G 2885T 3027C 3197C 3594C 4104A 4312C 7146A 7256C 7521G 8468C 8655C 8701A 9477A 9540T 10398A 10664C 10688G 10810T 10873T 10915T 11467G 11914G 12308G 12372A 12705C 13105A 13276A 13506C 13617C 13650C 14793G 15218G 16129G 16187C 16189T 16192T 16223C 16230A 16256T 16270T 16278C 16311T 16399G	16519T
Steigen	278	U5a1d	146T 195T 247G 769G 825T 1018G 2758G 2885T 3027C 3197C 3594C 4104A 4312C 7146A 7256C 7521G 8468C 8655C 8701A 9477A 9540T 10398A 10664C 10688G 10810T 10873T 10915T 11467G 11914G 12308G 12372A 12705C 13105A 13276A 13506C 13617C 13650C 14793G 15218G 16129G 16187C 16189T 16192T 16223C 16230A 16256T 16270T 16278C 16311T 16399G	189G 16519T

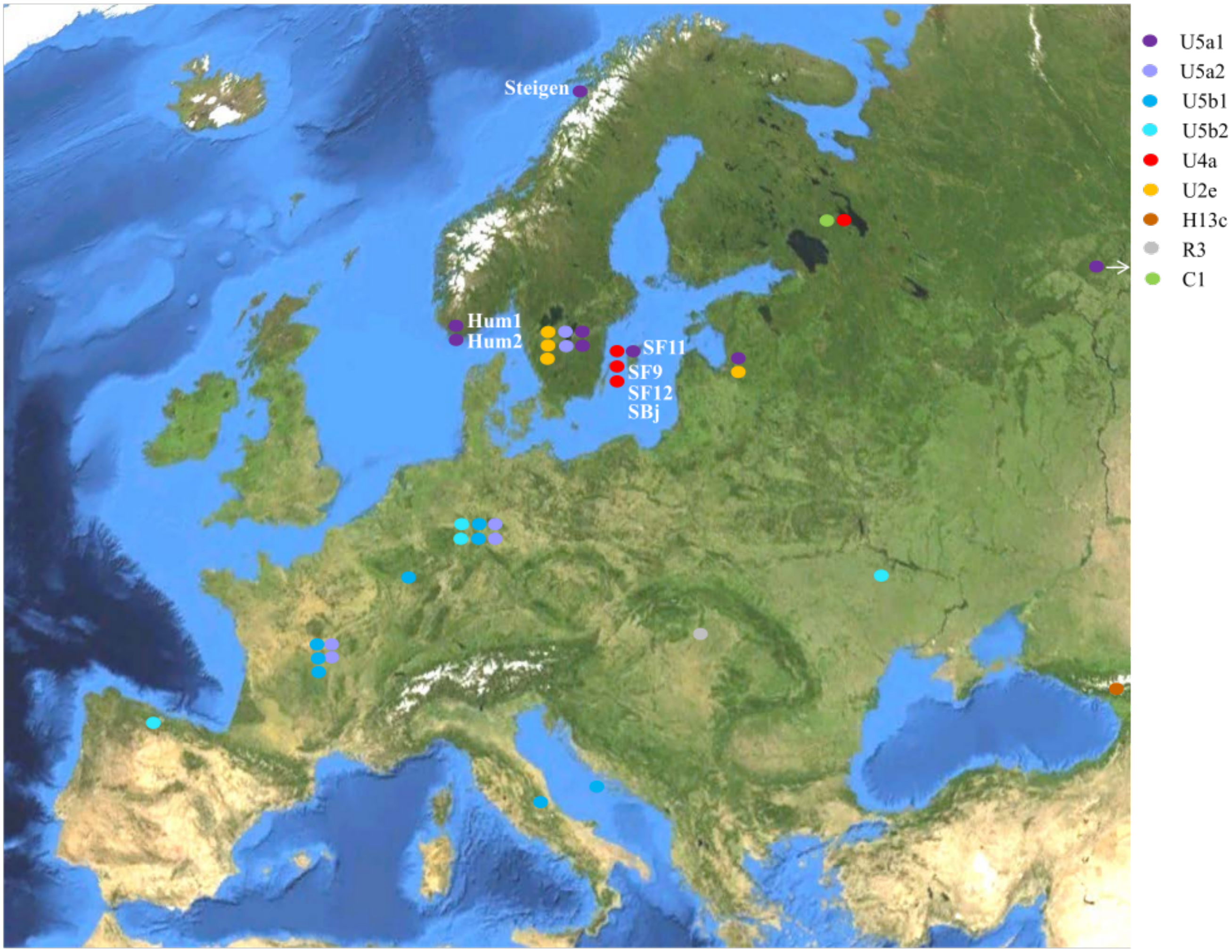

Figure S5.1 36 complete mitochondrial genomes from Mesolithic Europe including the three Norwegian samples Steigen, Hum1, Hum2, and the four Swedish samples SF9, SF11, SF12 and SBj. Seven previously published samples from Sweden [6–8], two from Latvia [12], one from Spain [11,31], five from France [2,28], one from Luxembourg [6], six from Germany [2,28,30], one from Italy [2], one from Hungary [13], one from Georgia [5], one from Ukraine [12] and three from Russia [6–8,36]. The underlying map can be found at https://commons.wikimedia.org/wiki/Atlas_of_Europe

References

1. van Oven M. PhyloTree Build 17: Growing the human mitochondrial DNA tree. Forensic Science International: Genetics Supplement Series. 2015;5: 9–11. doi:10.1016/j.fsigss.2015.09.155

2. Fu Q, Posth C, Hajdinjak M, Petr M, Mallick S, Fernandes D, et al. The genetic history of Ice Age Europe. Nature. 2016;534: 200–5. doi:10.1038/nature17993

3. Semino O, Passarino G, Oefner PJ, Lin AA, Arbuzova S, Beckman LE, et al. The genetic legacy of paleolithic Homo sapiens sapiens in extant europeans: A Y chromosome perspective. Science. 2000;290: 1155–1159. doi:10.1126/science.290.5494.1155

4. Karmin M, Saag L, Vicente M, Wilson Sayres MA, Järve M, Talas UG, et al. A recent bottleneck of Y chromosome diversity coincides with a global change in culture. Genome Research. 2015;25: 459–466. doi:10.1101/gr.186684.114

5. Jones ER, Gonzalez-Fortes G, Connell S, Siska V, Eriksson A, Martiniano R, et al. Upper Palaeolithic genomes reveal deep roots of modern Eurasians. Nature communications. 2015;6: 8912. doi:10.1038/ncomms9912

6. Lazaridis I, Patterson N, Mittnik A, Renaud G, Mallick S, Kirsanow K, et al. Ancient human genomes suggest three ancestral populations for present-day Europeans. Nature. 2014;513: 409–413. doi:10.1038/nature13673

7. Haak W, Lazaridis I, Patterson N, Rohland N, Mallick S, Llamas B, et al. Massive migration from the steppe was a source for Indo-European languages in Europe. Nature. 2015; doi:10.1038/nature14317

8. Mathieson I, Lazaridis I, Rohland N, Mallick S, Patterson N, Roodenberg SA, et al. Genome-wide patterns of selection in 230 ancient Eurasians. Nature. 2015;528: 499–503. doi:10.1038/nature16152

9. Fu Q, Hajdinjak M, Moldovan OT, Constantin S, Mallick S, Skoglund P, et al. An early modern human from Romania with a recent Neanderthal ancestor. Nature. 2015;524: 216–9. doi:10.1038/nature14558

10. Seguin-Orlando A, Korneliussen TS, Sikora M, Malaspinas A-S, Manica A, Moltke I, et al. Genomic structure in Europeans dating back at least 36,200 years. Science. 2014;346: 1113–1118. doi:10.1126/science.aaa0114

11. Olalde I, Allentoft ME, Sánchez-Quinto F, Santpere G, Chiang CWK, DeGiorgio M, et al. Derived immune and ancestral pigmentation alleles in a 7,000-year-old Mesolithic European. Nature. 2014;507: 225–228. doi:10.1038/nature12960

12. Jones ER, Zarina G, Moiseyev V, Lightfoot E, Nigst PR, Manica A, et al. The Neolithic Transition in the Baltic Was Not Driven by Admixture with Early European Farmers. Curr Biol. 2017;27: 576–582. doi:10.1016/j.cub.2016.12.060

13. Gamba C, Jones ER, Teasdale MD, McLaughlin RL, Gonzalez-Fortes G, Mattiangeli V, et al. Genome flux and stasis in a five millennium transect of European prehistory. Nature Communications. 2014;5: 5257. doi:10.1038/ncomms6257

14. Günther T, Valdiosera C, Malmström H, Ureña I, Rodriguez-Varela R, Sverrisdóttir ÓO, et al. Ancient genomes link early farmers from Atapuerca in Spain to modern-day Basques. Proc Natl Acad Sci USA. 2015;112: 11917–11922. doi:10.1073/pnas.1509851112

15. Allentoft ME, Sikora M, Sjögren K-G, Rasmussen S, Rasmussen M, Stenderup J, et al. Population genomics of Bronze Age Eurasia. Nature. 2015;522: 167–172. doi:10.1038/nature14507

16. Skoglund P, Malmstrom H, Omrak A, Raghavan M, Valdiosera C, Gunther T, et al. Genomic Diversity and Admixture Differs for Stone-Age Scandinavian Foragers and Farmers. Science. 2014;344: 747–750. doi:10.1126/science.1253448

17. Rootsi S, Kivisild T, Benuzzi G, Help H, Bermisheva M, Kutuev I, et al. Phylogeography of Y-Chromosome Haplogroup I Reveals Distinct Domains of Prehistoric Gene Flow in Europe. The American Journal of Human Genetics. 2004;75: 128–137. doi:10.1086/422196

18. Karlsson AO, Wallerström T, Götherström A, Holmlund G. Y-chromosome diversity in Sweden - a long-time perspective. European journal of human genetics : EJHG. 2006;14: 963–70. doi:10.1038/sj.ejhg.5201651

19. Passarino G, Cavalleri GL, Lin A a, Cavalli-Sforza LL, Børresen-Dale A-L, Underhill P a. Different genetic components in the Norwegian population revealed by the analysis of mtDNA and Y chromosome polymorphisms. European journal of human genetics : EJHG. 2002;10: 521–529. doi:10.1038/sj.ejhg.5200834

20. Lappalainen T, Laitinen V, Salmela E, Andersen P, Huoponen K, Savontaus ML, et al. Migration waves to the baltic sea region. Annals of Human Genetics. 2008;72: 337–348. doi:10.1111/j.1469-1809.2007.00429.x

21. Li H, Handsaker B, Wysoker A, Fennell T, Ruan J, Homer N, et al. The Sequence Alignment/Map format and SAMtools. Bioinformatics. 2009;25: 2078–2079. doi:10.1093/bioinformatics/btp352

22. Danecek P, Auton A, Abecasis G, Albers CA, Banks E, DePristo MA, et al. The variant call format and VCFtools. Bioinformatics. 2011;27: 2156–2158. doi:10.1093/bioinformatics/btr330

23. Vianello D, Sevini F, Castellani G, Lomartire L, Capri M, Franceschi C. HAPLOFIND: A New Method for High-Throughput mtDNA Haplogroup Assignment. Human Mutation. 2013;34: 1189–1194. doi:10.1002/humu.22356

24. Behar DM, van Oven M, Rosset S, Metspalu M, Loogväli E-L, Silva NM, et al. A "Copernican" reassessment of the human mitochondrial DNA tree from its root. Am J Hum Genet. 2012;90: 675–684. doi:10.1016/j.ajhg.2012.03.002

25. Marinov GK, Wang YE, Chan D, Wold BJ. Evidence for site-specific occupancy of the mitochondrial genome by nuclear transcription factors. PLoS ONE. 2014;9. doi:10.1371/journal.pone.0084713

26. Richards M, Macaulay V, Hickey E, Vega E, Sykes B, Guida V, et al. Tracing European founder lineages in the Near Eastern mtDNA pool. Am J Hum Genet. 2000;67: 1251–1276.

27. Malyarchuk B, Grzybowski T, Derenko M, Perkova M, Vanecek T, Lazur J, et al. Mitochondrial DNA phylogeny in eastern and western Slavs. Molecular Biology and Evolution. 2008;25: 1651–1658. doi:10.1093/molbev/msn114

28. Posth C, Renaud G, Mittnik A, Drucker DG, Rougier H, Cupillard C, et al. Pleistocene mitochondrial genomes suggest a single major dispersal of non-africans and a late glacial population turnover in Europe. Current Biology. 2016;26: 827–833. doi:10.1016/j.cub.2016.01.037

29. Raghavan M, Skoglund P, Graf KE, Metspalu M, Albrechtsen A, Moltke I, et al. Upper Palaeolithic Siberian genome reveals dual ancestry of Native Americans. Nature. 2014;505: 87–91. doi:10.1038/nature12736

30. Bollongino R, Nehlich O, Richards MP, Orschiedt J, Thomas MG, Sell C, et al. 2000 Years of Parallel Societies in Stone Age Central Europe. Science. 2013;342: 479–481. doi:10.1126/science.1245049

31. Sánchez-Quinto F, Schroeder H, Ramirez O, Ávila-Arcos MC, Pybus M, Olalde I, et al. Genomic Affinities of Two 7,000-Year-Old Iberian Hunter-Gatherers. Current Biology. 2012;22: 1494–1499. doi:10.1016/j.cub.2012.06.005

32. Richards MB, Macaulay VA, Bandelt HJ, Sykes BC. Phylogeography of mitochondrial DNA in western Europe. Ann Hum Genet. 1998;62: 241–260. doi:10.1046/j.1469-1809.1998.6230241.x

33. Soares P, Ermini L, Thomson N, Mormina M, Rito T, Röhl A, et al. Correcting for Purifying Selection: An Improved Human Mitochondrial Molecular Clock. American Journal of Human Genetics. 2009;84: 740–759. doi:10.1016/j.ajhg.2009.05.001

34. Malyarchuk B, Derenko M, Grzybowski T, Perkova M, Rogalla U, Vanecek T, et al. The Peopling of Europe from the Mitochondrial Haplogroup U5 Perspective. Gilbert MTP, editor. PLoS ONE. 2010;5: e10285. doi:10.1371/journal.pone.0010285

35. Fu Q, Mittnik A, Johnson PLF, Bos K, Lari M, Bollongino R, et al. A Revised Timescale for Human Evolution Based on Ancient Mitochondrial Genomes. Current Biology. 2013;23: 553–559. doi:10.1016/j.cub.2013.02.044

36. Der Sarkissian C, Brotherton P, Balanovsky O, Templeton JEL, Llamas B, Soubrier J, et al. Mitochondrial genome sequencing in mesolithic North East Europe unearths a new sub-clade within the broadly distributed human haplogroup C1. PLoS ONE. 2014;9. doi:10.1371/journal.pone.0087612
