# Supplementary material for: Population genomics of Mesolithic Scandinavia: Investigating early postglacial migration routes and high-latitude adaptation
Source: PLoS Biol. 2018 Jan 9;16(1):e2003703. doi: 10.1371/journal.pbio.2003703 (PMC5760011; doi:10.1371/journal.pbio.2003703)
Supplement: S6 Text — (PDF) [file pbio.2003703.s011.pdf]

S6 Basic Population genomic analysis

S6.1 Reference data for comparison

Most population genomic analyses require a set of reference data for comparison. We compiled three different data sets from the literature and merged them with the data from ancient individuals. The three reference SNP panels were (Table S6.1).

Table S6.1 Merged SNPs with genotype datasets

Individual	Number of SNPs overlapping with Human Origins	Number of SNPs overlapping with Mathieson2015 capture SNPs	Number of SNPs overlapping with 1000 genomes transversions	Reference
Bichon	102540	299715	1694024	Jones et al. (2015)
Kostenki14	65064	185278	954417	Seguin-Orlando et al. (2014)
LaBrana1	544119	958583	1435555	Olalde et al. (2014)
KO1	414976	745223	1039570	Gamba et al. (2014)
MA1	35313	99763	504287	Raghavan et al. (2014)
Mota	102604	300381	1699427	Gallego-Llorente2015
NE5	74916	203483	858144	Gamba et al. (2014)
NE6	73159	198106	974979	Gamba et al. (2014)
NE7	74626	202923	949847	Gamba et al. (2014)
Satsurblia	75549	221996	1158984	Jones et al. (2015)
Motala1/I0011	347021	587972	160770	Lazaridis et al. (2014), Mathieson et al. (2015)
Motala2/I0012	434301	731445	234654	Lazaridis et al. (2014), Mathieson et al. (2015)
Motala3/I0013	258476	298714	-*	Lazaridis et al. (2014), Mathieson et al. (2015)
Motala4/I0014	434714	698031	188548	Lazaridis et al. (2014), Mathieson et al. (2015)
Motala6/I0015	370945	556799	127750	Lazaridis et al. (2014), Mathieson et al. (2015)
Motala12/I0017	503322	857690	1457992	Lazaridis et al. (2014), Mathieson et al. (2015)
UzOO74/I0061	536949	957887	390487	Mathieson et al. (2015)
SVP44/I0124	282751	446671	99887	Mathieson et al. (2015)
UzOO40/I0211	73963	135875	25445	Mathieson et al. (2015)
BAR2/I0707	536086	941835	442887	Mathieson et al. (2015)
BAR6/I0708	525535	913818	389237	Mathieson et al. (2015)
BAR20/I0709	531783	920496	436761	Mathieson et al. (2015)
L11-216/I0736	462639	755679	218371	Mathieson et al. (2015)
M10-275/I0744	477950	822903	260522	Mathieson et al. (2015)
M11-363/I0745	533156	927837	464096	Mathieson et al. (2015)
L11-322/I0746	535594	935324	446015	Mathieson et al. (2015)
BAR26/I1096	390684	705558	147125	Mathieson et al. (2015)
BAR271/I1097	388345	702390	145044	Mathieson et al. (2015)
BAR99/I1098	409836	740392	159847	Mathieson et al. (2015)
M11-352a/I1101	339277	616629	122322	Mathieson et al. (2015)
M11-S-350/I1103	309136	561561	103912	Mathieson et al. (2015)

M13-72/I1579	423543	772168	200584	Mathieson et al. (2015)
L12-393/I1580	471099	855638	311850	Mathieson et al. (2015)
L12-502/I1581	425080	774697	213779	Mathieson et al. (2015)
L14-200/I1583	505043	914076	389573	Mathieson et al. (2015)
M11-59/I1585	427645	778039	232951	Mathieson et al. (2015)
LBK1992/I0025	476557	827905	244202	Mathieson et al. (2015)
LBK2155/I0026	485096	850232	234029	Mathieson et al. (2015)
HAL5/I0046	464381	829856	351506	Mathieson et al. (2015)
UWS4/I0054	547797	960468	626530	Mathieson et al. (2015)
HAL4/I0100	531901	922902	410387	Mathieson et al. (2015)
CB13	63867	183119	970351	Olalde et al. (2016)
Gok2	70093	187298	888578	Skoglund et al. (2014)
Hum1	36545	107443	562138	this study
Hum2	97637	286491	1603656	this study
SBj	30512	87942	574454	this study
SF11	11886	33728	171620	Skoglund et al. (2014) and this study
SF9	51038	146258	731468	this study
Steigen	76113	224241	1217162	this study
Ajv58	96273	281130	1531643	Skoglund et al. (2014) and this study
Ajv70	79856	228312	1171958	Skoglund et al. (2014) and this study
Kotias	97602	289363	1521960	Jones et al. (2015)
NE1	97147	1095486	1562267	Gamba et al. (2014)
LBK/Stuttgart	554264	290309	1611413	Lazaridis et al. (2014)
Loschbour	558885	1058564	1571764	Lazaridis et al. (2014)
Ust_Ishim	573301	1069826	1661490	Fu et al. (2014)
SF12	576797	1101606	1749144	this study
Zv313# (Latvia_HG1)	67736	241991	1017742	Jones et al. (2017)
Zv93# (Latvia_HG2)	98071	349433	1535533	Jones et al. (2017)
Zv121# (Latvia_HG3)	56402	200712	835147	Jones et al. (2017)

*Motala3 was excluded from the analysis of the shotgun BAM files as the BAM file seems identical to Motala12.

#The individuals from Jones et al. (2017) were named based on the burial number at the site. The original names used by Jones et al. (2017) are given in parentheses.

S6.2 Principal component analysis

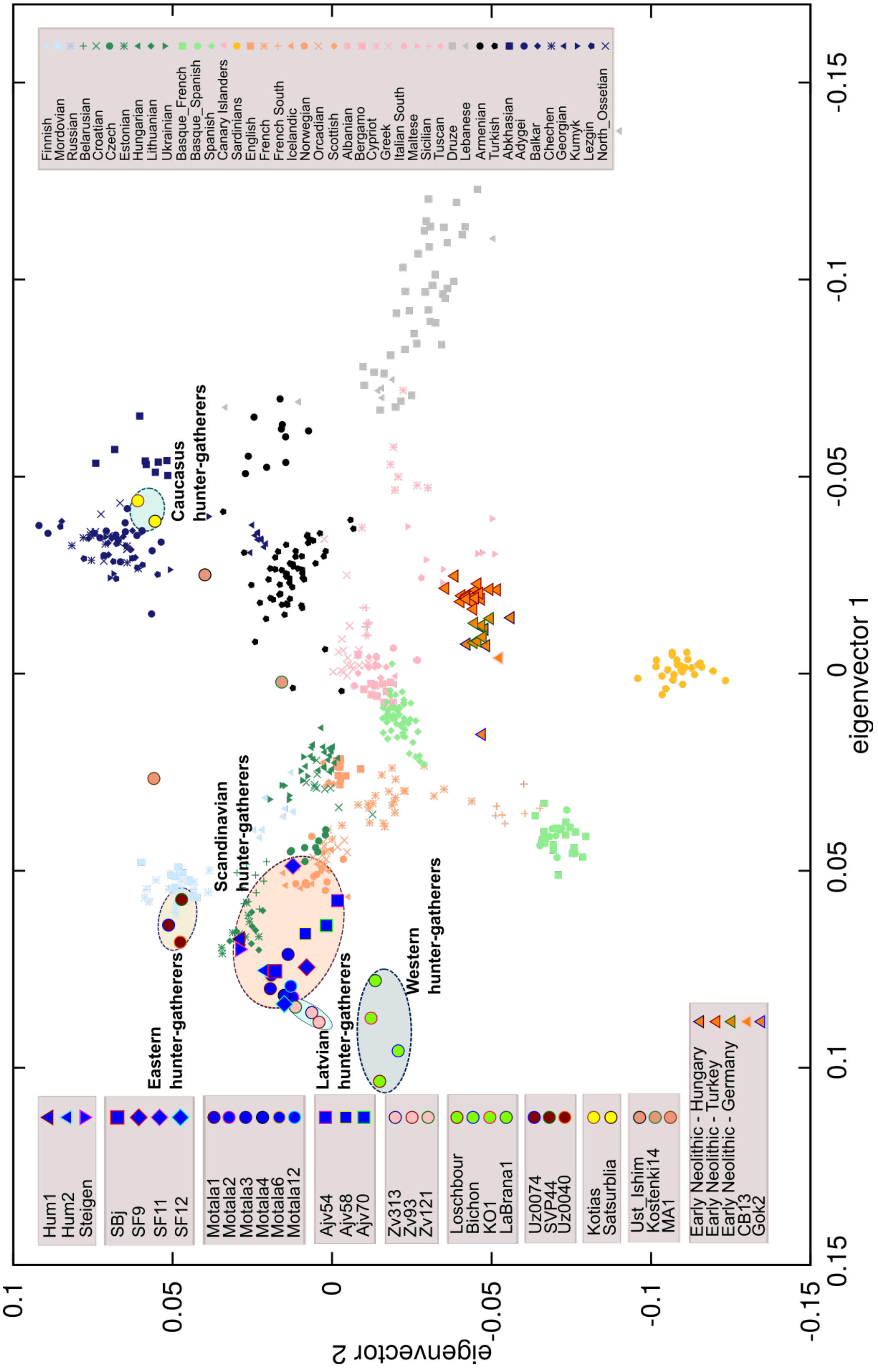

Figure S6.1 PCA of ancient individuals used in this study projected on a PCA of modern-day western Eurasians.

All Mesolithic individuals cluster tightly with individuals from a similar geographic region and cultural context (Figure 1 and Figure S6.1). The only exception appears to be SF11, instead of clustering with other Mesolithic individuals from Gotland, it clusters with Neolithic Pitted Ware culture (PWC) samples (also from Gotland). This grouping is consistent with a previous publication involving these individuals [1]. SF11 is also the lowest coverage individual with the highest estimate of nuclear contamination (Table 1, Table S4.2). To further investigate the clustering of SF11, we used SF9 as a representative of Mesolithic Gotland and down-sampled it to 34000, 19000, 15000, 8000 and 4000 SNPs. Additionally we produced ten different subsamples of SF9 with 10000 SNPs each to represent the noise caused by this low number of SNPs. While this subsampling added noise to the position of SF9 in the PCA, it was not possible to reproduce the position of SF11 (Figure S6.2). To assess whether modern contamination could have caused the clustering of SF11, we restricted the analysis to damaged reads using PMDTools [2] and PMD score cutoffs of 2 and 3. This filtering step changed the position of SF11 in the PCA but did not lead to a clustering with other Mesolithic samples from Gotland (here represented by SF9). Finally, we also tried to combine contamination and low coverage by artificially adding 10 and 20% nuclear contamination from a randomly chosen GBR sample from the 1000 genomes data [3] to the subsampled (8000 SNPs) SF9 sample. Adding contamination to SF9 results in a position closer to SF11's location in the PCA, but even 20% contamination is not sufficient to reproduce the clustering of SF11. We note that contamination from a source other than modern North-Western Europeans might cause other patterns. This led us to the conclusion that the position of SF11 in the PCA might be a combination of noise due to lower coverage, modern contamination, and possibly a slightly different genetic makeup of SF11.

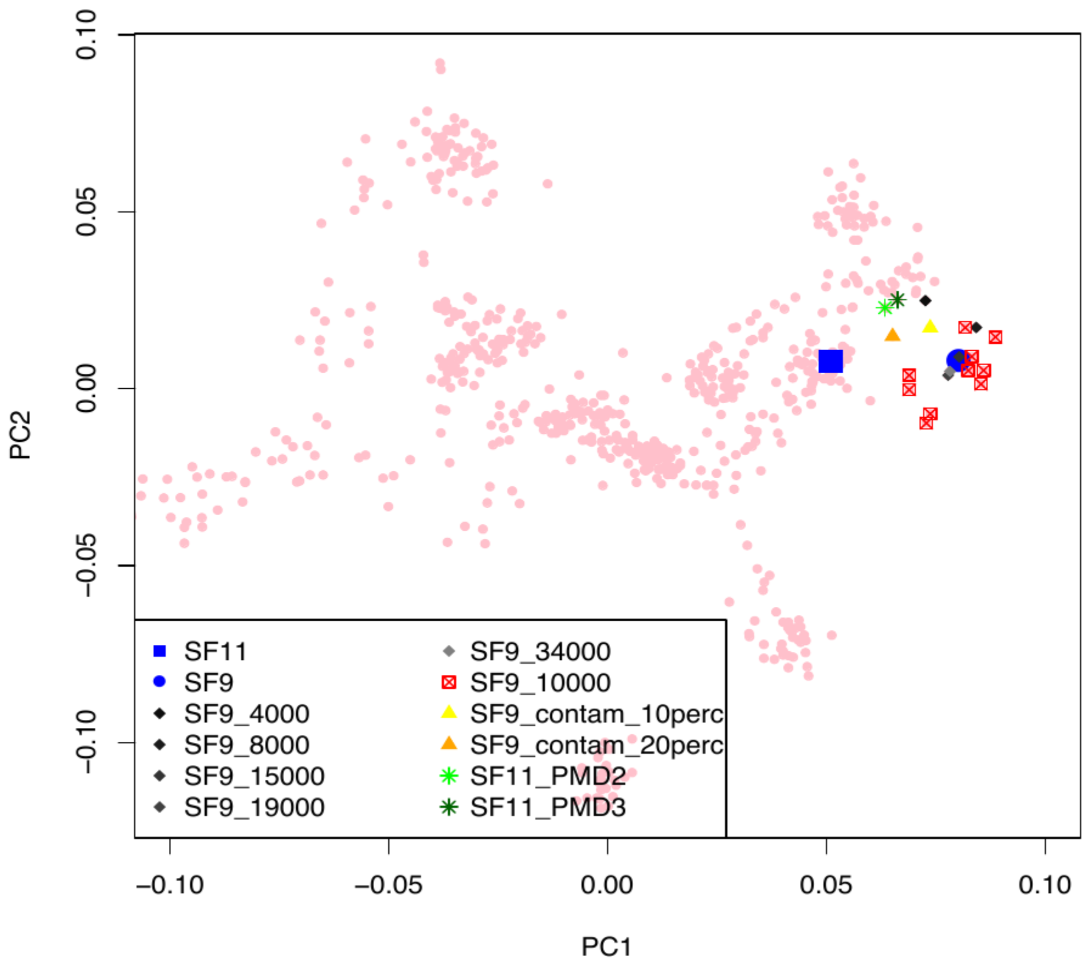

Figure S6.2 PCA for SF11 as well as different subsets and simulated contamination of SF9.

S6.3 Admixture graphs

Relationships amongst ancient individuals were examined using a statistical framework as implemented in TreeMix [4]. Treemix builds a maximum likelihood tree of populations using the covariance matrix of allele frequencies and fits admixture edges to given populations. TreeMix was applied to SF12, Motala12 [5] (as representatives of Scandinavian hunter-gatherers from Sweden), Hum1, Hum2 (as representatives of Scandinavian hunter-gatherers from Norway), Loschbour [5] (as representative of west European hunter-gatherers), and UzOO74 [6] (I0061, as representative of east European hunter-gatherers) and the tree was rooted with the ancient Ethiopian Mota individual [7]. The dataset was haploidized by selecting one random allele per each allele in each individual. Since single individuals were used separately, TreeMix was run with “-noss” option to turn off the correction for low sample size and standard errors were estimated using blocks of 500 SNPs with (-k 500 -se) parameters.

Analysis was restricted with a total of 73,960 overlapping transversion SNPs that were ascertained in African Yoruba individuals from the 1000 Genomes project [3] and were genotyped in seven ancient individuals. TreeMix was run by modelling “known” gene flow from east European hunter-gatherers

into all four Scandinavian hunter-gatherers using the (–cor-mig and –climb) parameters with 50 different random seeds. Starting proportion of admixture was set as 0.0 to allow 0% possibility of gene flow. The majority of runs (78%) supported the gene flow from EHGs to all SHGs. Hum1 and Hum2 received more EHG ancestry with admixture proportions of 48% and 45%, respectively while SF12 and Motala12 received approximately 12% EHG ancestry ($p < 0.05$). We report the graph with the highest likelihood and most common topology in Figure S6.3.

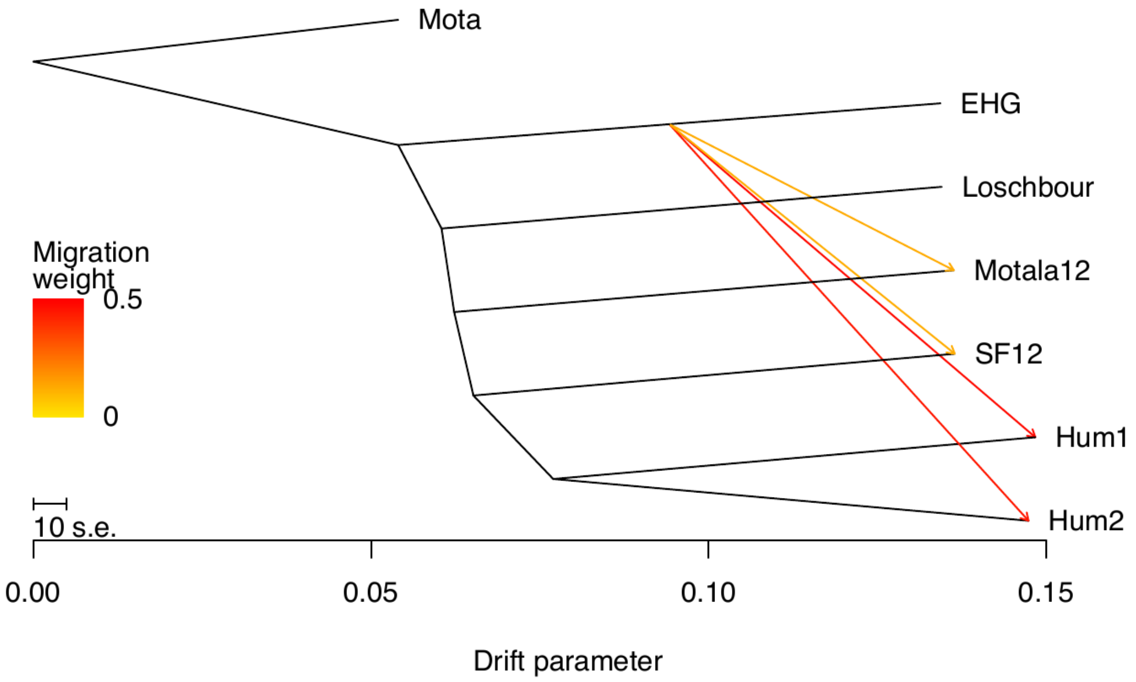

Figure S6.3 Treemix model of the data including gene flow from EHG into all SHGs.

References

1. Skoglund P, Malmstrom H, Omrak A, Raghavan M, Valdiosera C, Gunther T, et al. Genomic Diversity and Admixture Differs for Stone-Age Scandinavian Foragers and Farmers. Science. 2014;344: 747–750. doi:10.1126/science.1253448

2. Skoglund P, Northoff BH, Shunkov MV, Derevianko AP, Pääbo S, Krause J, et al. Separating endogenous ancient DNA from modern day contamination in a Siberian Neandertal. Proceedings of the National Academy of Sciences. 2014;111: 2229–2234.

3. Auton A, Abecasis GR, Altshuler DM, Durbin RM, Abecasis GR, Bentley DR, et al. A global reference for human genetic variation. Nature. 2015;526: 68–74. doi:10.1038/nature15393

4. Pickrell JK, Pritchard JK. Inference of population splits and mixtures from genome-wide allele frequency data. PLoS Genet. 2012;8: e1002967. doi:10.1371/journal.pgen.1002967

5. Lazaridis I, Patterson N, Mittnik A, Renaud G, Mallick S, Kirsanow K, et al. Ancient human genomes suggest three ancestral populations for present-day Europeans. Nature. 2014;513: 409–413. doi:10.1038/nature13673

6. Mathieson I, Lazaridis I, Rohland N, Mallick S, Patterson N, Roodenberg SA, et al. Genome-wide patterns of selection in 230 ancient Eurasians. Nature. 2015;528: 499–503. doi:10.1038/nature16152

7. Llorente MG, Jones ER, Eriksson A, Siska V, Arthur KW, Arthur JW, et al. Ancient Ethiopian genome reveals extensive Eurasian admixture throughout the African continent. Science. 2015;350: 820–2. doi:10.1126/science.aad2879
