# Supplementary material for: Population genomics of Mesolithic Scandinavia: Investigating early postglacial migration routes and high-latitude adaptation
Source: PLoS Biol. 2018 Jan 9;16(1):e2003703. doi: 10.1371/journal.pbio.2003703 (PMC5760011; doi:10.1371/journal.pbio.2003703)
Supplement: S7 Text — (PDF) [file pbio.2003703.s012.pdf]

S7 Diversity estimates

S7.1 Pairwise mismatch

Conditional nucleotide diversity [1] is a simple diversity measure which should be approximately equivalent to the heterozygosity within a population. It is based on pairwise mismatches between two haploid ancient individuals combined into one pseudo-diploid individual. Only sites known to be polymorphic are used for this comparison, the conditional nucleotide diversity is the number of pairwise mismatches divided by the number of overlapping sites covered in both individuals. To avoid effects of post-mortem damage and ascertainment bias, we only calculated this estimate for 1,797,398 transversion SNPs ascertained in Yorubans [2]. Standard errors were estimated using a block jackknife procedure with a blocksize of 2000 SNPs.

First, diversities were calculated for a mixed data set of shotgun sequence data and SNP capture data. For the individuals from Motala, both shotgun sequence [3] and SNP capture data [4,5] was available. We noticed, however, that diversities for SNP capture from Motala (e.g. Motala12-Motala1: 0.22) were substantially higher than diversities estimated from shotgun sequence data of the same individuals (0.1997). The values obtained from SNP capture data would reach similar levels as shotgun-sequenced Neolithic individuals (e.g. NE5-NE6: 0.22). As it seems well established that Neolithic populations had a higher diversity than Mesolithic Europeans [1,3,6,7], we conclude that some technical bias in the comparison of capture data and shotgun-sequence data likely causes these inflated diversity estimates, which is why we restrict all diversity estimates in this paper to shotgun-data. A plausible explanation could be that the targeted capture of the alternative allele could cause less reference bias than shotgun sequencing. However, the differences between shotgun and SNP capture data are not expected to affect our results for PCA, Admixture, Treemix or f-tests in any way. Genotyping error or reference bias should just be observed as an excess of sample-specific drift without specific direction in those analyses. We note however, that diversity in SNP capture EHGs appeared to be higher (~0.24) than in SNP capture Motala data (~0.22).

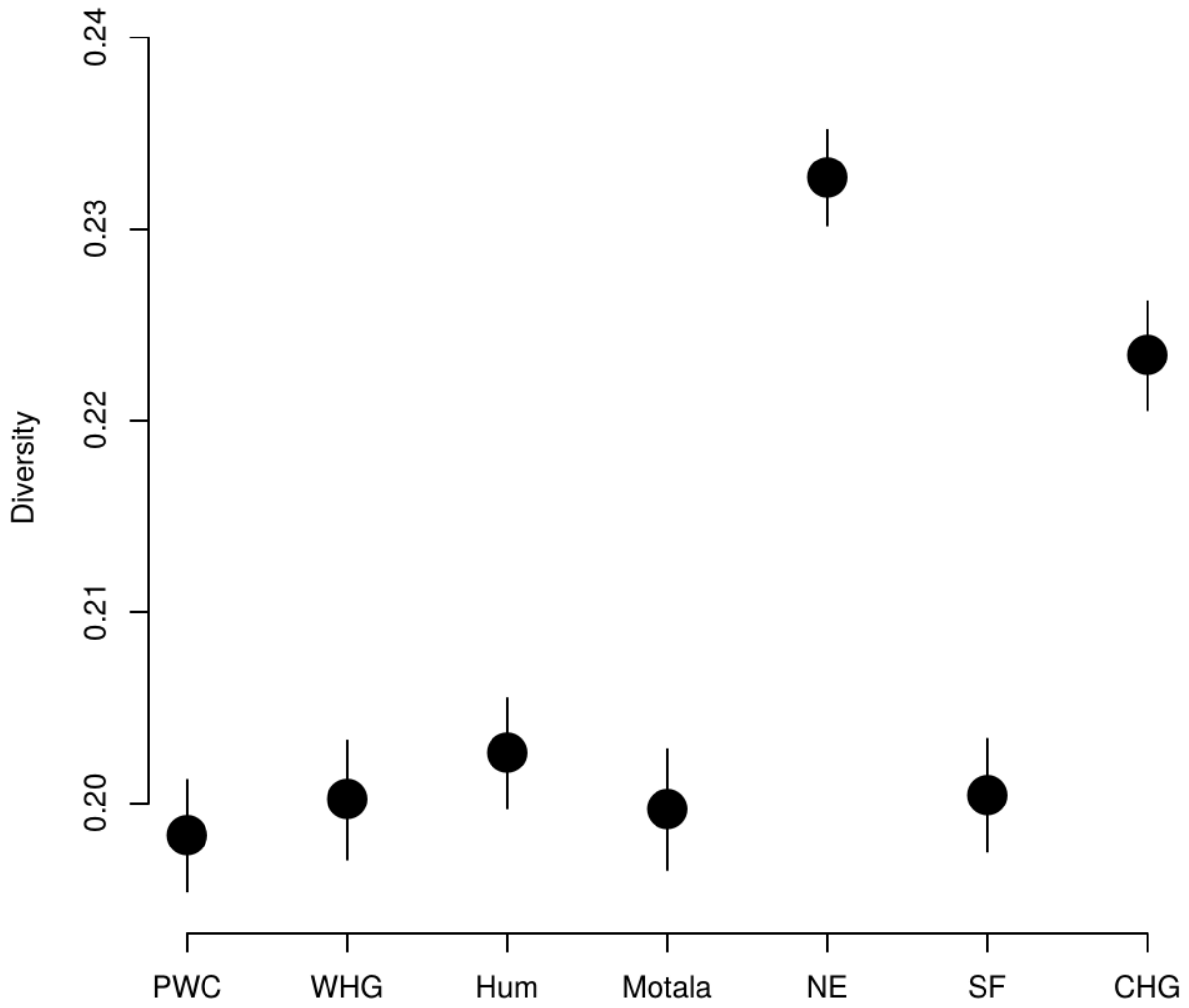

Figure S7.1 Conditional nucleotide diversity for seven prehistoric groups (using the two individuals with the highest shotgun sequencing coverage per group). Error bars show two standard errors. PWC – Pitted Ware culture, WHG – western hunter-gatherers, NE – Neolithic Hungary, CHG – Caucasus hunter-gatherers.

Furthermore, we noticed that the differences between shotgun sequenced Motala12 and Motala3 were reduced to one third when compared to other pairs of individuals from the Motala site. This is even lower than expected for first degree relatives. This reduced diversity was not observed when we used capture data for both individuals. The mitochondrial haplogroup of shotgun sequenced Motala3 (U2e1) differs from the haplogroup reported in Lazaridis et al [3] and the haplogroup of SNP capture data for the same individual (U5a1 in both cases). As the haplogroup for Motala12 is also U2e1, we suspect that the BAM file for Motala3 which was uploaded to the European Nucleotide Archive is likely from Motala12 while all SNP capture files have the correct assignments. Motala3 was excluded from all analyses based on sequence data only.

The general results of the diversity analysis is consistent with expectations [1], showing a low diversity in hunter-gatherer groups and a higher diversity in early farmers (Figure S7.1). The high diversity in CHG is notable but it might be attributable to the 3,500 years time difference between the two CHG

individuals. This analysis did not pick up substantial differences between the other hunter gatherer groups, so we continued with more fine-scale methods to measure genetic diversity (see main text).

References

1. Skoglund P, Malmstrom H, Omrak A, Raghavan M, Valdiosera C, Gunther T, et al. Genomic Diversity and Admixture Differs for Stone-Age Scandinavian Foragers and Farmers. Science. 2014;344: 747–750. doi:10.1126/science.1253448

2. Auton A, Abecasis GR, Altshuler DM, Durbin RM, Abecasis GR, Bentley DR, et al. A global reference for human genetic variation. Nature. 2015;526: 68–74. doi:10.1038/nature15393

3. Lazaridis I, Patterson N, Mittnik A, Renaud G, Mallick S, Kirsanow K, et al. Ancient human genomes suggest three ancestral populations for present-day Europeans. Nature. 2014;513: 409–413. doi:10.1038/nature13673

4. Haak W, Lazaridis I, Patterson N, Rohland N, Mallick S, Llamas B, et al. Massive migration from the steppe was a source for Indo-European languages in Europe. Nature. 2015; doi:10.1038/nature14317

5. Mathieson I, Lazaridis I, Rohland N, Mallick S, Patterson N, Roodenberg SA, et al. Genome-wide patterns of selection in 230 ancient Eurasians. Nature. 2015;528: 499–503. doi:10.1038/nature16152

6. Günther T, Valdiosera C, Malmström H, Ureña I, Rodriguez-Varela R, Sverrisdóttir ÓO, et al. Ancient genomes link early farmers from Atapuerca in Spain to modern-day Basques. Proc Natl Acad Sci USA. 2015;112: 11917–11922. doi:10.1073/pnas.1509851112

7. Cassidy LM, Martiniano R, Murphy EM, Teasdale MD, Mallory J, Hartwell B, et al. Neolithic and Bronze Age migration to Ireland and establishment of the insular Atlantic genome. Proceedings of the National Academy of Sciences. 2015; 1–6. doi:10.1073/pnas.1518445113
