# Supplementary material for: Population genomics of Mesolithic Scandinavia: Investigating early postglacial migration routes and high-latitude adaptation
Source: PLoS Biol. 2018 Jan 9;16(1):e2003703. doi: 10.1371/journal.pbio.2003703 (PMC5760011; doi:10.1371/journal.pbio.2003703)
Supplement: S8 Text — (PDF) [file pbio.2003703.s013.pdf]

S8 Functional variation in ancient samples

Methods

In order to generate a detailed portrait of the putative physical and metabolic phenotypes of Mesolithic hunter-gatherers, we screened all individuals presented in this study for variants associated with different traits of interest. We attempted to predict their physical appearance (pigmentation, scalp and facial hair phenotype), resistance to infectious diseases and other immune-related conditions, ability to digest certain animal or plant products, predisposition to genetic disorders, and other relevant conditions. The allelic states from these newly sequenced individuals were compared to those from the literature, including Motala, Western European HGs (WHG), Eastern European HGs (EHG), Caucasus hunter-gatherers (CHGs), as well as Eurasian Upper Paleolithic and Neolithic individuals to better understand the evolution and spread of these traits. Furthermore, only individuals with an average of >1x coverage for the captured SNPs were considered from [1] (ElMiron, Villabruna, GoyetQ116-1, Vestonice16 and Kostenki14). Genome sequence data and target-SNP capture data have been produced for some Motala individuals [2,3]. To jointly analyze those two types of data, we merged data per individual, after manually checking that no contradicting information was observed between the two BAM files at the investigated positions.

Both transversions and transitions were investigated for this analysis, as roughly two thirds of variants of interest are transitions. MapDamage V2.0.2-15 [4,5], was used with default parameters to rescale the base qualities of potentially deaminated sites in all BAM files. Data from individuals whose libraries had been damage-repaired (UDG treated) were not included in this base quality rescaling step. This approach may not completely remove all aDNA damages at the sequence fragment termini, but it controls for aDNA damage under a probabilistic framework by utilizing all information from the ancient genome sequence data. Samtools v1.3 [6] mpileup was used to restrict our analysis to variants with base qualities >= 30 and reads with MAPQ >= 30. The classification of published ancient samples can be found in table S8.1 and all calls at functional sites can be found in S1 Table.

Table S8.1 Samples used in the functional variants section.

Sample	Group	Group assignment for functional variant section	Country	Reference
Ajv58	NHG	Neolithic Hunter-gatherer Scandinavia	Sweden	Skoglund et al., 2014 and this study
Ajv70	NHG	Neolithic Hunter-gatherer Scandinavia	Sweden	Skoglund et al., 2014 and this study
Motala1/I0011	SHG	Mesoltihic Hunter-gatherer Scandinavia	Sweden	Haak et al 2015
Motala2/I0012	SHG	Mesoltihic Hunter-gatherer Scandinavia	Sweden	Lazaridis et al 2014, Haak et al 2015
Motala3/I0013	SHG	Mesoltihic Hunter-gatherer Scandinavia	Sweden	Lazaridis et al 2014, Haak et al 2015
Motala4/I0014	SHG	Mesoltihic Hunter-gatherer Scandinavia	Sweden	Lazaridis et al 2014, Haak et al 2015
Motala6/I0015	SHG	Mesoltihic Hunter-gatherer Scandinavia	Sweden	Lazaridis et al 2014, Haak et al 2015
Motala12/I0017	SHG	Mesoltihic Hunter-gatherer Scandinavia	Sweden	Lazaridis et al 2014, Haak et al 2015
Steigen	SHG	Mesoltihic Hunter-gatherer Scandinavia	Norway	This study
SF9	SHG	Mesolithic Hunter-gatherer Scandinavia	Sweden	This study
SF11	SHG	Mesoltihic Hunter-gatherer Scandinavia	Sweden	This study and Skoglund et al 2014
SBj	SHG	Mesoltihic Hunter-gatherer Scandinavia	Sweden	This study
Hum2	SHG	Mesoltihic Hunter-gatherer Scandinavia	Norway	This study
Hum1	SHG	Mesoltihic Hunter-gatherer Scandinavia	Norway	This study
SF12	SHG	Mesoltihic Hunter-gatherer Scandinavia	Sweden	This study
LaBrana1	WHG	Mesoltihic Hunter-gatherer West Europe	Spain	Olalde et al 2014
Loschbour	WHG	Mesoltihic Hunter-gatherer West Europe	Luxembourg	Lazaridis et al 2014
Bichon	WHG	Mesoltihic Hunter-gatherer West Europe	Switzerland	Jones et al. 2015
KO1	WHG	Mesoltihic Hunter-gatherer West Europe	Hungary	Gambaet al. 2014
UzOO74/I0061	EHG	Mesoltihic Hunter-gatherer East Europe	Russia	Mathieson et al. 2015
SVP44/I0124	EHG	Mesoltihic Hunter-gatherer East Europe	Russia	Mathieson et al. 2015
Kotias	CHG	Mesoltihic Hunter-gatherer Caucasus	Georgia	Jones et al. 2015
Satsurblia	CHG	Mesoltihic Hunter-gatherer Caucasus	Georgia	Jones et al. 2015
ElMiron	PEHG	Paleolithic Hunter-gatherer Europe	Spain	Fu et al. 2016
Villabruna	PEHG	Paleolithic Hunter-gatherer Europe	Italy	Fu et al. 2016
GoyetQ116-1	PEHG	Paleolithic Hunter-gatherer Europe	Belgium	Fu et al. 2016
Vestonice16	PEHG	Paleolithic Hunter-gatherer Europe	Czech Republic	Fu et al. 2016
Kostenki14	PEHG	Paleolithic Hunter-gatherer Europe	Russia	Fu et al. 2016
Klei10	EEF	Early Neolithic Balkans	Greece	Hofmanova et al. 2016
Rev5	EEF	Early Neolithic Balkans	Greece	Hofmanova et al. 2016
Pal7	EEF	Early Neolithic Balkans	Greece	Hofmanova et al. 2016
Bar8	EEF	Early Neolithic Anatolian	Turkey	Hofmanova et al. 2016
Bar31	EEF	Early Neolithic Anatolian	Turkey	Hofmanova et al. 2016
Tep002	EEF	Early Neolithic Anatolian	Turkey	Kılınç et al. 2016
Tep003	EEF	Early Neolithic Anatolian	Turkey	Kılınç et al. 2016
Tep004	EEF	Early Neolithic Anatolian	Turkey	Kılınç et al. 2016
Tep006	EEF	Early Neolithic Anatolian	Turkey	Kılınç et al. 2016
Bon001	EEF	Early Neolithic Anatolian	Turkey	Kılınç et al. 2016
Bon002	EEF	Early Neolithic Anatolian	Turkey	Kılınç et al. 2016
Bon004	EEF	Early Neolithic Anatolian	Turkey	Kılınç et al. 2016
BAR2/I0707	EEF	Early Neolithic Anatolian	Turkey	Mathieson et al. 2015
BAR6/I0708	EEF	Early Neolithic Anatolian	Turkey	Mathieson et al. 2015
BAR20/I0709	EEF	Early Neolithic Anatolian	Turkey	Mathieson et al. 2015
M11-363/I0745	EEF	Early Neolithic Anatolian	Turkey	Mathieson et al. 2015
L11-322/I0746	EEF	Early Neolithic Anatolian	Turkey	Mathieson et al. 2015
L14-200/I1583	EEF	Early Neolithic Anatolian	Turkey	Mathieson et al. 2015
Stuttgart	EEF	Early Neolithic Central European	Germany	Lazaridis et al. 2014
HAL2/I0659	EEF	Early Neolithic Central European	Germany	Haak et al. 2015, Mathieson et al. 2015
HAL24/I0821	EEF	Early Neolithic Central European	Germany	Haak et al. 2015, Mathieson et al. 2015
HAL25/I0048	EEF	Early Neolithic Central European	Germany	Haak et al. 2015, Mathieson et

				al. 2015
HAL34/I0057	EEF	Early Neolithic Central European	Germany	Haak et al. 2015, Mathieson et al. 2015
HAL4/I0100	EEF	Early Neolithic Central European	Germany	Haak et al. 2015, Mathieson et al. 2015
HAL5/I0046	EEF	Early Neolithic Central European	Germany	Haak et al. 2015, Mathieson et al. 2015
KAR16A/I0797	EEF	Early Neolithic Central European	Germany	Haak et al. 2015, Mathieson et al. 2015
KAR6	EEF	Early Neolithic Central European	Germany	Haak et al. 2015, Mathieson et al. 2015
LBK1976/I0022	EEF	Early Neolithic Central European	Germany	Haak et al. 2015, Mathieson et al. 2015
LBK1992/I0025	EEF	Early Neolithic Central European	Germany	Haak et al. 2015, Mathieson et al. 2015
LBK2155/I0026	EEF	Early Neolithic Central European	Germany	Haak et al. 2015, Mathieson et al. 2015
SZEH4/I0176	EEF	Early Neolithic Central European	Hungary	Haak et al. 2015
Starcevo	EEF	Early Neolithic Central European	Hungary	Haak et al. 2015
Troc1/I0409	EEF	Early Neolithic Iberian	Spain	Haak et al. 2015
Troc3/I0410	EEF	Early Neolithic Iberian	Spain	Haak et al. 2015
Troc5/I0412	EEF	Early Neolithic Iberian	Spain	Haak et al. 2015
Troc7/I0413	EEF	Early Neolithic Iberian	Spain	Haak et al. 2015

Finally, it is noteworthy that the variants used in our subsequent phenotypic predictions have been validated in present-day human populations, thus it is possible that undetected functional variation within the analyzed genes (or even other regions) contributed to phenotypic variation in ancient human populations. Such variation would not be captured by the following analyses [2].

S8.1 Pigmentation

Predicting human eye, hair, and skin pigmentation scales and color has recently been reported by analyzing genetic variation at several genes and SNPs [7–12]. Here we reconstructed the phenotypic appearance of the SHGs, and screened these functional variants in other hunter-gatherer individuals as well as Early Neolithic farmers across Europe, and thus improved the understanding of the history and evolution of these variants.

Eye, hair, and skin color/pigmentation was assessed by screening two sets of pigmentation-associated markers recently validated in present-day human populations [10,12–14]. The probability for certain eye and hair color for each ancient individual was computed using the enhanced version 1.0 Hirisplex Microsoft Excel macro [14], a SNP system originally designed for forensic purposes. We attempted to predict skin pigmentation using the 8plex system described by Hart et al [12], but as previously reported [2,15], the results were inconclusive for ancient samples using this panel. Similar to previous investigations of ancient humans [2,15–17], we provide skin-color predictions based on the genetic variants at the rs16891982 (in the *SLC45A2* gene) and rs1426654 (*SLC24A5*) SNPs. The derived allele at rs1426654 (*SLC24A5*) has the greatest effect on skin pigmentation among Europeans [18–21]. Moreover, this variant is part of a light skin haplotype termed "*C11*", which is defined by a set of 16 SNPs across 78kb within the *SLC24A5* gene. In addition to rs1426654, we investigated the non-synonymous SNP rs16891982 in the *SLC45A2* gene, which is a second major-effect pigmentation locus in Europeans [22]. The derived alleles at both rs1426654 and rs16891982 positions are found at extremely high frequencies in present-day European populations (where the former allele is virtually fixed and the latter is ~90%), an observation that has been interpreted as the signature of recent positive selection in Europeans [23].

In studies of eye pigmentation, the derived allele at SNP rs12913832 (within the *HERC2* gene) has been associated with iris depigmentation. Individuals carrying the homozygous derived variant exhibit a blue eye-color, whereas carriers of the heterozygote genotype or the homozygote ancestral allele display an intermediate or a brown eye-color phenotype [24]. Across present-day Europeans populations from the 1000 genomes project, the blue eye color associated variant is found at an average frequency of 60%, and it reaches its highest frequency in the Finnish population (90%) and the British population (81%) [25]. The SNP rs12913832 is part of the *h-1* haplotype, which is carried by 97% of blue-eyed individuals in a present-day study population from Turkey, Jordan, and Denmark [26]. The presence of *h-1* can be assessed through screening the allelic states at a set of 13 SNPs in the *OCA2/HERC* region.

Allelic states of the investigated SNPs as well as the *C11* and *h-1* haplotypes for each ancient group is shown in S1 Table. A brief summary of the pigmentation phenotypic reconstruction can be found below for each group and individual, as well as interpretations of the history of these variants.

Western hunter-gatherers (WHGs)

The Mesolithic La Braña, Loschbour WHGs, the Upper Palaeolithic Villabruna individual, and the Neolithic KO1 individual, which has a genetic makeup of a WHGs [27], had dark skin and blue eyes based on their allelic states at the rs1426654, rs16891982 and rs12913832 SNPs [1,2,16,27]; an observation supported by their high probabilities of blue eye color inferred by Hirisplex in this study. The Upper Paleolithic Bichon from central Europe [15] had likely dark-skin and brown eyes (S1 Table). The dark skin and blue eyes phenotype combination was likely very common among WHGs and it has been inferred for four out of five Mesolithic and Upper Paleolithic individuals in western and central Europe. Moreover, the allelic states at the *h-1* defining positions suggest that La Braña, Loschbour and Villabruna carried this haplotype. The haplotype determination was inconclusive for the remaining individuals due to alternative alleles and missing data at informative positions. All individuals from this group were predicted to have a dark hair color, where Loschbour, Villabruna and Bichon probably had black hair, whereas La Braña and KO1 present similar probabilities of having brown or black hair (S1 Table).

Eastern hunter-gatherers (EHGs)

The Karelian and Samaran Russian Mesolithic hunter gatherers [3] are currently the best representatives of a group of EHGs that migrated and admixed with WHGs to form SHGs. Mathieson et al [28] recently reported data from another Karelian Mesolithic hunter gatherer (sample I0211), with low coverage (0.136X across all captured SNPs), preventing pigmentation characterization. Hirisplex skin and hair color predictions suggest some opposing pigmentation patterns for the EHG individuals. The Karelian individual presents high probabilities of being brown-eyed (0.99), and having a dark hair (0.96). Without speculating about the genetic architecture of skin pigmentation, we suggest an intermediate skin-pigmentation phenotype for the Karelia individual, as it carried the ancestral allele at rs16891982 and the derived allele at rs1426654 (S1 Table). The presence of the rs1426654 light-skin allele, in addition to five additional *C11*-associated alleles at haplotype defining SNPs (S1 Table) suggests that the Karelian individual carried the *C11* light-skin haplotype. The Samaran individual exhibits high probabilities of being blue-eyed (0.88), light hair shade (0.99); most likely being blond (0.75). The two skin pigmentation SNPs suggest that the Samaran individual was light-skinned. In summary, the EHGs had high frequencies of the light-skin variants and intermediate frequencies of the blue-eye variants.

Scandinavian hunter-gatherers (SHGs)

The SHG group includes the seven individuals from this study, the six Motala hunter-gatherers [2], and the Neolithic Ajvide 58 hunter-gatherer [29]. From SHGs sequenced in this study, we obtained an eye and hair pigmentation portrait for SF9, Hum2, SBj and SF12. No pigmentation predictions were generated for the SF11, Hum1 and Steigen samples, due to missing data at the relevant positions. Out of the four individuals with a Hirisplex prediction, both light and dark pigmentation phenotypes were observed. SBj and SF12 exhibited high probabilities of being blue-eyed (0.91 and 0.88, respectively), while SF9 and Hum2 were predicted to have been brown eyed. The high-coverage and high quality genome of SF12 carried two copies of the *h-1* haplotype (defined by the homozygous state of all *h-1* haplotype SNPs, S1 Table). SF12 and Hum2 likely had dark hair (p=0.75 and p=0.99), while SF9 and SBj had light hair (p=0.58 and p=0.90). SBj presented slightly higher probabilities of being blond (0.52) than having black, brown or red hair.

The common feature of the skin-pigmentation SNPs among these Scandinavian hunter-gatherers was a mix of light and dark skin alleles at either rs16891982 or rs1426654 (S1 Table). The Hum2 individual exhibited only derived haplotype-associated alleles at the 16 *C11*-defining positions, suggesting that Hum2 had at least one copy of this haplotype. SF9 and Steigen present derived variants at 8 and 13 out of the 16 screened positions, respectively. Even though both Steigen and SF9 lacked information for the core SNP rs1426654, Steigen only carried C11-associated alleles at rs1834640, rs2675345, rs938505 positions, and SF9 at rs1834640 and rs938505, which distinguish C11 from other *SLC24A5* haplotypes. SF12 exhibits a different haplotype, C9, compared to the other SHGs, and carries the ancestral allele at the core SNP rs1426654, indicating dark skin pigmentation.

A Hirisplex eye and hair color prediction was obtained for the six hunter-gatherers from Motala [2,3]. Interestingly, all individuals exhibited high probabilities of being blue-eyed (0.71-0.92). The Motala2, Motala3, Motala4 and Motala12 individuals most likely had a dark hair color (0.70-0.99), while Motala1 and Motala6 had a light shaded hair (~0.91); they may have been blond (~0.60). Similar to SF9, SF11, SF12, SBj, Hum1, Hum2 and Steigen, the Motala hunter-gatherers presented a combination of light and dark skin pigmentation alleles. Only Motala2 presented exclusively light-skin variants at both rs16891982 and rs1426654. Motala12 exhibited only haplotype-defining derived alleles at both the *h-1* and the *C11* associated positions. The combination of C11-associated alleles at the rs1834640 and rs2675345 SNPs with a light-pigmentation allele at the core SNP rs1426654, suggest that Motala2 and Motala4 individuals carried the *C11* haplotype as well.

The Neolithic Ajvide 58 and 70 individuals from a Pitted Ware Culture context shares part of its phenotypic variation with SHGs. While they presented high probabilities of being blue-eyed (0.79 and 0.86) and having a dark hair color (0.94 and 0.97), Ajv58 exhibits the dark skin pigmentation alleles at rs16891982 and rs1426654 positions, while Ajv70 is heterozygous at those positions..

Interestingly, the eye and light skin pigmentation phenotypes observed in all SHGs could potentially be explained by admixture between WHG and EHG groups. The high relative-frequency of the blue-eye color allele in SHGs, resembles WHG, while the intermediate frequencies of the skin color determining SNPs in SHGs seem more likely to have come from EHG, since both light-pigmented alleles are virtually absent from WHG. However, for all three well-characterized skin and eye-color associated SNPs, the SHGs display a frequency that is greater for the light-skin variants and the blue-eye variant than can be expected from a mixture of WHGs and EHGs. This observation indicates that the frequencies may have increased due to continued adaptation to a low light conditions.

Caucasus hunter-gatherers (CHGs)

Both Kotias and Satsurblia CHGs [15] were predicted by Hirisplex to have brown eyes (>0.96) and a dark hair shade (>0.92). Looking at skin-pigmentation sites, both individuals carried the dark-skin allele at rs16891982 and the light-pigmentation allele at rs1426654. Similar to the Hum2 and Motala12 individuals, Kotias showed exclusively haplotype-associated alleles at the *C11*-defining positions (with at least 5 reads of such alleles per site), suggesting it carried the *C11* haplotype.

Palaeolithic European hunter-gatherers (PEHG)

The Hirisplex eye and hair color prediction for the Paleoltihic European hunter gatherers ElMiron, GoyetQ116-1, Vestonice16, Kostenki14 (dated from 18,000 to 36,000 ya), displayed high probabilities of being brown-eye color (> 0.99), and high probabilities of exhibiting a dark hair pigmentation (0.60-0.99). All four individuals presented only dark-skin alleles at rs16891982. The GoyetQ116-1 and Vestonice16 individuals missed information at the rs1426654, while El Miron carried only dark-skin alleles at that position, and Kostenki14 had four dark-skin alleles and one light-pigmentation allele.

Early European farmers (EEF)

The Hirisplex eye and hair color prediction of 37 Early Neolithic farmers sequenced across Europe and Anatolia [2,3,17,30] revealed that four individuals presented high probabilities of being blue-eyed (p=0.55-0.91), 23 were predicted to be brown-eyed (p=0.76-0.99), and 10 individuals did not have enough data to make a prediction. In total 23 individuals exhibit a high probability for dark hair pigmentation (p=0.57-0.99), five had most likely a light hair shade; the remaining nine farmers lacked data for meaningful hair pigmentation prediction (S1 Table). The Anatolian Barcin I1583 individual [28] exhibited blue-eye color variants at the core *h-1* SNP rs12913832 and its linked rs1129038 variant. Interestingly, it also presented *h-1*-haplotype-defining alleles at rs7170852, rs2240203, rs916977, suggesting that this individual might have harbored the blue-eye color founder haplotype as well. No other early farmer presented direct evidence for the *h-1* haplotype's presence.

While both pigmentation alleles were observed at rs16891982 (although the derived allele in much higher proportion, see below), virtually only the light-skin allele was observed at rs1426654. Unequivocal evidence for the presence of the *C11*-haplotype was observed for the central European Stuttgart sample, while (although at low coverage) only haplotype-associated alleles were observed in the Greek Klei10 early farmer individual.

Summary

After investigating the presence of light-pigmentation alleles and haplotypes in different hunter-gatherers and early farmers across Europe and Anatolia, it seems as if eye and light skin pigmentation alleles entered Europe several times during different migration events. In particular, the light skin pigmentation variant arrived to Scandinavia already in the Mesolithic with migrants from the northeast, whereas the blue-eye variants probably arrived in Scandinavia with migrants from the south.

Light eye pigmentation variants were present at high frequencies in WHG, SHG, EHG and EEF (not present in PEHG), while the blue-eye color founder haplotype *h-1* was found in the La Brana, Loschbour, Villabruna WHGs, SF12, Motala1 and Motala12 SHGs and at least one early farmer. Such results suggest that the blue eye-color allele is rather old. Using an ABC modeling approach Nakagome et al. [31], predicted that the light-pigmentation allele at rs12913832 emerged around 42,000 years ago or earlier; a date close in time to the initial peopling of Europe. A plausible scenario of the origin of the blue-eye mutation that reconciles our results with findings from other studies is one where this variant appeared in an ancestral population before the ancestors of the WHG migrated from Near East into West and Central Europe [1].

The large effect light-skin alleles at rs16891982 and rs1426654 were present in SHG, EHG, CHG and EEF but absent in WHG and PEHG. Similarly, the *C11* haplotype is present in hunter-gatherers (SHG, EHG and CHG but not WHG and PEHG) throughout Europe, as well as in at least two early farmers. This pattern is consistent with reports that the rs1426654 derived allele arose ~22,000-28,000 years ago [31,32], and that the light-pigmentation allele at rs16891982 arose only once in Eurasians [31,33]. A possible geographical origin for these two major light-skin alleles is West Asia or the Near East [34]. Later migrations across the Caucasus (CHG) and Eastern Europe would have brought it to Scandinavia, while EEF migrations introduced both alleles into central Europe.

Moreover, [21] suggested that selective sweeps on rs1426654 and rs16891982 (light-pigmentation alleles), started at between ~15-19 kya (under a dominant model) and ~11-13 kya (under an additive model), respectively. In a comparison of SNP capture data of hunter-gatherers of mixed geographic origin, early farmers and modern-day Europeans, Mathieson et al [28] suggested that while light-pigmented alleles frequencies in extant Europeans at rs1426654 could be explained by demography, variation at the rs16891982 locus produced the second highest genome-wide selection signal observed in their study. These results, of a high frequency of rs1426654 light-skin allele in Mesolithic Scandinavia and Eastern Europe, at a time when it is not seen in central Europe, supports a scenario of environmental adaptation to northern latitudes.

To summarize the results of the skin and eye color section, we display the allele frequencies of light-pigmentation alleles of the three SNPs rs12913832, rs16891982 and rs1426654 for each group in Figure 3. Allele frequencies are estimated as the proportion of chromosomes carrying the light pigmentation allele. A distinction was made between low coverage (haploid calls) sites (SNPs covered by less than five reads in a particular individual), versus higher coverage (diploid calls) sites (SNPs covered by five or more reads in a particular individual). The maximum count of light-pigmentation alleles at haploid sites was 1, and 2 at diploid sites. Positions where both alleles were observed contributed either 0.5 (haploid call) or 1 (diploid call) to light-color allele frequencies in order to be consistent with only calling 2 alleles if we have 5 or more reads covering a site. Data from all individuals was used to calculate standard errors for each estimate.

S8.2 Scalp and facial hair associated SNPs

Genetic studies in modern-day humans have found certain SNPs to be associated with scalp hair (shape, greying, balding) and facial hair (beard thickness, monobrow, eyebrow thickness) [35]. Here, we investigate these SNPs in ancient Scandinavian individuals. Table S8.2 summarizes the results.

Regarding hair shape, we screened SNPs rs3827760 and rs11803731, where variants have been associated with straight hair in Europeans and Asians. Furthermore, it has been suggested that the associated variants have been under recent selection in both populations [36,37]. At rs11803731, we find both the associated and non-associated variant among SHGs (SF12 is homozygote for the straight-hair associated variant), which is similar to other hunter-gatherers throughout Europe. For rs3827760, within the EDAR gene, the derived G allele is associated with shovel-shaped teeth and hair thickness phenotype in East Asians. In the novel SHGs in this study, only the ancestral A allele is present (SF12 is homozygote AA). The derived variant was reported in three of the six Motala SHGs which are younger than most other SHGs in this study [28]. It is clear that the variant was present among SHGs [28], and it is possible that it has a continuous (but varying) distribution from Scandinavia to East Asia during the Mesolithic, and that the very low sample size of EHGs has failed to pick up the variant. It is

also possible that the derived rs3827760 variant was brought to Scandinavia by migration in the Late Mesolithic, perhaps related to the specific Motala group.

In total 13 SNPs have been associated to facial hair presence and shape [35]. Among those positions, rs365060, rs4864809, rs6901317 and rs117717824 have been linked to beard thickness, rs112458845 to eyebrow thickness and rs2218065 to monobrow. These SNPs were ascertained in admixed Latin American populations and it is still unclear how well these variants influence facial hair presence and shape in non-Latin Americans. These are also highly polygenic traits, so the markers investigated here do not allow for reliable conclusions regarding the ancient individuals. Nonetheless, in order to get an indication for these traits among the ancient individuals, we investigated these SNPs.

For beard thickness, 2 SNPs (rs365060 and rs117717824) showed opposing patterns of only the thick beard variant for the former SNP, and only the thin beard variant for the latter SNP. For the 2 remaining beard thickness SNPs, we observe both variants (2 of 5 vs 3 of 5 for rs4864809) and 5.5 of 8 vs 2.5 of 8 in rs6901317). Hence, the SHGs would, on average, have intermediate and varied beard thickness. While SF12 is a female (she is heterozygote for 2 of the SNPs, and homozygote thick beard for one site and homozygote thin beard for one site), Steigen is a male and has the thick beard variant at 2 sites, possibly heterozygote at one site and carries one thin beard variant at the 4th site. Interestingly, the thick beard variant at rs365060 has been shown to be under selection in modern-day western Europeans [35]. A comparison of the allelic state of the individuals from this study to other HGs, showed that the thick beard variant at rs365060 was widely spread within WHGs, EHGs and CHGs. Thus, a selective event probably predated the split of hunter-gatherers in Europe.

The eyebrow thickness associated SNP rs112458845 showed variation among SHGs (3As and 2Gs called), whereas the monobrow variant G (rs2218065) appears to be in high frequency (6 of 6) among SHGs. SF12 was homozygote for both the thick eyebrow and monobrow variants. The gray hair associated variant was also quite common among SHGs (4 of 5).The SNPs rs2814331 and rs4258142 have been associated to balding [35,38]. Among all 7 individuals, we only find balding associated variants at these two SNPs (3 individuals had no data on these SNPs).

Table S8.2 Scalp and facial associated SNPs

	**SNPID**	**gene**	**mutation**	**strand**	**Steigen**	**SF9**	**SF11**	**SBj**	**Hum2**	**Hum1**	**SF12**
Hair shape	rs11803731	*TCHH*	A > T	+	NA	1A	NA	NA	2T	NA	77T
Hair shape	rs3827760	*EDAR*	A > G	-	1A	1A	NA	NA	8A	NA	88A
Beard thickness	rs365060	*EDAR*	G > C	+	2C	NA	NA	NA	4C	NA	85C
Beard thickness	rs4864809	*LNX1*	A > G	+	2G	1G	NA	NA	3A	NA	29G,23A
Beard thickness	rs6901317	*PREP*	G > T	+	1T,1G	1G	1G	NA	2G	1G	35T,35G
Beard thickness	rs117717824	*FOXP2*	G > T	+	2G	2G	NA	NA	1G	1G	54G
Eyebrow thickness	rs112458845	*FOXL2*	G > A	+	NA	NA	NA	1G	2G	NA	31A
Monobrow	rs2218065	*PAX3*	A > G	+	2G	1G	NA	NA	1G	NA	41G
Hair greying	rs12203592	*IRF4*	C > T	+	NA	NA	NA	NA	4T	2T	40C,34T
Balding	rs2814331	*GRID1*	C > T	+	1T	1T	NA	NA	NA	NA	45T
Balding	rs4258142	*AR/EDA2R*	C > T	+	3T	2T	NA	NA	2T	NA	24T

S8.3 Acetylator phenotype

The genetic basis of drug response is a major focus of pharmacogenetics. Drugs are metabolized using metabolic pathways, which evolved to degrade harmful metabolic by-products, as well as dietary and environmental toxins. Therefore, individual drug response constitutes a complex genetic trait shaped by both genetic and environmental pressures. N-acetylotransferase 2 gene (*NAT2*) is involved in the metabolism of a wide variety of foreign chemical substances (xeniobiotics) [39]. The metabolism can

be either fast or slow depending on the polymorphisms within NAT2 gene. This differential drug response has also been linked to response to a number of carcinogens, especially those causing bladder and colon cancers [40]. The global distribution of the two phenotypes suggests the incidence of the fast-acetylator phenotype is higher in modern hunter-gatherers, Native Americans and East Asians than in Africans and West Eurasians. The prevalence of each of the phenotypes has been reported to correlate with the type of subsistence practiced by various populations, ranging from high frequency of fast phenotype among hunter-gatherers (>77%) to medium levels among pastoralists and agriculturalists (51-54.9%) [41]. There is evidence from present-day populations for selection favoring the slow-acetylator phenotype, which was hypothesised to be related to dietary changes accompanying the transition to agriculture [42,43]. Traditionally, *NAT2* phenotype is predicted based on phased *NAT2* haplotypes. However, in the absence of phased data, genome-wide shotgun sequence data from 7 informative SNPs (rs1801279, rs1801280, rs1799930, rs1799931, rs1041983, rs1799929, rs1208) can be used in phenotype prediction. The methodology was adopted in an online *NAT2* prediction tool - NAT2PRED (http://nat2pred.rit.albany.edu/) [44]. Alternatively to the 7-SNP phenotype prediction panel it is possible to use a single tag SNP (rs1495741) and/or a 2-SNP panel (rs1041983 and rs1801280) [45]. In general, presence of ancestral alleles is associated with the presence of rapid metabolizer phenotypes. It has however been suggested that the 2-SNP panel outperforms the prediction power of the single tag SNP, which may be Caucasian specific [46]. We have used the combination of all three methods to predict the acetylation state in 6 out of 7 investigated individuals. The 7-SNP panel was only useful for two individuals: SF12 and Hum2. While SF12 was estimated to be a slow acetylator, this type was previously described in the Stuttgart farmer [2], Hum2 was probably an intermediate or rapid acetylator (similarly to Motala12 and Loschbour foragers). Remaining individuals did not have enough genome coverage for reliable prediction based on the 7-SNP system. However, using the tag single- and two-SNP panels, it was possible to predict the following phenotypes: Steigen (slow), SF9 (slow), SF11 (intermediate). Thus, the Scandinavian hunter-gatherers from this study exhibit the whole range of acetylator phenotypic variation with excess of slow acetylator phenotype (N=4) over the rapid and intermediate phenotypes (N=3). While those phenotype frequencies are similar to those observed among present day Europeans, they deviate from previous observations of modern-day and two ancient hunter-gatherers [2,41]. In order to further investigate this scenario, we screened the aforementioned markers in 17 other European hunter-gatherers. We were able to assign the 7-SNP acetylator phenotypes in five of the tested individuals. Two of the individuals were slow (Bichon, Kotias), two were intermediate (LaBrana, Loshbour) and the Motala12 individual was a rapid metabolizer (based on NATPRED and 2-SNP prediction). We then applied the tag-SNP (rs1495741) in order to increase the number of individuals and managed to assign the metabolizer state in 17 hunter-gatherers (S1 Table). According to tag-SNP analyses only one individual was a rapid metabolizer (KO1), five were intermediate metabolizers (Motala4, Motala12, Loshbour, Vestonice16 and Kostenki14), while 9 foragers were slow metabolizers (Ajvide58, Motala6, LaBrana, Bichon, Kotias, Karelia, Samara, ElMiron and Villabruna). In Motala12 we see an inconsistency between the scoring systems, where both 7-SNP and the 2-SNP give the same type, while the phenotype based on the 1 tag SNP system differs.

Identified mixed phenotypes with a slight excess of slow metabolizers in populations pre-dating introduction of farming, contradicts the widely held view that the slow-acetylator phenotype arose as a result of dietary changes accompanying the transition to agriculture [42,43], however, it might have increased its frequency as a consequence of adaptation to new dietary habits.

Table S8.3 Allelic state of *NAT2* associated SNPs in all seven new SHGs.

SNPID	gene	mutation	strand	Steigen	SF9	SF11	SBj	Hum2	Hum1	SF12
rs1801279	*NAT2*	G > A	+	3G	2G	NA	NA	1G	NA	79G
rs1801280	*NAT2*	T > C	+	NA	1T	NA	NA	1T,1C	NA	57T
rs1799930	*NAT2*	G > A	+	NA	1G	1A*	NA	4G	NA	46A
rs1799931	*NAT2*	G > A	+	2G,1A	NA	NA	1G	8G	1A	68G
rs1495741#	*NAT2*	G > A	+	1A	2A	1G,1A	NA	1G	NA	62A
rs1041983	*NAT2*	C > T	+	NA	1C	1C	NA	5C	NA	49T
rs1799929	*NAT2*	C -> T	+	NA	1C	NA	NA	NA	NA	55C
rs1208	*NAT2*	A > G	+	1G	NA	NA	1A	5G,5A*	1A	41A

Table S8.4 Predicted acetylation status in seven new SHGs using the 7-SNP panel (NAT2PRED: http://nat2pred.rit.albany.edu/) (Kuznetsov *et al.* 2009), 2-SNP panel and the rs1495741 tag SNP.

SNPID	NAT2 allele	Steigen	SF9	SF11	SBj	Hum2	Hum1	SF12
rs1801279	G191A	(+)	(+)	nd	nd	(+)	nd	(+)
rs1801280	T341C	nd	(+)	nd	nd	(+/-)	nd	(+)
rs1799930	G590A	nd	(+)	(-)	nd	(+)	nd	(-)
rs1799931	G857A	(+/-)	nd	nd	(+)	(+)	(-)	(+)
rs1041983	C282T	nd	(+)	(+/-)	nd	(+)	nd	(-)
rs1799929	C481T	nd	(+)	nd	nd	(-)	nd	(+)
rs1208	A803G	(-)	nd	nd	(+)	(+/-)	(+)	(+)
NAT2PRD prediction		***nd***	***nd***	***nd***	***nd***	***interm.***	***nd***	***slow***
rs1495741	# tag SNP	(-)	(-)	(+/-)	nd	(+)	nd	(-)
rs1495741 prediction		***slow***	***slow***	***interm.***	***nd***	***rapid***	***nd***	***slow***
rs1801280	T341C	nd	(+)	nd	nd	(+/-)	nd	(+)
rs1041983	C282T	nd	(+)	(+/-)	nd	(+)	nd	(-)
2-SNP prediction		***nd***	***nd***	***nd***	***nd***	***rapid***	***nd***	***interm.***

(+) indicative of presence of a fast metabolizer *NAT2*4* allele
(-) indicative of presence of a slow metabolizer *NAT2*4* allele
(+/-) intermediate allele
rs1495741 is a prediction tag where genotypes are interpreted as metabolizers: (A;A) slow, (A;G) intermediate, (G;G) rapid

S8.4 Diet and taste related SNPs

We have typed all 7 SHG individuals from this study at a number of SNPs of known dietary function, closely linked to Vitamin D levels and rickets in modern populations. All those sites have been suggested to have undergone recent positive selection [28,47], and in the case of the SHGs of this study they may have conferred local adaption to high latitude climates.

Blood levels of circulating vitamin D are the result of diet, exposure to sunlight and genetic predisposition [48]. Some of those genetic components include the genes *DHCR7* and *NADSYN1* which are associated with circulating vitamin D levels. All sites associated with circulating vitamin D levels show high levels of heterozygosity among SHGs, clearly observed in SF12 and Hum2. Furthermore, high diversity was found among other tested ancient hunter-gatherer groups including WHGs, EHGs and CHGs (see S1 Table).

FADS1 and *FADS2* are involved in fatty acid metabolism and the derived rs174546 SNP has been linked to lower triglyceride levels in Europeans [49]. The high coverage individual (SF12) was a heterozygote at the site, while two other individuals were carriers of a single C allele associated with increased LC-PUFAS levels and one non-associated allele T respectively (SBj and SF9) [50]. Fumagalli et al [51] found selection on the *FADS* region in Greenlandic Inuit (GI) populations, which is thought to be an environmental adaptation. The presence of the derived T allele at position rs7115739 has been associated with reduced height and Body-Mass-Index (BMI) in GI. The authors also found similar albeit weaker associations in Europeans between the derived alleles (T) on rs7115739 and rs174570 and reduced height [51]. Neither Hum2 nor SF12 were carriers of derived allele at rs7115739 (T), however, almost all ancient individuals were homozygous for the associated alleles at rs174602 and rs174570 (*FADS1-3*) with only SF12 being heterozygous at rs174570. The latter is associated with low fasting serum insulin, LDL and cholesterol levels in Europeans [51]. Variation in *FADS1-3* probably reflects interactions between *FADS1* and the dietary intake of omega-3 and omega-6 fatty acids [52].

The ability to digest sugars found in milk during adulthood is widely perceived as an important cultural adaptation in humans. Initially thought to have been spread with early farmers, the ability to digest milk has now been shown to be uncommon among early farmers and likely only increased in frequency in Europe during the Iron Age [53]. We tested all our ancient individuals at both *LCTa* (rs4988235) and *LCTb* (rs182549) loci and found that the three individuals for whom there was overlapping data, were all carriers of non-lactase persistence alleles, indicating inability to digest milk in adulthood, thus in line with previous observations.

Additionally, we screened polymorphisms at two SNPs loci linked to celiac disease which have been identified as having undergone recent selection within the *SLC22A4* and *ATXN2* genes [28]. The two SNPs in *SLC22A4* show a consistent pattern in which rs1050152 is a C variant not associated with increased risk of Crohn's disease (also in 12 other HGs) while rs272872 is heterogeneous among all investigated HGs. Furthermore, *ATXN2* associated with celiac disease displays a derived non associated T in four SHG individuals and 16 other hunter gatherers with available data at this position. The identification of only non-associated alleles at position rs1050152 in our dataset as well as in 12 other ancient hunter gatherers is concordant with Mathieson et al. [28] who suggested that the disease causing variant (L503F) did not reach significant frequency until recent times.

Finally, according to Fumagalli et al. [51] *WARS2/TBX15* might convey adaptation to cold in Greenlandic Inuits by decreasing the waist/hip ratio and possibly influencing the regulation of brown

and beige adipose tissue. Subsequently, the type of tissue can influence lipid oxidation and increase of heat generation in response to cold. We found ancestral rs2298080 alleles in Steigen and Hum1, while both Hum2 and SF12 were heterozygotes.

Table S8.5 Diet related SNPs.

SNPID	gene	mutation	strand	Steigen	SF9	SF11	SBj	Hum2	Hum1	SF12
rs182549	*LCTb*	C > T	+	NA	NA	NA	NA	7C	NA	90C
rs4988235	*MCM6/LCTa*	G > A	-	2G	3G	1G	NA	2G	2G	66G
rs174546	*FADS1/FADS2*	T > C	+	NA	1C	NA	1T	NA	NA	44C,36T
rs7940244	*DHCR7&NADSYN1*	C > T	+	NA	4T,2C	1T	NA	4C,1T	NA	52C,38T
rs7944926	*DHCR7&NADSYN1*	G > A	+	NA	1A	NA	NA	2A	NA	41A,20G
rs12785878	*NADSYN1*	G > T	+	1T	NA	NA	NA	4G,1T	2T	48T,43G
rs1050152	*SLC22A4*	C > T	+	3C	NA	NA	NA	1C	1C	88C
rs272872	*SLC22A4*	G > A	-	1A	2G	NA	NA	6A	NA	68A
rs653178	*ATXN2*	C > T	-	1T	1T	NA	NA	3T	NA	52T
rs74771917	*FADS1-3*	C > T	+	1C	2C	NA	1C	NA	NA	91C
rs3168072	*FADS1-3*	A > T	+	NA	1A	NA	NA	1A	NA	62A
rs12577276	*FADS1-3*	A > G	+	3A	NA	NA	NA	4A	2A	87A
rs7115739	*FADS1-3*	G > T	+	NA	NA	NA	NA	3G	NA	72G
rs174602	*FADS1-3*	C > T	-	1T	NA	NA	2T	4T	NA	99T
rs174570	*FADS1-3*	C > T	+	NA	NA	NA	1T	7T	NA	31T,27C
rs2298080	*WARS2/TBX15*	G > A	+	2G	NA	NA	NA	6G,2A	2G	49A,29G

S8.5 Taste-perception related SNPs

The ability to taste phenylthiocarbamide (PTC) is closely linked with the ability to taste other compounds containing thiocyanate. This ability was linked to a gene called taste receptor 2 member 38 (*TAS2R38*) and is present among humans on two common haplotypes: taster (PAV haplotype) and non-taster (AVI haplotype). The latter is inherited as an autosomal recessive trait (OMIM 171200) [54]. In our study, the two individuals with sufficient genome coverage at the sites of interest (Hum2 and SF12) were carriers and were homozygous for the PAV haplotype (Table S8.6); thus, both had the ability to taste PTC [55]. Interestingly, the taster haplotype frequency in present-day Norway and Finland is in between PAV and AVI, and it has been suggested that the observed frequency arose as a result of drift rather than selection favoring specific haplotypes [56]. The distribution of PAV ad AVI haplotypes among WHG, EHG, CHG and PHG revealed that most ancient individuals were carriers of the PAV haplotype (see Table S8.6, S1 Table). Only two individuals were identified as carriers of AVI phenotype (Samara, Vestonice16), while further two were carriers of mixed haplotypes (Ajvide58, Kostenki14).

Due to the limited number of available hunter-gatherer genomes, this result should be taken with caution, however based on available data it seems all Western and Northern hunter-gatherers were carriers of PAV phenotype (with the exception of heterozygous Ajvide58), while AVI were only identified in individuals from central Europe.

Table S8.6 Bitter taste receptor TAS2R38 alleles

SNPID	gene	mutation	strand	Steigen	SF9	SF11	SBj	Hum2	Hum1	SF12
rs713598	*TAS2R38*	G > C	-	NA	NA	NA	1G	4C	NA	96C
rs1726866	*TAS2R38*	G > A	-	3G	NA	NA	NA	4A	NA	85A
rs10246939	*TAS2R38*	C > T	+	1T	3T	NA	NA	7T	NA	68T
rs4726481	*TAS2R38*	T > G	+	2T,1G	NA	NA	NA	9G	NA	52G
rs17162635	*TAS2R38*	T > A	+	2T	NA	NA	NA	4T	NA	63T
Haplotype	*TAS2R38*	-	-	NA	NA	NA	NA	PAV	NA	PAV

S8.6 Copy number variation at the AMY1 region

Copy number variation (CNV) in the human loci encoding for salivary amylase (*AMY1*), an enzyme involved in starch degradation, has been reported as an example of recent positive selection associated to diet changes [57]. Different from other great apes and archaic hominids [57,58] – which have 2 *AMY1* copies – the reference human genome has 3 copies of the *AMY1* gene: *AMY1A*, *AMY1B* and *AMY1C* (a total of six diploid copies). Each of these loci has been observed to expand into a higher copy number in present-day human populations, which might be explained by a starch-consumption adaptation upon the advent of agriculture [57]. The authors reported that modern human agriculturalist groups – known to have a higher starch consumption - present a higher *AMY1* gene copy number, in contrast to contemporary hunter gatherers – assumed to have a low-starch consumption - and exhibiting a lower copy number variation. Most present-day Europeans seem to have around 8-10 copies, with as low as two and as high as sixteen diploid copies. Other present-day African and non-African populations have similar distributions, however Asians present the widest range of copies in modern humans [58]. Recently it has been reported that the La Braña and Loschbour WHGs, the Motala12 SHG and the Stuttgart early farmer, had on average ~5, 13, 6 and 16 *AMY1* diploid gene copies, respectively [2,16]. While the *AMY1* diploid gene copy numbers in La Braña and the Motala12 individual agree with a low-starch hunter-gatherer diet, the prediction for the Loschbour WHG, falls well within the range of current European populations and farmers [2,57,58].

In order to continue to shed light on the distribution of *AMY1* gene copies in ancient hunter-gatherer groups, we aim to characterize the CNV in the SHGs from this study by predicting CNVs from the distribution of *de novo* mapped reads [16,59]. Illumina reads without adapter sequences were split into 36 bp reads, using the fastqutils module of the ngstils 0.5.9 suite [60]. Split reads were mapped against the human genome reference using mrFAST version 2.6.1.0 [59]. We used the hg19 version of the human reference masked for repeats detected with RepeatMasker (www.repeatmasker.org) and Tandem Repeat Finder [61]. Following mapping, mrCaNaVaR V 0.51 [59] was employed to calculate the mean read depth per base pair in 1 kb non-overlapping windows of non-repetitive sequence.

Using coordinates obtained from the UCSC browser for each of the *AMY1* genes (*AMY1A*, *AMY1B* and *AMY1C*), as well as the most upstream coordinate and most downstream positions, for *AMY1A* and *AMY1C* respectively, the average number of diploid *AMY1* gene copies was estimated for the SF12, Hum2 and Steigen individuals. CNV detection in the other individuals from this study was hindered by their low nuclear genome coverage. Given SF12's high nuclear genome coverage, and that the described methodology involves remapping raw sequencing reads, the following time-saving strategy was envisioned to analyze that individual's data. In brief, SF12 libraries were ranked by a high nuclear coverage and low mitochondrial contamination estimates. Following that order, libraries were grouped and merged together to generate three separate "test genomes" of an average coverage of 5x each. The protocol described above to call CNVs was performed for all three subsets separately as for the other individuals.

Table S8.7 Estimated copy numbers for the *AMY1* region.

Sample	*AMY1A*	*AMY1B*	*AMY1C*	Full region
SF12_1st_5x	10.3065	10.3256	9.53159	8.53405
SF12_2nd_5x	10.4606	10.5248	9.83696	8.38953
SF12_3rd_5x	8.08692	8.11287	7.59612	7.0647
Steigen	14.7399	14.8016	14.9535	12.2159
Hum2	12.8483	12.8709	12.8003	11.6797

The predicted copy number for each *AMY1* gene loci varied from 7.5 to ~15 for SF12, Hum2 and Steigen individuals. Given the high sequence similarity between the *AMY1* A, B and C genes and as mrFAST allows for each read to map against multiple sites, we expect most reads to map against all three genes and not only one of them. Therefore, we also use the depth estimate encompassing the whole region to report the number of *AMY1* copies per individual, which results in slightly smaller estimates. Our results, together with those from the LaBraña1, Loschbour, Motala12 and Stuttgart individuals, suggest that the amylase copy number expansion in modern-humans had occurred prior to the advent of agriculture in Europe. An observation also consistent with other recent studies on the worldwide distribution of the CNV [62].

S8.7 Metabolic syndrome related SNPs

Type 2 diabetes (T2D; OMIM 125853) is a complex disease with a variable world-wide distribution. Its development is preconditioned by a combination of genetic and environmental factors. A number of genetic risk scoring systems have been developed aming to facilitate T2D risk prediction. Following the approach of Lazaridis et al. [2] we use two scoring systems [63,64] to facilitate comparison of our ancient metabolic genotypes to modern non-diabetic genotype.

We were able to calculate phenotype prediction scores for two out of six investigated ancient SHG individuals. Note that results obtained for Hum2 should be treated with caution due to low coverage at a number of polymorphic sites. According to both systems, SF12's count based risk scores seem slightly lower than those of Hum2 with weighted genetic risk scores of 6.26 and 80.51 for SF12 and 8.16 and 79.14 for Hum2 according to the two different models respectively [63,64]. Both individuals

showed genetic risk scores (GRS) at the lower end of the score spectrum detected among modern-day Europeans. Even though the GRS prediction power is limited due to a number of other non-genetic factors influencing T2D, our results show that both SF12 and Hum2 had reduced MetS risk scores compared to both modern day Europeans [63,64], as well as the ancient forager and farmer Loschbour and Stuttgart individuals from continental Europe. We analyzed the genetic risk scores in another seven hunter-gatherers with >1x genome coverage. In all ancient individuals the genetic risk scores were either at the low (Motala12, Kotias, Kostenki14) or intermediate (Ajv58, LaBrana1, Bichon) levels compared to modern populations. The two ancient individuals with highest risk score, but still within the median range compared to modern populations, were Bichon and Loschbour or Ajv58, depending on the SNP scoring panel used.

Table S8.8 Method 2 results from Cornelis et al [64](217)

SNPID	gene	mutation	risk allele	strand	Hum2	SF12
rs564398	*CDKN2A/B*	T > C	T	-	3C	38C,40T
rs10010131	*WFS1*	G > A	G	+	2A,1G	42G,39A
rs4402960	*IGF2BP2*	G > T	T	+	3T,3G	45G
rs1801282	*PPARG*	C > G	C	+	2C,1G	28G,26C
rs5219	*KCNJ11*	C > T	T	+	2C	58C
rs1111875	*HHEX*	T > C	C	-	3C	34T,37C
rs13266634	*SLC30A8*	C > T	C	+	3C	71C
rs10811661	*CDKN2A/B*	C > T	T	+	8T	47T,46C
rs7756992	*CDKAL1*	G > A	G	+	8A	53A
rs12255372	*TCF7L2*	G > T	T	+	6G	95G
The Genetic Risk Score (GRS)					**9**	**7**
Weighted Genetic Risk Score					**8.16**	**6.26**

Table S8.9 Method 1 results from Meigs et al. [63](216)

SNPID	gene	mutation	risk allele	strand	Hum2	SF12
rs7901695	*TCF7L2*	C > T	C	+	2C,1T	55T
rs7903146	*TCF7L2*	T > C	T	+	6C	54C
rs1470579	*IGF2BP2*	C > A	C	+	2A	66A
rs10811661	*CDKN2A/B*	C > T	T	+	8T	46C,47T
rs864745	*JAZF1*	T > C	T	-	1T,1C	35C
rs5219	*KCNJ11*	C > T	T	+	2C	58C
rs5215	*KCNJ11*	T > C	C	+	1T	75T
rs12779790	*CDC123/CAMK1D*	A > G	G	+	8A	57A
rs7578597	*THADA*	T > C	T	+	4T	63T
rs7754840	*CDKAL1*	G > C	C	+	2G	41G
rs7961581	*TSPAN8/LGR5*	C > T	C	+	3T	47T
rs4607103	*ADAMTS9*	C > T	C	+	5C	69C
rs1111875	*HHEX*	T > C	C	-	3C	37C,34T
rs10923931	*NOTCH2*	G > T	T	+	6G	85G
rs13266634	*SLC30A8*	C > T	C	+	3C	71C
rs1153188	*DCD*	A > T	A	-	4A	34T,27A
rs1801282	*PPARG*	C > G	C	+	2C,1G	28G,29C
rs9472138	*VEGFA*	C > T	T	+	6C	34C,32T
rs10490072	*BCL11A*	T > C	T	+	4C*	57C
rs689	*INS*	A > T	A	-	2A	35A,32T
The Genetic Risk Score (GRS)					**14**	**14**
Weighted Genetic Risk Score					**79.14**	**80.51**

S8.8 Blood type

ABO blood group polymorphisms often referred to as classical genetic markers have been utilized in population studies ever since their discovery in the early 20th century. ABO blood groups consist of three major alleles (A, B and O) located on chromosome 9. Alleles A and B are codominant, while O is

recessive. Allele A has two major subgroups: A_1 and A_2. Therefore, there are six major possible ABO phenotypes encoded by various genotypes (listed in parenthesis): A_1 (A_1A_1, A_1A_2, A_1O), A_2 (A_2A_2, A_2O), A_1B (A_1B), A_2B (A_2B), B (BO or BB) and O(OO). The Rh (Rhesus) system is encoded by the RHD gene encoding for the D polypeptide. Rh+ denotes presence of the D antigen, while Rh- indicates absence of D [65].

By screening a number of SNPs associated with ABO polymorphisms we aimed at describing blood group variation in the investigated ancient individuals. There are numerous different SNPs in the AB0 gene encoding amino acid changes defining various ABO alleles. We have checked our individuals at a number of SNPs in the region, of which the following are the best known blood group defining variants: rs8176719 (G/-) defines blood group O in case of G deletion; rs7853989 (C;C or C;G) defines likely blood group B while (G;G) excludes B, rs8176746 (C;C) defines blood group A and (A;A) is characteristic of blood group B, rs8176747 (G;G) defines either allele A or O while rs590787 is a SNP defining Rhesus (Rh) with (C;C) defining Rh- and (C;T) and (T;T) defining Rh+ [66]. It was possible to make broad blood group inferences in Hum2 who was carrier of at least one O allele. However lack of coverage at rs590787 did not allow for evaluation of presence or absence of antigen D (Rh+/Rh-). The high coverage individual (SF12) was identified as a carrier of A0+.

Table S8.10 Blood defining SNPs

SNPID	gene	mutation	strand	Hum2	SF12
rs8176719	*ABO*	A > C	-	2-	25G,25-
rs1053878	*ABO*	G > A	-	NA	44G,36A
rs7853989	*ABO*	G > C	+	4G	102G
rs8176740	*ABO*	A > T	-	3A	83A
rs8176743	*ABO*	C > T	-	5C	99C
rs8176746	*ABO*	G > T	-	1G	84G
rs41302905	*ABO*	C > T	+	1C	80C
rs8176747	*ABO*	C > G	-	1C	79C
rs8176749	*ABO*	C > T	-	2C	81C
rs8176720	*ABO*	C > T	-	NA	50T
rs8176741	*ABO*	G > A	-	3G	89G
rs8176750	*ABO*	G > -	-	NA	55G
rs590787	*ABO*	A > C/G/T	-	NA	1A
**Blood type** ***(genotype)***				**NA** ***(O?)***	**A+** ***(A_1O)***

(http://www.ncbi.nlm.nih.gov/projects/gv/mhc/xslcgi.cgi?cmd=bgmut/systems_info&system=abo)

S8.9 Other phenotypic traits

We have tested all individuals for a number of SNPs associated with various additional phenotypic traits. SF12, SF9 and Hum1 had normal risk of hypertension carrying the low-risk allele A at the *AGT* gene (similar to 12 other hunter-gatherers) [67]. This was not the case for Steigen and Hum2 who were carriers of risk associated allele G and had increased risk of hypertension (the allele was also found in Motala1, Loschbour, Bichon, Karelia and Kotias individuals) (see S1 Table and Table S8.11).

KITLG conveys increased risk of testicular cancer, but also provides protection from sun damage causing UV protective tanning response in lighter skinned individuals [68]. The variant associated with UV protection was found in SF12 but not in SF11 or Hum2. The ancestral variant seems to be widespread among Scandinavian hunter-gatherers (identified in all Motala individuals), moderate in EHG and CHG individuals, but not particularly common within WHGs (See table S8.11).

Furthermore, SF9, SBj, Hum2 and SF12 as well as all other HG tested (with the exception of KO1), carried allelic variants at *ABCC1* gene associated with wet earwax, normal body odor and normal colostrum [69].

The lack of variant A alleles at the *ALDH2* (rs671) suggests absence of the Asian flush and reduced hangovers in SF12, SF9, SF11, SBj as well as all tested Hgs [70,71]. None of the tested individuals with sequence coverage at position rs1229984 were carriers of the A allele. If present, *ADH1B**47His conveys reduced risk for oral cancer and is associated with alcohol metabolism in East Asians. The mutation is closely linked to Asian flush, which is believed to protect from alcoholism [43,72]. It has been suggested that prevalence of derived *ADH1B**47His alleles could be explained by the increased alcohol consumption in connection to Neolithic rice domestication [43].

Hereditary haemochromatosis is a genetic disease caused by mutations in the *HFE* gene leading to increased accumulation of iron in body tissues, especially the liver, often resulting in cirrhosis [73]. The mutation causing haemochromatosis was hypothesized to have been disseminated with either Viking or Celtic expansions [74,75]. Steigen and Hum2 carried at least one copy of the risk-allele so they could be affected by a mild form of haemochromatosis. The distribution of alleles was very similar in all other tested hunter-gatherers with not a single identified carrier of the risk allele A at position rs1800562 and various mixed variants at rs1799945. Thus, the oldest so far identified European carrier of the C282Y mutation was a Bronze Age individual from Ireland [76].

Table S8.11 Allelic state of selected metabolic and physical conditions in all seven new SHGs.

SNPID	gene	mutation	strand	Steigen	SF9	SF11	SBj	Hum2	Hum1	SF12
rs699	*AGT*	G > A	-	1G	2A	NA	NA	2G,2A	2A	98A
rs4590952	*KITLG*	G > A	+	NA	NA	1A	NA	5A,1G	NA	50G
rs3827760	*EDAR*	A > G	-	1A	3A	NA	NA	8A	NA	88A
rs17822931	*ABCC1*	C > T	+	NA	4C	NA	1C	1C	NA	79C
rs671	*ALDH2*	G > A	+	NA	3G	1G	2G	NA	NA	100G
rs3811801	*ADH1Ba*	G > A	-	2G	NA	NA	1G	1G	3G	73G
rs1229984	*ADH1Bb*	C > T	-	NA	1C	NA	NA	3C	NA	57C
rs1800562	*HFE*	G > A	+	NA	1G	NA	NA	NA	NA	87G
rs1799945	*HFE*	C > G	+	2G	2C	NA	NA	2C,1G	NA	82C

S8.10 Immunological variants

A host-pathogen "arms race" has shaped the human genome as a result of selective pressures acting upon genes involved in host resistance and immune response. The Neolithic revolution has been regarded as one of the most important events during that process, due to the unprecedented exposure to zoonotic infections within relatively large new farmer settlements [77]. While several studies have suggested that a large number of the common modern infections in humans could be due to zoonotic post-Neolithic events [77,78], more recent findings suggest a more complex scenario.

It is reported that a Mesolithic hunter-gatherer from the Iberian Peninsula presented both derived and ancestral variants at SNPs influencing the susceptibility to infections, and within immune-related genes under positive selection in Europeans [16]. It was thus concluded that both pre- and post-Neolithic events contributed to shaping the immune system of present-day Europeans. However, if post-Neolithic events contributed or not to such adaptation remains unknown. The genomes of the seven SHG in this study, together with other Mesolithic and Paleolithic hunter-gatherers across Europe, provide an unprecedented sample size of 27 pre-Neolithic hunter-gatherers (Table S8.1 and S8.12) to investigate this topic.

We built an empirical distribution of derived allele frequency differences between present-day Europeans and HGs for all genic SNPs throughout the genome. We then compared this genome-wide distribution to the allele frequency differences for 67 SNPs from genes which show patterns of positive selection in Europeans and which include polymorphisms that influence susceptibility to infections in Europeans (Table S8.12) [16]. If these immune response sites are among the regions of the genome that have changed the most, then exposure to previously unknown environments and pathogens would have shaped the immune system since Mesolithic times.

In order to build the empirical distribution, derived allele frequencies were estimated for all 16,668,219 SNPs (as annotated in Ensemble 75) in all present-day Europeans of the 1000 genomes project (1KGP) data [79]. Next, we also estimated the derived allele frequency at each of these positions for the 27 screened European HGs as described above (S8.1). We restricted our analysis to sites were the derived allele was variable in both populations. Singletons in present-day Europeans, and singletons in the ancient population with data for less than four HGs were excluded as well. This filter avoids potential sequencing errors or unfiltered DNA damage (see beginning of S8 Text) to be considered as low frequency variants. Our empirical distribution of allele frequency differences between the two groups was derived from 5,488,574 sites after filtering. However, since HG sequencing mainly came from low-coverage shotgun or capture data, large proportions of missing data adds noise to the derived allele frequency estimations for ancient populations. In order to obtain somewhat reliable allele frequency estimates, we restricted our analysis to SNPs that had sequence data from at least 25% (7/27) of the HGs.

Table S8.12 shows the results for all SNPs in this analysis. There appears to be an enrichment of the immunological SNPs among the sites that have changed the most after the Mesolithic period. Nine out of 67 immune SNPs (13.4%) were in the top 5% of the empirical distribution when restricting to sites which were covered in at least 25% of the HGs. Seven out of 42 sites (16.7%) were among the top 5% when restricting to sites covered in at least 50% of the HGs. Figure S8.1 was produced with sites that had information from at least 25% of all HGs.

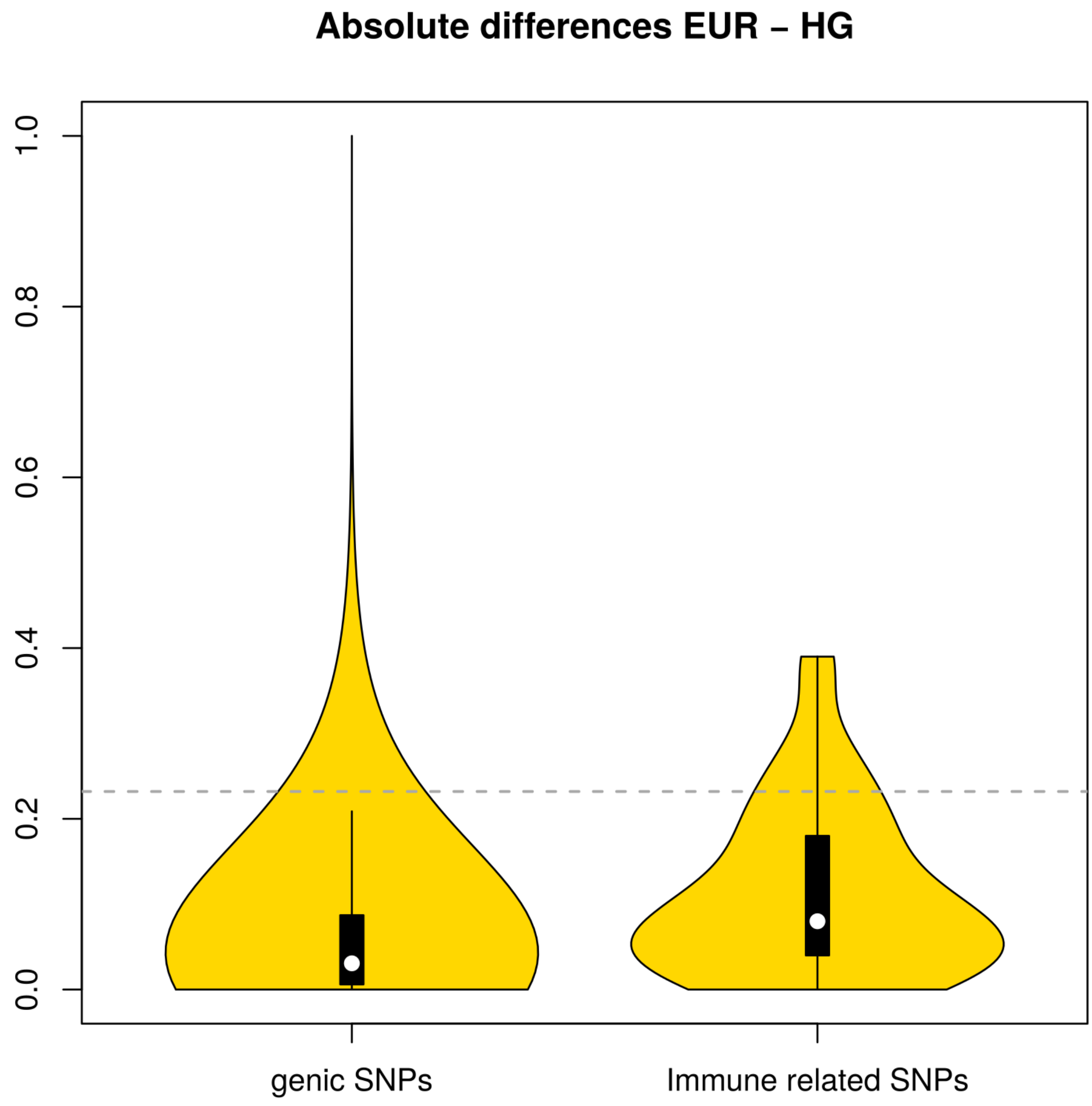

Figure S8.1 Distribution of absolute allele frequency differences between modern Europeans and Mesolithic Europeans for all genic sites covered in at least 25% of the Mesolithic individuals. The dashed horizontal line represents the top 5% of all SNPs.

Table S8.12 Comparison of the allele frequency differences between immunological SNPs and a genome-wide distribution for all genic SNPs.

				Derived allele frequencies			Percentile of the genome-wide distribution conditional on the proportion of HGs covered		
**SNP ID**	**Gene**	**Derived allele**	**% of covered HGs**	**EUR**	**HG**	**Absolute derived allele frequency difference**	**> 25 % HG w/ data**	**> 50% HG w/ data**	**> 75% HG w/ data**
rs497116	*CASP12*	A	44.4444	0.999	1.000	0.001	3.064	NA	NA
rs2039381	*IFNE*	A	44.4444	0.003	0.000	0.003	12.836	NA	NA
rs9302752	*NOD2*	T	85.1852	0.255	0.258	0.003	17.541	17.926	10.625
rs4986790	*TLR4*	G	81.4815	0.057	0.050	0.007	26.389	26.951	16.591
rs10982385	*TNFSF15*	G	66.6667	0.573	0.580	0.007	28.252	28.84	NA
rs1800872	*IL-10*	T	88.8889	0.240	0.250	0.010	32.885	33.554	21.468
rs1024611	*CCL2/MCP-1*	G	62.963	0.316	0.304	0.012	34.328	35.039	NA
rs5743315	*TLR3*	A	37.037	0.012	0.000	0.012	34.381	NA	NA
rs2015070	*CCL18*	T	66.6667	0.096	0.083	0.013	36.369	37.111	NA
rs2066842	*NOD2*	T	74.0741	0.247	0.260	0.014	36.514	37.272	NA
rs1800451	*MBL2*	T	77.7778	0.012	0.033	0.021	43.669	44.667	31.852
rs2015086	*CCL18*	A	85.1852	0.860	0.838	0.022	43.729	44.726	32.015
rs61752945	*RIG-1*	T	40.7407	0.009	0.031	0.022	44.421	NA	NA
rs5743708	*TLR2*	A	88.8889	0.024	0.000	0.024	45.441	46.519	33.989
rs4251545	*IRAK4*	A	74.0741	0.087	0.113	0.026	47.339	48.444	NA
rs2569190	*CD14*	A	77.7778	0.486	0.452	0.034	52.3	53.591	43.228
rs17217280	*RIG-1*	T	37.037	0.136	0.094	0.042	57.226	NA	NA
rs12212067	*FOXO3A*	T	48.1481	0.872	0.917	0.045	58.924	NA	NA
rs30461	*IL-29*	A	88.8889	0.888	0.934	0.047	59.871	61.951	50.358
rs10930046	*IFIH1/MDA5*	T	44.4444	0.988	0.941	0.047	60.179	NA	NA
rs3802814	*Mal/TIRAP*	A	55.5556	0.172	0.125	0.047	60.213	62.277	NA
rs2069727	*IFNG*	C	85.1852	0.463	0.414	0.049	61.437	63.584	51.74
rs4986791	*TLR4*	T	74.0741	0.058	0.107	0.049	61.6	63.755	NA
rs2779249	*NOS2A*	A	51.8519	0.325	0.275	0.050	61.977	64.098	NA
rs14304	*CCL18*	T	62.963	0.318	0.370	0.051	62.751	64.871	NA
rs5743836	*TLR9*	A	44.4444	0.869	0.813	0.056	65.004	NA	NA
rs8078340	*NOS2A*	A	40.7407	0.130	0.071	0.059	66.014	NA	NA
rs361525	*TNF*	A	81.4815	0.064	0.000	0.064	67.709	69.389	59.441
rs179008	*TLR7*	T	29.6296	0.231	0.167	0.064	67.969	NA	NA
rs10114470	*TNFSF15*	T	81.4815	0.325	0.391	0.066	68.398	70.065	60.443
rs16910526	*Dectin-1/CLEC7A*	C	40.7407	0.071	0.000	0.071	70.047	NA	NA
rs4574921	*TNFSF15*	C	44.4444	0.266	0.344	0.077	72.168	NA	NA
rs2274894	*NOS2A*	T	55.5556	0.396	0.476	0.081	73.137	74.867	NA

rs3753344	*TNFRSF18*	A	37.037	0.052	0.133	0.082	73.435	NA	NA
rs1873613	*LRRK2*	C	44.4444	0.695	0.778	0.083	73.81	NA	NA
rs8177374	*Mal/TIRAP*	T	55.5556	0.186	0.275	0.089	75.571	77.395	NA
rs7137054	*SOCS2*	G	48.1481	0.157	0.250	0.093	76.61	NA	NA
rs1891467	*TGFB2*	A	85.1852	0.761	0.855	0.093	76.757	78.636	71.363
rs1800629	*TNF*	A	85.1852	0.134	0.029	0.105	79.595	81.444	74.994
rs10745657	*SOCS2*	G	48.1481	0.506	0.611	0.105	79.639	NA	NA
rs2305619	*PTX3*	G	62.963	0.544	0.438	0.106	79.916	81.748	NA
rs333	*CCR5*	-	40.7407	0.110	0.000	0.110	80.798	NA	NA
rs42490	*RIPK2*	A	81.4815	0.611	0.722	0.111	80.929	82.703	76.786
rs7215373	*NOS2A*	T	44.4444	0.532	0.647	0.115	81.798	NA	NA
rs2228428	*CCR4*	T	81.4815	0.314	0.191	0.123	83.216	84.899	79.875
rs8057341	*NOD2*	A	85.1852	0.295	0.167	0.129	84.203	85.857	81.199
rs3750920	*TOLLIP*	T	44.4444	0.457	0.313	0.145	86.742	NA	NA
rs11754268	*IFNGR1*	T	37.037	0.231	0.083	0.147	87.098	NA	NA
rs3775291	*TLR3*	T	81.4815	0.324	0.483	0.159	88.605	90.109	87.081
rs5030737	*MBL2*	A	70.3704	0.060	0.234	0.175	90.444	91.843	NA
rs900	*TGFB2*	T	44.4444	0.280	0.094	0.187	91.601	NA	NA
rs3802813	*Mal/TIRAP*	A	77.7778	0.042	0.230	0.188	91.739	93.053	91.301
rs3764880	*TLR8*	A	70.3704	0.731	0.920	0.189	91.833	93.14	NA
rs3135499	*NOD2*	A	85.1852	0.565	0.375	0.190	91.887	93.19	91.497
rs2043055	*IL-18BP*	G	70.3704	0.372	0.180	0.192	92.085	93.378	NA
rs4804803	*DC-SIGN*	A	44.4444	0.784	0.588	0.196	92.464	NA	NA
rs6478108	*TNFSF15*	C	88.8889	0.336	0.547	0.211	93.619	94.788	93.638
rs4833095	*TLR1*	T	51.8519	0.720	0.500	0.220	94.225	95.339	NA
rs2333227	*MPO*	T	44.4444	0.238	0.000	0.238	95.317	NA	NA
rs2074158	*LGP2*	C	70.3704	0.178	0.417	0.239	95.386	96.384	NA
rs5743899	*TOLLIP*	T	62.963	0.789	0.524	0.265	96.648	97.488	NA
rs4077515	*CARD9*	T	66.6667	0.397	0.667	0.270	96.825	97.64	NA
rs11096957	*TLR10*	G	74.0741	0.398	0.672	0.275	97.01	97.798	NA
rs4073	*IL-8*	T	40.7407	0.579	0.300	0.279	97.143	NA	NA
rs5743810	*TLR6*	A	66.6667	0.409	0.042	0.367	99.088	99.474	NA
rs2430561	*IFNG*	A	29.6296	0.462	0.083	0.379	99.225	NA	NA
rs3184504	*SH2B3*	T	81.4815	0.464	0.076	0.388	99.316	99.636	99.693

References

1. Fu Q, Posth C, Hajdinjak M, Petr M, Mallick S, Fernandes D, et al. The genetic history of Ice Age Europe. Nature. 2016;534: 200–5. doi:10.1038/nature17993

2. Lazaridis I, Patterson N, Mittnik A, Renaud G, Mallick S, Kirsanow K, et al. Ancient human genomes suggest three ancestral populations for present-day Europeans. Nature. 2014;513: 409–413. doi:10.1038/nature13673

3. Haak W, Lazaridis I, Patterson N, Rohland N, Mallick S, Llamas B, et al. Massive migration from the steppe was a source for Indo-European languages in Europe. Nature. 2015; doi:10.1038/nature14317

4. Ginolhac A, Rasmussen M, Gilbert MTP, Willerslev E, Orlando L. mapDamage: Testing for damage patterns in ancient DNA sequences. Bioinformatics. 2011;27: 2153–2155. doi:10.1093/bioinformatics/btr347

5. Jónsson H, Ginolhac A, Schubert M, Johnson PL, Orlando L. mapDamage2. 0: fast approximate Bayesian estimates of ancient DNA damage parameters. Bioinformatics. 2013;29: 1682–1684.

6. Li H, Handsaker B, Wysoker A, Fennell T, Ruan J, Homer N, et al. The Sequence Alignment/Map format and SAMtools. Bioinformatics. 2009;25: 2078–2079. doi:10.1093/bioinformatics/btp352

7. Walsh S, Lindenbergh A, Zuniga SB, Sijen T, De Knijff P, Kayser M, et al. Developmental validation of the IrisPlex system: Determination of blue and brown iris colour for forensic intelligence. Forensic Science International: Genetics. 2011;5: 464–471. doi:10.1016/j.fsigen.2010.09.008

8. Walsh S, Wollstein A, Liu F, Chakravarthy U, Rahu M, Seland JH, et al. DNA-based eye colour prediction across Europe with the IrisPlex system. Forensic Science International: Genetics. 2012;6: 330–340. doi:10.1016/j.fsigen.2011.07.009

9. Walsh S, Liu F, Wollstein A, Kovatsi L, Ralf A, Kosiniak-Kamysz A, et al. The HIrisPlex system for simultaneous prediction of hair and eye colour from DNA. Forensic Science International: Genetics. 2013;7: 98–115. doi:10.1016/j.fsigen.2012.07.005

10. Spichenok O, Budimlija ZM, Mitchell AA, Jenny A, Kovacevic L, Marjanovic D, et al. Prediction of eye and skin color in diverse populations using seven SNPs. Forensic Science International: Genetics. 2011;5: 472–478. doi:10.1016/j.fsigen.2010.10.005

11. Branicki W, Liu F, Van Duijn K, Draus-Barini J, Pośpiech E, Walsh S, et al. Model-based prediction of human hair color using DNA variants. Human Genetics. 2011;129: 443–454. doi:10.1007/s00439-010-0939-8

12. Hart KL, Kimura SL, Mushailov V, Budimlija ZM, Prinz M, Wurmbach E. Improved eye- and skin-color prediction based on 8 SNPs. Croatian medical journal. 2013;54: 248–56. doi:10.3325/cmj.2013.54.248

13. Pneuman A, Budimlija ZM, Caragine T, Prinz M, Wurmbach E. Verification of eye and skin color predictors in various populations. Legal Medicine. 2012;14: 78–83. doi:10.1016/j.legalmed.2011.12.005

14. Walsh S, Chaitanya L, Clarisse L, Wirken L, Draus-Barini J, Kovatsi L, et al. Developmental validation of the HIrisPlex system: DNA-based eye and hair colour prediction for forensic and

anthropological usage. Forensic Science International: Genetics. 2014;9: 150–161. doi:10.1016/j.fsigen.2013.12.006

15. Jones ER, Gonzalez-Fortes G, Connell S, Siska V, Eriksson A, Martiniano R, et al. Upper Palaeolithic genomes reveal deep roots of modern Eurasians. Nature communications. 2015;6: 8912. doi:10.1038/ncomms9912

16. Olalde I, Allentoft ME, Sánchez-Quinto F, Santpere G, Chiang CWK, DeGiorgio M, et al. Derived immune and ancestral pigmentation alleles in a 7,000-year-old Mesolithic European. Nature. 2014;507: 225–228. doi:10.1038/nature12960

17. Hofmanová. Early farmers from across Europe directly descended from Neolithic Aegeans. PNAS. 2016; 201523951. doi:10.1073/pnas.1523951113

18. Lamason RL, Mohideen M-APK, Mest JR, Wong AC, Norton HL, Aros MC, et al. SLC24A5, a putative cation exchanger, affects pigmentation in zebrafish and humans. Science. 2005;310: 1782–1786. doi:10.1126/science.1116238

19. Stokowski RP, Pant PVK, Dadd T, Fereday A, Hinds D a, Jarman C, et al. A genomewide association study of skin pigmentation in a South Asian population. American journal of human genetics. 2007;81: 1119–1132. doi:10.1086/522235

20. Norton HL, Kittles RA, Parra E, McKeigue P, Mao X, Cheng K, et al. Genetic evidence for the convergent evolution of light skin in Europeans and East Asians. Molecular Biology and Evolution. 2007;24: 710–722. doi:10.1093/molbev/msl203

21. Beleza S, Santos AM, McEvoy B, Alves I, Martinho C, Cameron E, et al. The timing of pigmentation lightening in Europeans. Molecular Biology and Evolution. 2013;30: 24–35. doi:10.1093/molbev/mss207

22. Soejima M, Tachida H, Ishida T, Sano A, Koda Y. Evidence for recent positive selection at the human AIM1 locus in a European population. Molecular Biology and Evolution. 2006;23: 179–188. doi:10.1093/molbev/msj018

23. Grossman SR, Andersen KG, Shlyakhter I, Tabrizi S, Winnicki S, Yen A, et al. Identifying recent adaptations in large-scale genomic data. Cell. 2013;152: 703–713. doi:10.1016/j.cell.2013.01.035

24. Donnelly MP, Paschou P, Grigorenko E, Gurwitz D, Barta C, Lu RB, et al. A global view of the OCA2-HERC2 region and pigmentation. Human Genetics. 2012;131: 683–696. doi:10.1007/s00439-011-1110-x

25. Marcus JH, Novembre J. Visualizing the Geography of Genetic Variants. Bioinformatics (Oxford, England). 2016; doi:10.1093/bioinformatics/btw643

26. Eiberg H, Troelsen J, Nielsen M, Mikkelsen A, Mengel-From J, Kjaer KW, et al. Blue eye color in humans may be caused by a perfectly associated founder mutation in a regulatory element located within the HERC2 gene inhibiting OCA2 expression. Human Genetics. 2008;123: 177–187. doi:10.1007/s00439-007-0460-x

27. Gamba C, Jones ER, Teasdale MD, McLaughlin RL, Gonzalez-Fortes G, Mattiangeli V, et al. Genome flux and stasis in a five millennium transect of European prehistory. Nature Communications. 2014;5: 5257. doi:10.1038/ncomms6257

28. Mathieson I, Lazaridis I, Rohland N, Mallick S, Patterson N, Roodenberg SA, et al. Genome-wide patterns of selection in 230 ancient Eurasians. Nature. 2015;528: 499–503. doi:10.1038/nature16152

29. Skoglund P, Malmstrom H, Omrak A, Raghavan M, Valdiosera C, Gunther T, et al. Genomic Diversity and Admixture Differs for Stone-Age Scandinavian Foragers and Farmers. Science. 2014;344: 747–750. doi:10.1126/science.1253448

30. Kılınç GM, Omrak A, Özer F, Günther T, Büyükkarakaya AM, Bıçakçı E, et al. The Demographic Development of the First Farmers in Anatolia. Current Biology. 2016;26: 2659–2666. doi:10.1016/j.cub.2016.07.057

31. Nakagome S, Alkorta-Aranburu G, Amato R, Howie B, Peter BM, Hudson RR, et al. Estimating the ages of selection signals from different epochs in human history. Molecular biology and evolution. 2015;33: 657–669.

32. Basu Mallick C, Iliescu FM, Möls M, Hill S, Tamang R, Chaubey G, et al. The Light Skin Allele of SLC24A5 in South Asians and Europeans Shares Identity by Descent. PLoS Genetics. 2013;9. doi:10.1371/journal.pgen.1003912

33. Yuasa I, Umetsu K, Harihara S, Kido A, Miyoshi A, Saitou N, et al. Distribution of the F374 allele of the SLC45A2 (MATP) gene and founder-haplotype analysis. Annals of Human Genetics. 2006;70: 802–811. doi:10.1111/j.1469-1809.2006.00261.x

34. Canfield V a, Berg A, Peckins S, Wentzel SM, Ang KC, Oppenheimer S, et al. Molecular phylogeography of a human autosomal skin color locus under natural selection. G3 (Bethesda, Md). 2013;3: 2059–67. doi:10.1534/g3.113.007484

35. Adhikari K, Fontanil T, Cal S, Mendoza-Revilla J, Fuentes-Guajardo M, Chacón-Duque J-C, et al. A genome-wide association scan in admixed Latin Americans identifies loci influencing facial and scalp hair features. Nature communications. 2016;7: 10815.

36. Kamberov YG, Wang S, Tan J, Gerbault P, Wark A, Tan L, et al. Modeling recent human evolution in mice by expression of a selected EDAR variant. Cell. 2013;152: 691–702. doi:10.1016/j.cell.2013.01.016

37. Medland SE, Nyholt DR, Painter JN, McEvoy BP, McRae AF, Zhu G, et al. Common Variants in the Trichohyalin Gene Are Associated with Straight Hair in Europeans. American Journal of Human Genetics. 2009;85: 750–755. doi:10.1016/j.ajhg.2009.10.009

38. Li R, Brockschmidt FF, Kiefer AK, Stefansson H, Nyholt DR, Song K, et al. Six novel susceptibility loci for early-onset androgenetic alopecia and their unexpected association with common diseases. PLoS Genetics. 2012;8. doi:10.1371/journal.pgen.1002746

39. Jobling M, Hollox E, Hurles M, Kivisild T, Tyler-Smith C. Human Evolutionary Genetics [Internet]. 1989. doi:10.1017/CBO9781107415324.004

40. Hein DW. Molecular genetics and function of NAT1 and NAT2: Role in aromatic amine metabolism and carcinogenesis. Mutation Research - Fundamental and Molecular Mechanisms of Mutagenesis. 2002;506–507: 65–77. doi:10.1016/S0027-5107(02)00153-7

41. Sabbagh A, Darlu P, Crouau-Roy B, Poloni ES. Arylamine N-Acetyltransferase 2 (NAT2) Genetic Diversity and Traditional Subsistence: A Worldwide Population Survey. PLoS ONE. 2011;6. doi:10.1371/journal.pone.0018507

42. Patin E, Barreiro LB, Sabeti PC, Austerlitz F, Luca F, Sajantila A, et al. Deciphering the ancient and complex evolutionary history of human arylamine N-acetyltransferase genes. American Journal of Human Genetics, The. 2006;78: 423–436. doi:10.1086/500614

43. Peng Y, Shi H, Qi X, Xiao C, Zhong H, Ma RZ, et al. The ADH1B Arg47His polymorphism in east Asian populations and expansion of rice domestication in history. BMC evolutionary biology. 2010;10: 15. doi:10.1186/1471-2148-10-15

44. Kuznetsov IB, Mcduffie M, Moslehi R. A web server for inferring the human N-acetyltransferase-2 (NAT2) enzymatic phenotype from NAT2 genotype. Bioinformatics. 2009;25: 1185–1186. doi:10.1093/bioinformatics/btp121

45. He YJ, Shapero MH, Mcleod HL. Novel tagging SNP rs1495741 and 2-SNPs (rs1041983 and rs1801280) yield high prediction of NAT2 genotype in HapMap samples. Pharmacogenetics and Genomics. 2012;22: 322–324. doi:10.1097/FPC.0b013e3283510a51

46. Selinski S, Blaszkewicz M, Lehmann M-L, Ovsiannikov D, Moormann O, Guballa C, et al. Genotyping NAT2 with only two SNPs (rs1041983 and rs1801280) outperforms the tagging SNP rs1495741 and is equivalent to the conventional 7-SNP NAT2 genotype. Pharmacogenetics and genomics. 2011;21: 673–8. doi:10.1097/FPC.0b013e3283493a23

47. Racimo F, Sankararaman S, Nielsen R, Huerta-Sánchez E. Evidence for archaic adaptive introgression in humans. Nature reviews Genetics. 2015;16: 359–371. doi:10.1038/nrg3936

48. Wang TJ, Zhang F, Richards JB, Kestenbaum B, Van Meurs JB, Berry D, et al. Common genetic determinants of vitamin D insufficiency: A genome-wide association study. The Lancet. 2010;376: 180–188. doi:10.1016/S0140-6736(10)60588-0

49. Teslovich TM, Musunuru K, Smith AV, Edmondson AC, Stylianou IM, Koseki M, et al. Biological, clinical and population relevance of 95 loci for blood lipids. Nature. 2010;466: 707–13. doi:10.1038/nature09270

50. Mathias RA, Sergeant S, Ruczinski I, Torgerson DG, Hugenschmidt CE, Kubala M, et al. The impact of FADS genetic variants on $ømega$6 polyunsaturated fatty acid metabolism in African Americans. BMC genetics. 2011;12: 50. doi:10.1186/1471-2156-12-50

51. Fumagalli M, Moltke I, Grarup N, Racimo F, Bjerregaard P, Jørgensen ME, et al. Greenlandic Inuit show genetic signatures of diet and climate adaptation. Science (New York, NY). 2015;349: 1343–7. doi:10.1126/science.aab2319

52. Tosi F, Sartori F, Guarini P, Olivieri O, Martinelli N. Delta-5 and delta-6 desaturases: Crucial enzymes in polyunsaturated fatty acid-related pathways with pleiotropic influences in health and disease. 2014. doi:10.1007/978-3-319-7320-0_7

53. Allentoft ME, Sikora M, Sjögren K-G, Rasmussen S, Rasmussen M, Stenderup J, et al. Population genomics of Bronze Age Eurasia. Nature. 2015;522: 167–172. doi:10.1038/nature14507

54. Jobling M, Hollox E, Hurles M, Kivisild T, Tyler-Smith C. Human Evolutionary Genetics, Second Edition. Garland Science; 2013.

55. Wooding S, Kim U-K, Bamshad MJ, Larsen J, Jorde LB, Drayna D. Natural selection and molecular evolution in PTC, a bitter-taste receptor gene. American journal of human genetics. 2004;74: 637–46. doi:10.1086/383092

56. Risso DS, Mezzavilla M, Pagani L, Robino A, Morini G, Tofanelli S, et al. Global diversity in the TAS2R38 bitter taste receptor: revisiting a classic evolutionary PROPosal. Scientific reports. 2016;6: 25506. doi:10.1038/srep25506

57. Perry GH, Dominy NJ, Claw KG, Lee AS, Fiegler H, Redon R, et al. Diet and the evolution of human amylase gene copy number variation. Nature genetics. 2007;39: 1256–1260.

58. Prüfer K, Racimo F, Patterson N, Jay F, Sankararaman S, Sawyer S, et al. The complete genome sequence of a Neanderthal from the Altai Mountains. Nature. 2014;505: 43–9. doi:10.1038/nature12886

59. Alkan C, Kidd JM, Marques-Bonet T, Aksay G, Antonacci F, Hormozdiari F, et al. Personalized copy number and segmental duplication maps using next-generation sequencing. Nature genetics. 2009;41: 1061–1067. doi:10.1038/ng.437

60. Breese MR, Liu Y. NGSUtils: A software suite for analyzing and manipulating next-generation sequencing datasets. Bioinformatics. 2013;29: 494–496. doi:10.1093/bioinformatics/bts731

61. Benson G. Tandem repeats finder: A program to analyze DNA sequences. Nucleic Acids Research. 1999;27: 573–580. doi:10.1093/nar/27.2.573

62. Inchley CE, Larbey CD, Shwan NA, Pagani L, Saag L, Antão T, et al. Selective sweep on human amylase genes postdates the split with Neanderthals. Scientific reports. 2016;6: 37198.

63. Meigs JB, Shrader P, Sullivan LM, McAteer JB, Fox CS, Dupuis J, et al. Genotype score in addition to common risk factors for prediction of type 2 diabetes. The New England journal of medicine. 2008;359: 2208–2219. doi:10.1056/NEJMoa0804742

64. Cornelis MC, Qi L, Zhang C, Kraft P, Manson JA, Cai T, et al. Joint effects of common genetic variants on the risk for type 2 diabetes in U.S. men and women of European ancestry. Annals of Internal Medicine. 2009;150: 541–550. doi:10.1059/0003-4819-150-8-200904210-00008

65. Mielke JH, Konigsberg LW, Relethford J. Human biological variation. Oxford University Press; 2011.

66. Melzer D, Perry JRB, Hernandez D, Corsi A-M, Stevens K, Rafferty I, et al. A genome-wide association study identifies protein quantitative trait loci (pQTLs). PLoS genetics. 2008;4: e1000072. doi:10.1371/journal.pgen.1000072

67. Mondry A, Loh M, Liu P, Zhu A-L, Nagel M. Polymorphisms of the insertion / deletion ACE and M235T AGT genes and hypertension: surprising new findings and meta-analysis of data. BMC Nephrol. 2005;6: 1. doi:10.1186/1471-2369-6-1

68. Zeron-Medina J, Wang X, Repapi E, Campbell MR, Su D, Castro-Giner F, et al. XA polymorphic p53 response element in KIT ligand influences cancer risk and has undergone natural selection. Cell. 2013;155. doi:10.1016/j.cell.2013.09.017

69. Yoshiura K, Kinoshita A, Ishida T, Ninokata A, Ishikawa T, Kaname T, et al. A SNP in the ABCC11 gene is the determinant of human earwax type. Nat Genet. 2006;38: 324–330. doi:10.1038/ng1733

70. Crabb DW, Edenberg HJ, Bosron WF, Li TK. Genotypes for aldehyde dehydrogenase deficiency and alcohol sensitivity. The inactive ALDH2(2) allele is dominant. J Clin Invest. 1989;83: 314–316. doi:10.1172/JCI113875

71. Quillen EE, Chen X-D, Almasy L, Yang F, He H, Li X, et al. ALDH2 is associated to alcohol dependence and is the major genetic determinant of “daily maximum drinks” in a GWAS study of an isolated rural Chinese sample. Am J Med Genet B Neuropsychiatr Genet. 2014;165B: 103–110. doi:10.1002/ajmg.b.32213

72. Brooks PJ, Enoch M-A, Goldman D, Li T-K, Yokoyama A. The alcohol flushing response: an unrecognized risk factor for esophageal cancer from alcohol consumption. PLoS Med. 2009;6: e50. doi:10.1371/journal.pmed.1000050

73. Asberg a, Hveem K, Thorstensen K, Ellekjter E, Kannelønning K, Fjøsne U, et al. Screening for hemochromatosis: high prevalence and low morbidity in an unselected population of 65,238 persons. Scandinavian journal of gastroenterology. 2001;36: 1108–1115. doi:10.1080/003655201750422747

74. Whittington CA. Was the C282Y mutation an Irish Gaelic mutation that the Vikings help disseminate? Medical Hypotheses. 2006;67: 1270–1273. doi:10.1016/j.mehy.2006.06.013

75. Milman N, Pedersen P. Evidence that the Cys282Tyr mutation of the HFE gene originated from a population in Southern Scandinavia and spread with the Vikings. Clinical Genetics. 2003;64: 36–47. doi:10.1034/j.1399-0004.2003.00083.x

76. Cassidy LM, Martiniano R, Murphy EM, Teasdale MD, Mallory J, Hartwell B, et al. Neolithic and Bronze Age migration to Ireland and establishment of the insular Atlantic genome. Proceedings of the National Academy of Sciences. 2015; 1–6. doi:10.1073/pnas.1518445113

77. Wolfe ND, Dunavan CP, Diamond J. Origins of major human infectious diseases. Nature. 2007;447: 279–283. doi:10.1038/nature05775

78. Barreiro LB, Quintana-Murci L. From evolutionary genetics to human immunology: how selection shapes host defence genes. Nature Reviews Genetics. 2010;11: 17–30.

79. Auton A, Abecasis GR, Altshuler DM, Durbin RM, Abecasis GR, Bentley DR, et al. A global reference for human genetic variation. Nature. 2015;526: 68–74. doi:10.1038/nature15393
