# Supplementary material for: Population genomics of Mesolithic Scandinavia: Investigating early postglacial migration routes and high-latitude adaptation
Source: PLoS Biol. 2018 Jan 9;16(1):e2003703. doi: 10.1371/journal.pbio.2003703 (PMC5760011; doi:10.1371/journal.pbio.2003703)
Supplement: S9 Text — (PDF) [file pbio.2003703.s014.pdf]

S9 Adaptation to high-latitude climates

S9.1 Genome-wide scan for patterns of adaptation

This study presents the largest number of Mesolithic Scandinavians (to date) that have had their genomes sequenced. These individuals were among the first pioneering inhabitants of Scandinavia and northern Europe. While genetic variation of the Mesolithic populations falls outside the modern-European genetic variation, it is known that modern-day Europeans trace some ancestry to these groups [1,2]. Assuming that Mesolithic as well as modern-day northern Europeans were adapted to similar climatic conditions, possibly by sharing some genetic material (i.e., continuity) as previously demonstrated [1,3], we investigated if certain alleles or gene-regions show a long term continuity in the region. Signals of such allele/gene-region continuity will be informative of local adaptation, possibly linked to the environment at northern latitudes. A strong selective pressure in high-latitude regions is cold temperature. The response to cold stress is cardiovascular, metabolic and endocrinological while physiological adaptation to cold climates is mainly insulative or metabolic [4]. A recently detected example of adaptation to arctic climates is the gene cluster for fatty acid desaturase enzymes (FADS), in the Greenlandic Inuit population, which modulate fatty acid composition [5].

We scanned the genomes for SNPs with similar allele frequencies in Mesolithic and modern-day northern Europeans, and contrast it to a modern-day population from southern latitudes using D_{sel}. Outliers detected using this approach appear to have functional relevance as the upper end of the distribution of D_{sel} values is enriched with SNPs at conserved sites (measured by GERP score > 3; [6]) (Figure S9.1).

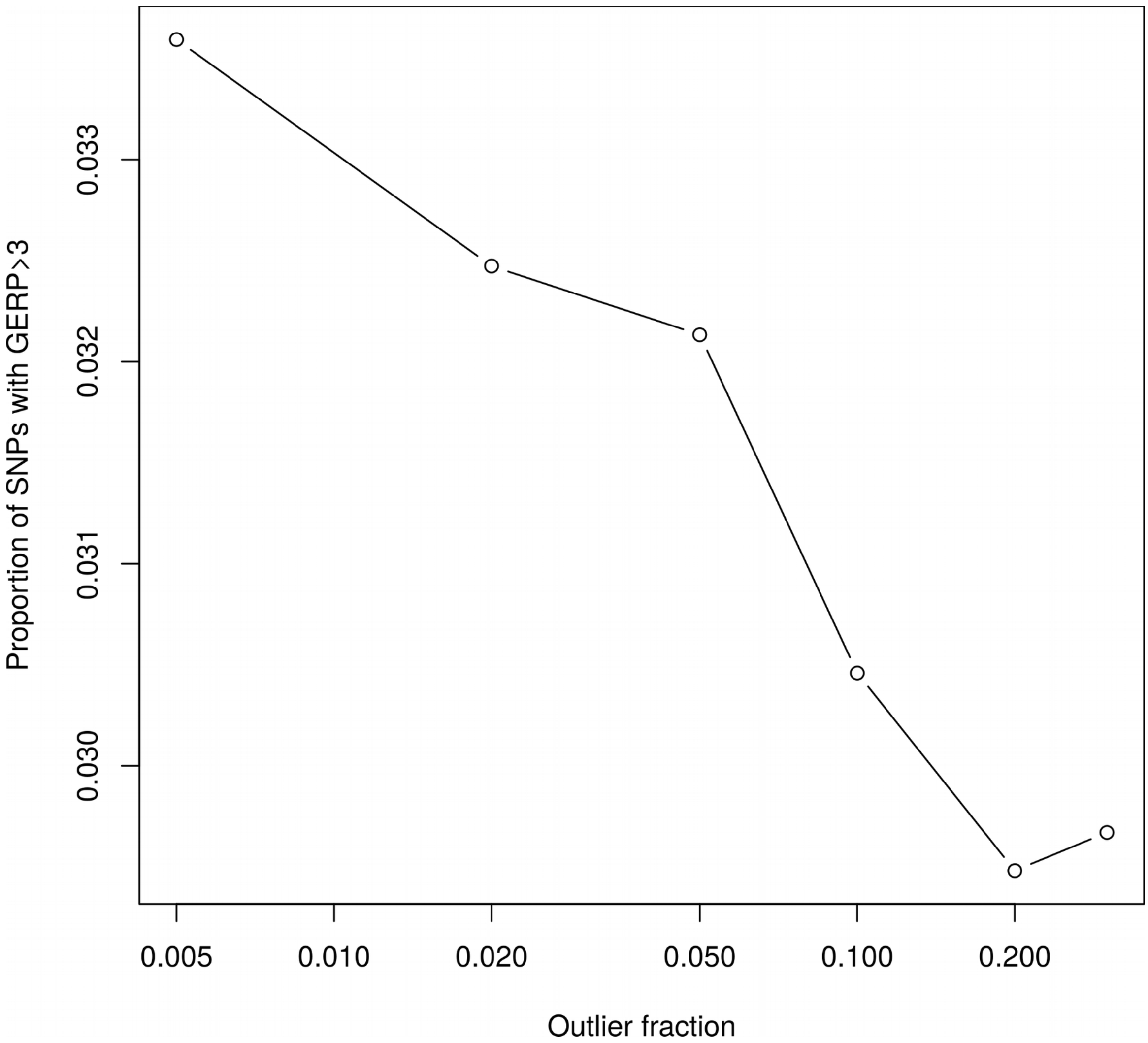

Figure S9.1 Enrichment of SNPs with high conservation among the outliers of the selection scan

We explored the most extreme and positive values of our statistic since those represent SNPs similar in modern-day and Mesolithic northern Europeans, but different to southern Europeans. We used SNPnexus [7] to obtain annotation for the top ranking SNPs in our genome-wide scan. Notably, six of the ten SNPs with the highest D_{sel} values are located in the transmembrane gene *TMEM131* (https://www.ncbi.nlm.nih.gov/gap/phegeni?tab=1&gene=23505). GWAS have associated SNPs in this gene with performance in exercise tests (rs10520549, $p<10^{-5}$ [8]) and heart rate (rs1026015, $p<10^{-5}$ [9]). The heritability of the 17 tested physical exercise phenotypes are in the range 0.30-0.52 [8]. These cardiovascular traits are likely connected to the climatic conditions in northern Europe [4]. Four of the top 100 ranking SNPs (Table S9.1) are located in *FHIT*, which has been associated with a wide range of phenotypes (Table S9.2). These include psychological traits (sleep [10], attention-deficit hyperactivity disorder [11], major depressive disorder [12], Tobacco Use Disorder [13], Asperger Syndrome [14], metabolic traits (body mass index [15], type 2 diabetes [16]), cardiovascular traits (blood pressure [17]), and developmental traits (Cleft Lip [17], menopause [18]). Due to this large range of different phenotypes it is difficult to find a clear link to adaptation to high-latitude climates, although several of the traits involved have been linked to cold adaptation [4]. *GPC5* harbors three of the top 100 ranking SNPs. This gene has also been associated with a wide range of phenotypes,including metabolic traits (serum metabolites [19], Cholesterol and HDL [20]), immune phenotypes (Monocyte Chemoattractant Protein-1 [21], multiple sclerosis [22–24], Crohn's disease

[25]) and developmental traits (Mental Competency [18], height [26], hair thickness [27], kidney aging [28]. For both *GPC5* and *FHIT*, a majority of the phenotypes are possibly involved in local adaptation, e.g. handling changes in light exposure (psychological and developmental traits) and the increased energy demand during cold seasons (metabolic and cardiovascular traits), or general physiological changes to adapt to the environment (developmental traits). The genes *PLD1* and *GABPB1* also harbor three or more SNPs out of the first 100 SNPs of D_{sel}. Unfortunately, we do not find any GWAS results for these genes. *PLD1* is involved in Ras protein signal transduction [29], so it could be connected to the response to external signals. *GABPB1* might be involved in physical performance in competitions [30], which is similar to the associations of *TMEM131* and may also be well connected to the climatic conditions in northern Europe. Other genes among the top 100 SNPs are associated with a wide range of metabolic, cardiovascular and psychological traits (Table S9.2).

All six of the highest scoring SNPs that fell within *TMEM131* show similar allele frequency differences between FIN and TSI which suggests that two different haplotypes are present in high frequencies in these two modern populations. In total, the region comprises at least 264 kilobases with allele frequency differences of up to 40%. This region is a genome-wide outlier in its allele frequency difference between FIN and TSI compared to other regions of similar length (Figure S9.3d). To produce Figure S9.3d we defined blocks as follows: For each SNP, we scanned the next 50 kbp for other SNPs with maximally 5% difference between the allele frequency differences between the two populations. A block is a sequence of such SNPs with less than 50 kbp between neighboring SNPs (note that SNPs with highly different allele frequencies were allowed if another SNP within 50 kbp had a similar allele frequency difference). Figure S9.3d shows the block with the maximum allele frequency per 1 Mbp window of the genome. In order to investigate the haplotype structure in the TMEM131 region, we phased chromosome 2 of all FIN and TSI individuals plus SF12 and Hum2 using FastPHASE 1.4 [31] (with parameters -T25 -C25 -w -Pm -Pp -H100 -K25 -Kp.1). The GNU R package pegas [32] was used to draw a haplotype network for the region (Figure S9.3e). All major haplotype configurations seem to be segregating in both modern populations but there appears to be a clear gradient between the two populations. Both of the most extreme haplotype configurations are predominantly found in either TSI or FIN, and the haplotype found in SF12 and Hum2 is more common in FIN than in TSI. This pattern of haplotype differentiation is also visible in a haplotype bifurcation diagram around the highest scoring SNP in *TMEM131* (Figure S9.3c, drawn using the R package rehh2 [33]). The *TMEM131* region is the second strongest signal of haplotype differentiation on chromosome 2 when using a haplotype based selection scan (rsb [34], calculated with rehh2 [33]). Only the region around the Lactase gene shows a higher haplotype differentiation between TSI and FIN (Figure S9.3a,b). Both alleles at the highly differentiated SNPs are also found in other prehistoric Europeans included in this study. The SNPs are polymorphic in both hunter-gatherers and early farmers, but the haplotype found in sf12 is found in slightly higher frequencies in other hunter-gatherers (e.g. SF9, Steigen, Motala12, Hum1, ajv58, Loschbour) than in early farmers (LBK is a homozygous carrier, while Gok2 and ne1 are homozygous alternative) (Figure S9.4). Notably, the haplotype is also found in a Late Neolithic/Bronze Age Scandinavian (RISE98 [35], Figure S9.4).

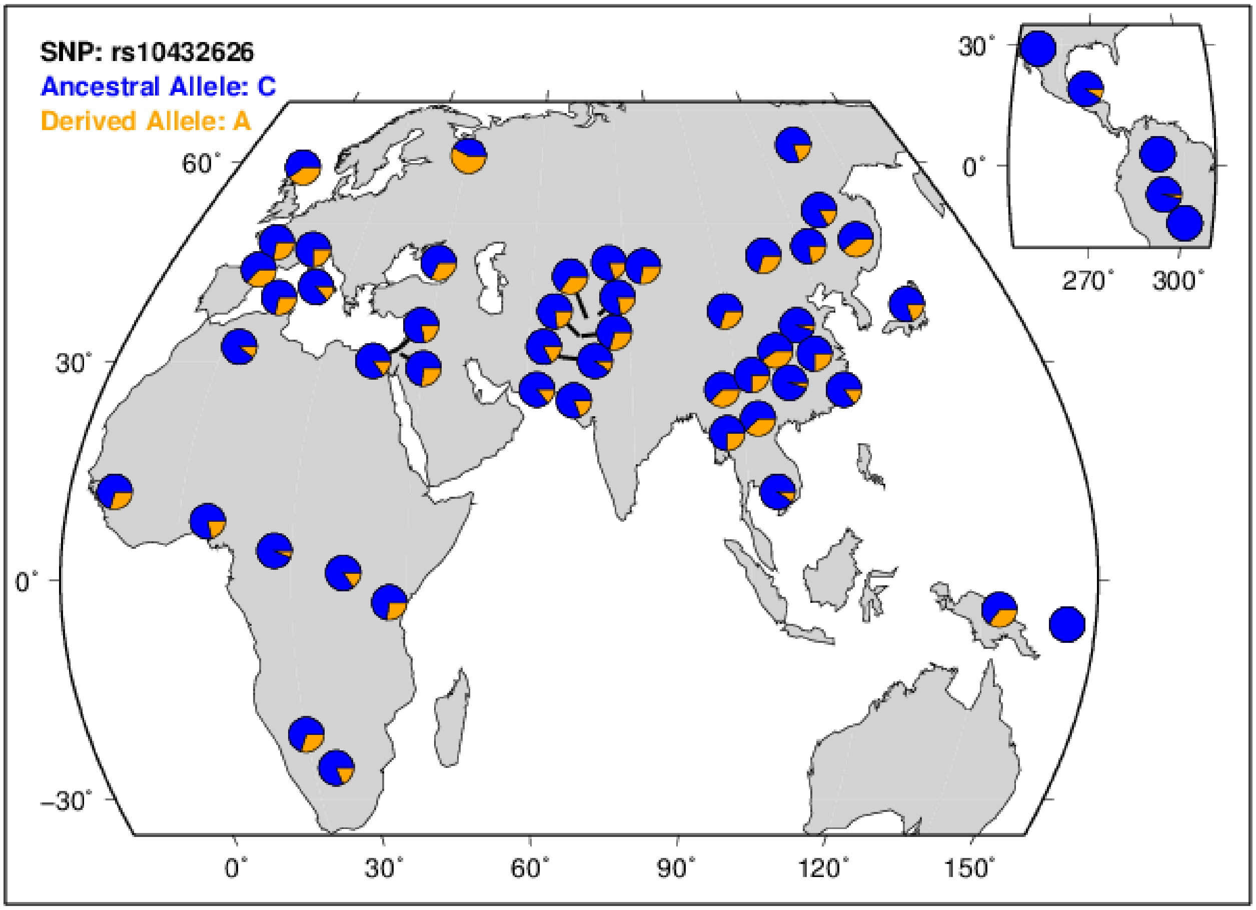

Figure S9.2 Allele frequencies of rs10432626, one of the SNPs in TMEM131. The plot was obtained from the HGDP selection browser (http://hgdp.uchicago.edu/).

In addition to investigating the genes among the top 100 SNPs, we also looked at biological process GO terms among the top 0.5% of D_{sel} scores (1298 SNPs) compared to all SNPs tested. For GO term enrichment we used Gowinda [36]. Gowinda employs a permutation approach to detect GO terms overrepresented among a subset of all SNPs analyzed. This accounts for the different lengths of genes and the number of SNPs expected for each gene. Gowinda was run in gene mode while counting all SNPs 20kbp up- or downstream to that gene. We only used categories with at least 10 genes and conducted 1,000,000 permutations. Only ten GO terms have a FDR of less than 20%, we show the top 20 GO terms in Table S9.3. In contrast to the genes among the top 100 SNPs, these GO terms mainly include developmental processes but also some involved in signaling processes. These could be involved in polygenic adaptation to high-latitide climates and may have changed morphology in northern Europeans as represented by individuals from Pitted War Culture individuals (PWC) in osteological analyses. Comparing individuals from Funnel beaker (TRB) and PWC contexts in Sweden osteologically, a certain degree of morphological differences between the skeletons has been found. It has been shown that PWC individuals exhibit skeletal traits characteristic of cold-adaptation, such as certain facial features and limb proportions (crural index) which are absent in TRB individuals [37,38].

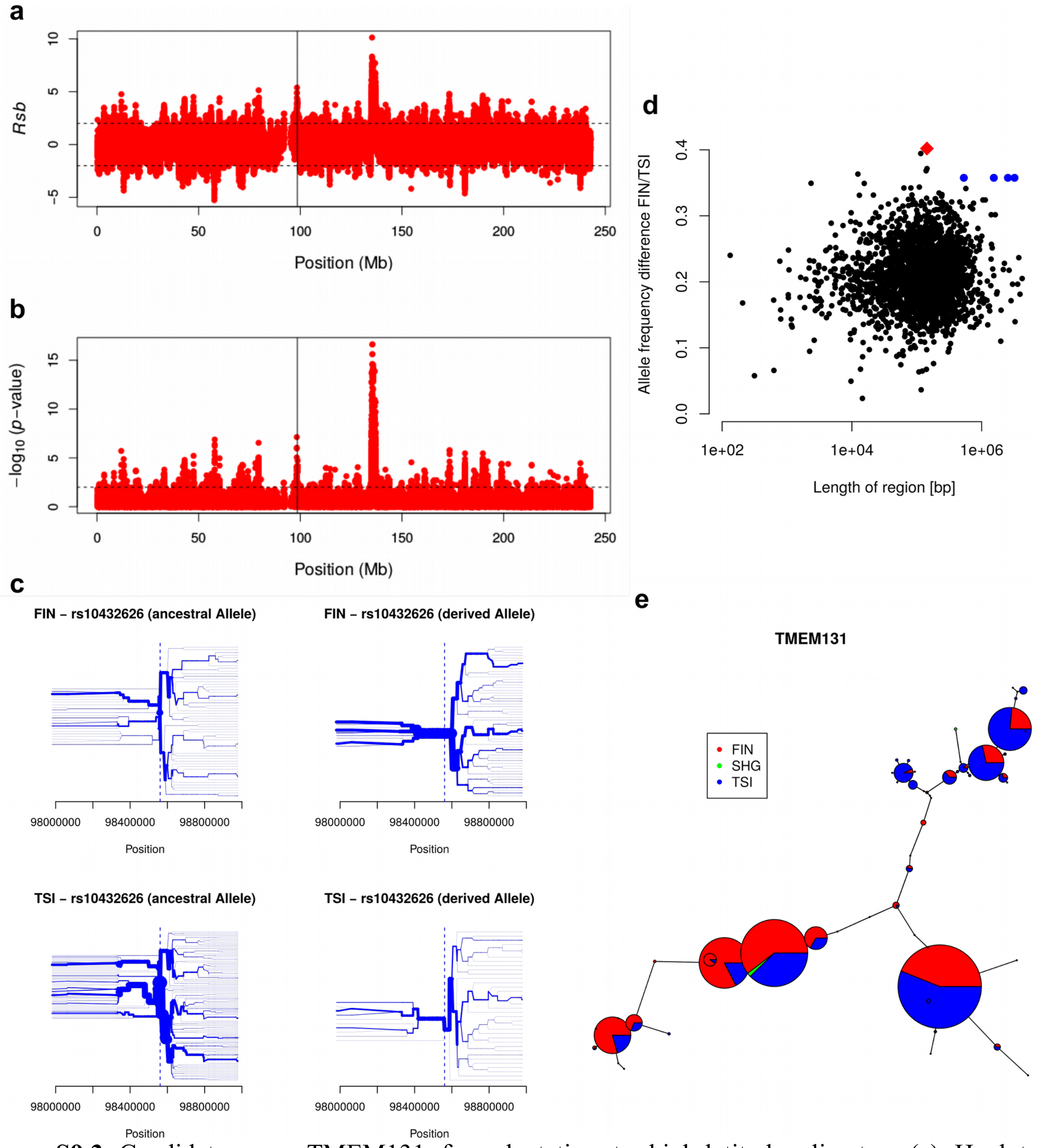

Figure S9.3 Candidate gene TMEM131 for adaptation to high-latitude climates. (a) Haplotype differentiation measured using rsb between TSI and FIN populations on chromosome 2 (calculated with rehh2 , Supplementary Information 10), and (b) -log 10 (p-value) for rsb. (c) Bifurcation of the different haplotypes around the highest scoring SNP (drawn with GNU R and rehh2). (d) Block length versus allele frequency differences between southern Europeans, TSI, and northern Europeans, FIN. Blocks are defined as the maximum physical distance (in base-pairs) between two SNPs of similarly high allele frequency difference between TSI and FIN, requiring that the block contains other SNPs with similarly high allele frequency difference, but with a maximum distance of 50 kbp between neighboring SNPs. The red diamond represents the TMEM131 gene region, blue dots represent the OCA2/HERC region. (e) Haplotype network of the TMEM131 region (drawn with the GNU R package pegas).

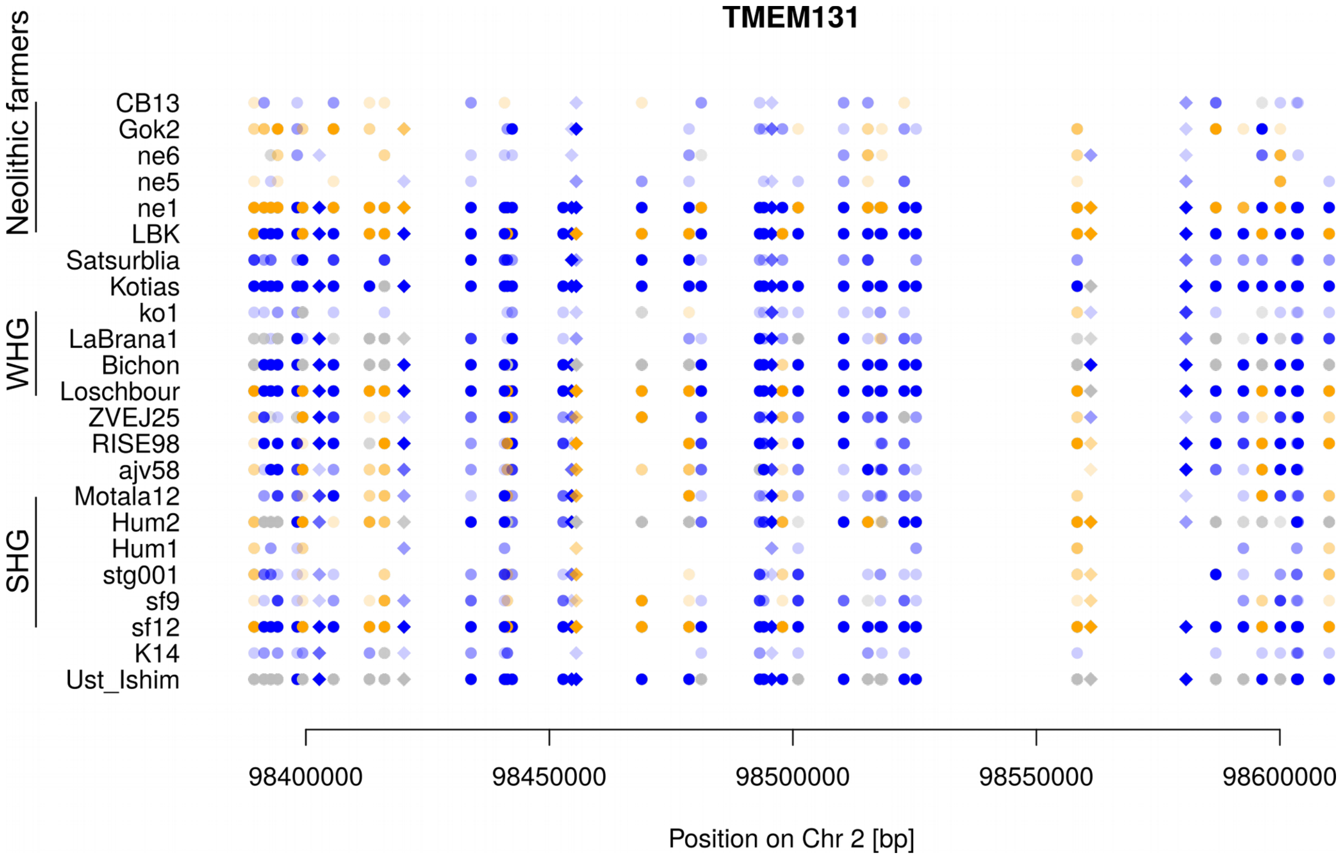

Figure S9.4: TMEM131 haplotype in different ancient individuals. The reference allele is shown in blue, the alternative allele in orange, the SNPs are colored gray if both alleles are observed. The SNPs are shaded according to their coverage, sites covered by 5 or more reads are in full color. Diamonds represent SNPs among the highest ranking Dsel scores.

Table S9.1 Top 100 SNPs of the D_{sel} analysis

SNP-ID	D_{sel}	DAF_{SHG}	DAF_{FIN}	DAF_{TSI}	Consequence type	Gene(s)
rs10432626	0.397	0	0.37	0.77	intronic	*TMEM131*
rs13020776	0.393	0.17	0.37	0.77	intronic	*TMEM131*
rs1838797	0.393	0.83	0.63	0.23	intronic	*TMEM131*
rs10210880	0.382	0	0.15	0.53	intronic	*TMEM131*
rs11692671	0.38	0.83	0.64	0.26	intronic	*ZAP70*
rs7402734	0.368	0.83	0.74	0.37	intergenic	
rs11894541	0.341	0.17	0.15	0.53	intronic	*TMEM131*
rs35940587	0.341	0.17	0.15	0.53	intronic	*TMEM131*
rs168714	0.332	0.83	0.82	0.49	intergenic	
rs6726062	0.325	0.25	0.4	0.73	intronic	*PARD3B*
rs6546608	0.321	0.83	0.74	0.42	intergenic	
rs10276954	0.319	0.61	0.58	0.26	intergenic	
rs537672	0.318	0.43	0.44	0.76	intronic, non-coding intronic	*SLC9A8*
rs6012753	0.317	0.67	0.56	0.24	intronic, non-coding intronic	*SLC9A8*
rs6118443	0.317	0.29	0.32	0.64	intergenic	
rs452203	0.315	0	0.22	0.54	intronic	*FHIT*
rs2734389	0.314	0.08	0.23	0.55	intronic	*FHIT*
rs306169	0.314	0.93	0.7	0.39	intergenic	
rs3104821	0.314	0	0.42	0.74	intergenic	
rs4882475	0.314	0.17	0.24	0.56	intergenic	
rs2218657	0.312	0.83	0.55	0.23	non-coding intronic	*MIR4435_1HG*
rs3112591	0.31	0	0.23	0.54	intergenic	
rs369278	0.304	0.08	0.24	0.55	intronic	*FHIT*

rs4845824	0.304	0.58	0.61	0.26	intergenic	
rs7963463	0.302	0	0.34	0.64	intergenic	
rs11251448	0.3	0.31	0.36	0.66	intergenic	
rs7698798	0.3	0	0.18	0.48	intronic	*MTTP*
rs7173127	0.3	0.17	0.37	0.67	intronic	*GABPB1*
rs11638564	0.3	0.83	0.63	0.33	intronic	*GABPB1*
rs1972701	0.3	1	0.63	0.33	intronic	*GABPB1*
rs1972700	0.3	0	0.37	0.67	intronic	*GABPB1*
rs28372114	0.3	0.14	0.37	0.67	intronic	*GABPB1*
rs11853236	0.3	0.17	0.37	0.67	intronic	*GABPB1*
rs11070768	0.3	0.83	0.63	0.33	intronic	*GABPB1*
rs2033115	0.3	0.14	0.37	0.67	intronic	*GABPB1*
rs11069744	0.299	0.08	0.31	0.61	intergenic	
rs12465488	0.299	0.08	0.43	0.73	intronic	*PARD3B*
rs556682	0.298	0.93	0.8	0.5	intergenic	
rs9571939	0.298	0.94	0.8	0.5	intergenic	
rs3784296	0.298	0.71	0.55	0.25	3’ downstream	*GABPB1*
rs12811599	0.297	0.17	0.28	0.58	intronic	*ANO4*
rs11184569	0.295	0.81	0.76	0.46	intergenic	
rs12034143	0.294	0.83	0.69	0.39	intergenic	
rs6958292	0.294	0.64	0.56	0.26	intergenic	
rs11688847	0.293	0.17	0.2	0.5	intergenic	
rs62256379	0.292	0.75	0.65	0.36	intronic	*SUCLG2*
rs7129877	0.292	0.42	0.47	0.77	intergenic	
rs57090061	0.291	0.25	0.61	0.9	intronic	*PLD1*
rs6806989	0.291	0.93	0.39	0.1	intronic	*PLD1*
rs7616441	0.291	0.44	0.61	0.9	intronic	*PLD1*
rs6773632	0.291	0.42	0.61	0.9	intronic	*PLD1*
rs9839305	0.29	0.79	0.51	0.21	3’ utr	*GPD1L*
rs378022	0.29	0.08	0.25	0.54	intronic	*FHIT*
rs8007792	0.29	0.14	0.19	0.48	intronic	*TTLL5*
rs6434424	0.288	0	0.15	0.44	intergenic	
rs2908871	0.287	0.29	0.41	0.7	intergenic	
rs7145573	0.287	0.75	0.53	0.24	intronic	*ACTN1*
rs6883098	0.285	0.94	0.75	0.47	non-coding intronic	*LOC102467224*
rs7987488	0.285	0	0.25	0.53	intronic	*GPC5*
rs1002420	0.285	0.14	0.25	0.54	intronic	*PCSK5*
rs6598159	0.285	0.71	0.62	0.34	intergenic	
rs2005127	0.284	0.83	0.8	0.51	intergenic	
rs4805487	0.284	0.75	0.55	0.26	intergenic	
rs28679562	0.283	0.57	0.57	0.86	intergenic	
rs79176913	0.283	0.08	0.16	0.44	intergenic	
rs7581814	0.283	0.06	0.16	0.44	intergenic	
rs12230024	0.283	0.69	0.53	0.25	intergenic	
rs2704516	0.282	0.92	0.71	0.43	intergenic	
rs41377545	0.282	1	0.96	0.68	intergenic	
rs7332756	0.282	0.83	0.77	0.49	intronic	*GPC5*
rs10804805	0.281	0.67	0.57	0.29	intergenic	
rs494428	0.281	0	0.25	0.53	intergenic	
rs6492597	0.281	0.17	0.25	0.53	intronic	*GPC5*
rs59740759	0.28	0.17	0.26	0.54	intergenic	
rs41204	0.28	0.1	0.51	0.79	intergenic	
rs62109766	0.279	0.86	0.55	0.27	5’ upstream, intronic	*ARHGEF18*
rs6859099	0.279	0.92	0.73	0.45	non-coding intronic	*LOC102467224*
rs10819439	0.279	0.86	0.79	0.51	intronic	*ZER1*
rs9541386	0.279	0.08	0.15	0.43	intergenic	
rs4869761	0.279	0	0.21	0.49	intronic	*SYNE1*
rs10203341	0.278	0.83	0.72	0.44	intronic	*THSD7B*
rs9521695	0.278	0.25	0.4	0.68	intronic	*COL4A2*

rs2819419	0.278	0.75	0.6	0.32	coding	*AHNAK2*
rs7162536	0.278	0.83	0.65	0.37	intergenic	
rs80353268	0.278	0.75	0.71	0.43	intronic, non-coding intronic	*CCNT2*
rs1319222	0.277	1	0.83	0.56	intronic, 5' upstream	*SEMA5A, SNHG18*
rs793084	0.277	0.5	0.45	0.18	intergenic	
rs4748302	0.277	0.92	0.64	0.36	3' downstream, 3' utr	*PTER, C1QL3*
rs10258475	0.276	0.14	0.24	0.52	intergenic	
rs7296207	0.276	0.21	0.37	0.64	intergenic	
rs1300237	0.276	0	0.37	0.64	intronic	*SLC46A3*
rs11221793	0.275	0	0.13	0.41	intergenic	
rs9884570	0.275	0.25	0.38	0.66	intronic	*DCHS2*
rs6067275	0.275	0.17	0.38	0.66	intergenic	
rs7958156	0.275	0	0.32	0.6	intergenic	
rs7631636	0.275	0.42	0.45	0.72	intronic	*SUCLG2*
rs9419673	0.274	0.88	0.73	0.46	intergenic	
rs7213892	0.274	0.93	0.48	0.21	intronic	*ALOXE3*
rs17050803	0.274	0.33	0.33	0.61	intergenic	
rs28647713	0.274	1	0.67	0.39	intergenic	

Table S9.2 GWAS results for the genes found among the top 100 SNPs of the D_{sel} analysis.

**Gene**	**GWAS associated phenotype**
PCSK5	Dehydroepiandrosterone, Body Height
PTPRN2	C-Reactive Protein
THSD7B	Brain, Cholesterol, HDL, Cholesterol, LDL
TPO	Respiratory Function Tests
AFF3	Cholesterol, Cholesterol, HDL
TMEM131	Exercise Test, Heart Rate
MLL3	Schizophrenia
CNTNAP2	Heart Failure
IRG1	Waist Circumference
SPEN	Heart Failure
EPHB2	Insulin, Insulin Resistance
SEPT10	Blood Pressure
PDE4DIP	Respiratory Function Tests
AGBL3	Attention Deficit Disorder with Hyperactivity
MTF1	Hypothyroidism
NAV2	Arteries, Asthma, Cell Adhesion Molecules, Lipoproteins, Myocardial Infarction, Stroke, Attention Deficit Disorder with Hyperactivity, HIV-1
FAM23A	Blood Coagulation Factors, Body Weight
MRC1L1	Aspartate Aminotransferases
MRC1	Aspartate Aminotransferases
TPH2	Waist Circumference
PUS7	Erythrocyte Indices
PARD3B	Knee osteoarthritis, C-Reactive Protein, Platelet Count, Cholesterol, HDL, Body Height, Osteoarthritis, Knee, E-Selectin, Tuberculosis, Acquired Immunodeficiency Syndrome
SLC26A5	Triglycerides
SEMA5A	Autism, Parkinson's disease, Blood Pressure Determination, Breath Tests, Glucose, Myocardial Infarction, Tunica Media, Parkinson Disease, Alkaline Phosphatase, Peroxidase, Mortality, Hip, Hemoglobin A, Glycosylated, Cholesterol, Cholesterol, LDL, Body Weight, Blood Pressure, Carotid Artery Diseases
HP	Apolipoproteins B, Cholesterol, LDL
HPR	Apolipoproteins B, Cholesterol, LDL
GC	Erythrocytes, Vitamin D
ANK3	Arteries, Creatinine, Glomerular Filtration Rate, Cholesterol, LDL, Triglycerides, Bipolar Disorder, Schizophrenia
ZNF32	Body Mass Index
RET	Hirschsprung Disease
FHIT	lung cancer and preneoplastic bronchial lesions, tumour kinetics and chromosomal instability, transcriptional inactivation of the FHIT gene, smoking, cervical cancer, prostate cancer, ADHD | attention-deficit hyperactivity disorder, major depressive disorder , Albumins, Body Composition, Coronary Artery Disease, Erythrocyte Count, Lipids, Lipoproteins, Myocardial Infarction, Schizophrenia, Stroke, Waist Circumference, Creatinine, Glomerular Filtration Rate, Fibrinogen, Body Mass Index, Body Weight, Blood Pressure, Sleep, Asperger Syndrome, Aorta, Anticonvulsants, Cleft Lip
LRRN1	Blood Pressure, Menopause, Cholesterol, HDL, Triglycerides, Body Weight, Echocardiography
TKT	Waist Circumference, Heart Function Tests
ZNF717	Hippocampus
IGHG1	Sjogren's syndrome, atopy
ANG	Stroke
ABCB1	Phospholipids
TNP2	Diabetes Mellitus, Type 1
C7orf10	Precursor Cell Lymphoblastic Leukemia-Lymphoma
ZNF107	Calcium
UNC13A	Hemoglobins, Amyotrophic Lateral Sclerosis
ZNF92	Smoking
ZNF138	Smoking
BMP8A	Atrial Natriuretic Factor
BMP8B	Atrial Natriuretic Factor
IL12RB2	Liver Cirrhosis, Biliary
SYNE1	Ovarian cancer , tonometry, Body Height, Erythrocyte Count, Forced Vital Capacity, Diabetes Mellitus, Type 2, Triglycerides, Echocardiography
PIGF	Body Height

STK4	Neuroblastoma
LRRN4	Menopause
TGM6	Stroke
EMR2	Blood Pressure Determination
EMR3	Blood Pressure Determination
GPC5	Serum metabolites, multiple sclerosis, height, Coronary Artery Disease, Glucose, Monocyte Chemoattractant Protein-1, Mental Competency, Cholesterol, HDL, Echocardiography, Lung Neoplasms, Nephrotic Syndrome
DAO	Erythrocyte Count, Hemoglobins
SF1	Gout
ACTN1	Arteries
TRIM16	Hemoglobin A, Glycosylated
COX10	Echocardiography
MTTP	Plasma cholesterol levels and body mass index, ApoB-48, lipid metabolism disorders, diabetes, type 2, blood pressure, arterial, steatohepatitis, non-alcoholic, body mass; cholesterol, LDL; cholesterol, total; insulin; apoB, atherosclerosis, coronary; lipoprotein; lipids, blood pressure, arterial diabetes, type 2 glucose insulin, Fatty Liver| Hepatitis C, Chronic
TEC	Inflammatory Bowel Diseases
NRAS	Erythrocytes
MPP7	Iron, Body Mass Index, Echocardiography, Cardiovascular Diseases, Electrocardiography, Alzheimer Disease, Asthma
TTLL5	Body Height
CPN1	Iron, Alkaline Phosphatase
LIPA	Coronary Artery Disease
AGK	Dehydroepiandrosterone Sulfate
COL4A2	Coronary Artery Disease, Vascular Calcification
COLQ	Alcoholism, Body Height, Iron
DCHS2	C-Reactive Protein, Lipoproteins, Blood Coagulation Factors, Erythrocytes, Lipids, Triglycerides, Blood Pressure, Fibrinogen, Alzheimer Disease

Table S9.3 Results of the GO-term enrichment analysis.

GO-ID	Total number of genes	Expected number of genes among outliers	Observed number of genes among outliers	Nominal p-value	FDR	Description of GO term
GO:0060603	37	1.707	9	0.000018	0.029482	mammary gland duct morphogenesis
GO:0060443	53	2.48	10	0.000061	0.0506862	mammary gland morphogenesis
GO:0022612	109	4.591	14	0.000099	0.0506862	gland morphogenesis
GO:0060444	25	0.905	6	0.000134	0.0506862	branching involved in mammary gland duct morphogenesis
GO:0021536	63	2.437	9	0.000146	0.0506862	diencephalon development
GO:0061180	68	2.919	10	0.000299	0.0849803333	mammary gland epithelium development
GO:0071514	22	0.701	5	0.000466	0.1161662857	genetic imprinting
GO:0030879	127	5.523	14	0.000735	0.1490481111	mammary gland development
GO:0048732	266	10.015	21	0.00074	0.1490481111	gland development
GO:0048589	265	12.449	24	0.000866	0.1562793	developmental growth
GO:0033135	62	2.321	8	0.001343	0.2200139091	regulation of peptidyl-serine phosphorylation
GO:0050432	30	0.935	5	0.001592	0.2226172632	catecholamine secretion
GO:0072077	18	0.617	4	0.001716	0.2226172632	renal vesicle morphogenesis
GO:0035023	165	7.987	17	0.001935	0.2226172632	regulation of Rho protein signal transduction
GO:0006885	46	1.415	6	0.002005	0.2226172632	regulation of pH
GO:0045740	48	1.368	6	0.00209	0.2226172632	positive regulation of DNA replication
GO:0051926	21	0.599	4	0.002166	0.2226172632	negative regulation of calcium ion transport
GO:0006655	10	0.295	3	0.002262	0.2226172632	phosphatidylglycerol biosynthetic process
GO:0040019	8	0.431	3	0.002278	0.2226172632	positive regulation of embryonic development
GO:0048754	143	4.78	12	0.002602	0.24296675	branching morphogenesis of an epithelial tube

S9.2 Testing selection on known pigmentation SNPs

To complement the genome-wide scan above, we specifically looked into signals of selection in known pigmentation-associated SNPs as pigmentation is one of the major traits under selection pressure, especially in high latitudes [39]. Pigmentation is a trait well studied in populations of European descent (see also S8 Text). Here we focus on three major-effect SNPs in the genes *OCA2/HERC2* affecting eye pigmentation, and *SLC45A2* as well as *SLC24A5* affecting skin pigmentation. We observe (Figure 4B) that the allele frequencies of the derived allele at all three SNPs is higher in SHGs than expected based on their genome-wide admixture proportions (qpAdm estimates; S6 Text) and the allele frequencies in EHGs and WHGs. To test whether these allele frequency changes are significant, we performed simulations. For each SNP and each SHG individual, we randomly sampled the alleles from the two source populations based on the individual's genome-wide qpAdm admixture proportions and the allele frequencies in the source populations. The allele frequencies in the source populations were calculated as described in S8 Text. We assume that the true frequencies in the source populations follow a normal distribution with mean as our point estimate and standard deviation as the binomial sampling error estimated from a normal approximation:

$$SE=\sqrt{\frac{\hat{p}(1-\hat{p})}{n}}$$

Where $\hat{p}$ is the point estimate of the allele and n is the number of chromosomes. This approximation can be inaccurate if the allele frequency estimate is close to 0 or 1. Therefore, we take the conservative choice of always using the maximum standard error possible for a given sample size which is reached when p=0.5. This approach will overestimate the uncertainty in the source populations' allele frequencies in most cases but it avoids underestimating the uncertainty in the situations where allele frequency estimates are close to 0 or 1.

The true admixture proportions per individual are also drawn from a normal distribution with mean equal to the point estimate and standard deviation equal to the jackknife standard error of that estimate. Before calculating allele frequencies in the admixed SHGs, we randomly sample the same number of SHGs for which data was available in the empirical study to account for noise due to missing data. This simulation is assumed to provide a null distribution of SHG allele frequencies without selection. After 1,000,000 simulations, we find that the allele frequencies in *SLC45A2* (p=0.076862), *OCA2/HERC2* (p=0.060368) and *SLC24A5* (p=0.180055) are elevated but not significantly. These p values may be overestimated since our simulations can be considered conservative. As all three of them are pointing in the same direction and as the three SNPs can be considered evolutionary independent, we calculated a combined p value. We used Fisher's method [40] to combine the three p values and the p value for observing all three SNPs elevated like this is 0.028. The results of this simulation are shown in Figure 4B.

These results suggest that high latitude conditions exhibited a selection pressure on pigmentation phenotypes in SHGs. The polygenic architecture of skin pigmentation as well as the occurrence of different combinations of depigmentation mutations in different parts of the world suggests that selection on skin pigmentation is mainly due to physiological advantages of light pigmentation in high latitudes [41]. Hair and eye-color pigmentation on the other hand could have been affected by drift and sexual selection as less mutations need to be involved [41].

References

1. Skoglund P, Malmstrom H, Omrak A, Raghavan M, Valdiosera C, Gunther T, et al. Genomic Diversity and Admixture Differs for Stone-Age Scandinavian Foragers and Farmers. Science. 2014;344: 747–750. doi:10.1126/science.1253448

2. Lazaridis I, Patterson N, Mittnik A, Renaud G, Mallick S, Kirsanow K, et al. Ancient human genomes suggest three ancestral populations for present-day Europeans. Nature. 2014;513: 409–413. doi:10.1038/nature13673

3. Skoglund P, Malmstrom H, Raghavan M, Stora J, Hall P, Willerslev E, et al. Origins and Genetic Legacy of Neolithic Farmers and Hunter-Gatherers in Europe. Science. 2012;336: 466–469. doi:10.1126/science.1216304

4. Makinen TM. Different types of cold adaptation in humans. Frontiers in bioscience. 2010;2: 1047–1067. doi:10.2741/S117

5. Fumagalli M, Moltke I, Grarup N, Racimo F, Bjerregaard P, Jørgensen ME, et al. Greenlandic Inuit show genetic signatures of diet and climate adaptation. Science (New York, NY). 2015;349: 1343–7. doi:10.1126/science.aab2319

6. Cooper GM. Distribution and intensity of constraint in mammalian genomic sequence. Genome Research. 2005;15: 901–913. doi:10.1101/gr.3577405

7. Ullah AZD, Lemoine NR, Chelala C. SNPnexus: a web server for functional annotation of novel and publicly known genetic variants (2012 update). Nucleic Acids Research. 2012; gks364. doi:10.1093/nar/gks364

8. Vasan RS, Larson MG, Aragam J, Wang TJ, Mitchell GF, Kathiresan S, et al. Genome-wide association of echocardiographic dimensions, brachial artery endothelial function and treadmill exercise responses in the Framingham Heart Study. BMC Medical Genetics. 2007;8: S2. doi:10.1186/1471-2350-8-S1-S2

9. Newton-Cheh C, Guo C-Y, Wang TJ, O'donnell CJ, Levy D, Larson MG. Genome-wide association study of electrocardiographic and heart rate variability traits: the Framingham Heart Study. BMC medical genetics. 2007;8: 1.

10. Gottlieb DJ, T O'Connor G, Wilk JB. Genome-wide association of sleep and circadian phenotypes. BMC medical genetics. 2007;8: 1.

11. Lasky-Su J, Neale BM, Franke B, Anney RJ, Zhou K, Maller JB, et al. Genome-wide association scan of quantitative traits for attention deficit hyperactivity disorder identifies novel associations and confirms candidate gene associations. American Journal of Medical Genetics Part B: Neuropsychiatric Genetics. 2008;147: 1345–1354.

12. Shi M, Murray JC, Marazita ML, Munger RG, Ruczinski I, Hetmanski JB, et al. Genome wide study of maternal and parent-of-origin effects on the etiology of orofacial clefts. American Journal of Medical Genetics Part A. 2012;158: 784–794.

13. Rose JE, Behm FM, Drgon T, Johnson C, Uhl GR. Personalized smoking cessation: interactions between nicotine dose, dependence and quit-success genotype score. Molecular Medicine (Cambridge, Mass). 2010;16: 247–253. doi:10.2119/molmed.2009.00159

14. Salyakina D, Ma DQ, Jaworski JM, Konidari I, Whitehead PL, Henson R, et al. Variants in several genomic regions associated with asperger disorder. Autism Research. 2010;3: 303–310.

15. Fox CS, Heard-Costa N, Cupples LA, Dupuis J, Vasan RS, Atwood LD. Genome-wide association to body mass index and waist circumference: the Framingham Heart Study 100K project. BMC medical genetics. 2007;8: 1.

16. Bailey CJ, Gross JL, Pieters A, Bastien A, List JF. Effect of dapagliflozin in patients with type 2 diabetes who have inadequate glycaemic control with metformin: a randomised, double-blind, placebo-controlled trial. The Lancet. 2010;375: 2223–2233.

17. Levy D, Larson MG, Benjamin EJ, Newton-Cheh C, Wang TJ, Hwang S-J, et al. Framingham Heart Study 100K Project: genome-wide associations for blood pressure and arterial stiffness. BMC medical genetics. 2007;8: 1.

18. Lunetta KL, D'Agostino RB, Karasik D, Benjamin EJ, Guo C-Y, Govindaraju R, et al. Genetic correlates of longevity and selected age-related phenotypes: a genome-wide association study in the Framingham Study. BMC medical genetics. 2007;8: 1.

19. Gieger C, Geistlinger L, Altmaier E, De Angelis MH, Kronenberg F, Meitinger T, et al. Genetics meets metabolomics: a genome-wide association study of metabolite profiles in human serum. PLoS Genet. 2008;4: e1000282.

20. Kathiresan S, Manning AK, Demissie S, D'agostino RB, Surti A, Guiducci C, et al. A genome-wide association study for blood lipid phenotypes in the Framingham Heart Study. BMC medical genetics. 2007;8: 1.

21. Benjamin EJ, Dupuis J, Larson MG, Lunetta KL, Booth SL, Govindaraju DR, et al. Genome-wide association with select biomarker traits in the Framingham Heart Study. BMC medical genetics. 2007;8: 1.

22. Baranzini SE, Wang J, Gibson RA, Galwey N, Naegelin Y, Barkhof F, et al. Genome-wide association analysis of susceptibility and clinical phenotype in multiple sclerosis. Human Molecular Genetics. 2009;18: 767–778. doi:10.1093/hmg/ddn388

23. Mero I-L, Lorentzen $\backslash$AAslaug R., Ban M, Smestad C, Celius EG, Aarseth JH, et al. A rare variant of the TYK2 gene is confirmed to be associated with multiple sclerosis. European journal of human genetics. 2010;18: 502–504.

24. Gourraud P-A, McElroy JP, Caillier SJ, Johnson BA, Santaniello A, Hauser SL, et al. Aggregation of multiple sclerosis genetic risk variants in multiple and single case families. Annals of neurology. 2011;69: 65–74.

25. Cleynen I, John JMM, Henckaerts L, van Moerkercke W, Rutgeerts P, van Steen K, et al. Molecular reclassification of Crohn's disease by cluster analysis of genetic variants. PLoS ONE. 2010;5. doi:10.1371/journal.pone.0012952

26. Okada Y, Kamatani Y, Takahashi A, Matsuda K, Hosono N, Ohmiya H, et al. A genome-wide association study in 19 633 Japanese subjects identified LHX3-QSOX2 and IGF1 as adult height loci. Human molecular genetics. 2010; ddq091.

27. Fujimoto A, Nishida N, Kimura R, Miyagawa T, Yuliwulandari R, Batubara L, et al. FGFR2 is associated with hair thickness in Asian populations. Journal of human genetics. 2009;54: 461–465.

28. Wheeler HE, Metter EJ, Tanaka T, Absher D, Higgins J, Zahn JM, et al. Sequential use of transcriptional profiling, expression quantitative trait mapping, and gene association implicates MMP20 in human kidney aging. PLoS Genet. 2009;5: e1000685.

29. Suzuki J, Yamazaki Y, Li G, Kaziro Y, Koide H, Guang L. Involvement of Ras and Ral in chemotactic migration of skeletal myoblasts. Mol Cell Biol. 2000;20: 4658–4665.

30. Eynon N, Alves AJ, Sagiv M, Yamin C, Sagiv M, Meckel Y. Interaction between SNPs in the NRF2 gene and elite endurance performance. Physiol Genomics. 2010;41: 78–81. doi:10.1152/physiolgenomics.00199.2009

31. Scheet P, Stephens M. A fast and flexible statistical model for large-scale population genotype data: applications to inferring missing genotypes and haplotypic phase. The American Journal of Human Genetics. 2006;78: 629–644.

32. Paradis E. pegas: an R package for population genetics with an integrated–modular approach. Bioinformatics. 2010;26: 419–420.

33. Gautier M, Klassmann A, Vitalis R. rehh 2.0: a reimplementation of the R package rehh to detect positive selection from haplotype structure. Mol Ecol Resour. 2017;17: 78–90. doi:10.1111/1755-0998.12634

34. Tang K, Thornton KR, Stoneking M. A new approach for using genome scans to detect recent positive selection in the human genome. PLoS biology. 2007;5: e171.

35. Allentoft ME, Sikora M, Sjögren K-G, Rasmussen S, Rasmussen M, Stenderup J, et al. Population genomics of Bronze Age Eurasia. Nature. 2015;522: 167–172. doi:10.1038/nature14507

36. Kofler R, Schlötterer C. Gowinda: unbiased analysis of gene set enrichment for genome-wide association studies. Bioinformatics. 2012;28: 2084–2085.

37. Ahlström T. Den exogama gränsen: Kring interaktionen mellan jägare-samlare och bönder-boskapsskötare under mellanneolitisk tid. Till Gunborg: arkeologiska samtal. 1997;

38. Ahlström T. Pitted ware skeletons and boreal temperatures. Lund Archaeological Review,(3). 1997; 37–48.

39. Fan S, Hansen MEB, Lo Y, Tishkoff SA. Going global by adapting local: A review of recent human adaptation. Science. 2016;354: 54–59. doi:10.1126/science.aaf5098

40. Fisher RA. Statistical methods for research workers. Edinburgh: Oliver and Boyd; 1925.

41. Jablonski NG, Chaplin G. The colours of humanity: the evolution of pigmentation in the human lineage. Philosophical Transactions of the Royal Society B: Biological Sciences. 2017;372: 20160349. doi:10.1098/rstb.2016.0349
